# Vacuolar ATPase Is a Possible Therapeutic Target in Acute Myeloid Leukemia: Focus on Patient Heterogeneity and Treatment Toxicity

**DOI:** 10.3390/jcm12175546

**Published:** 2023-08-25

**Authors:** Sushma Bartaula-Brevik, Calum Leitch, Maria Hernandez-Valladares, Elise Aasebø, Frode S. Berven, Frode Selheim, Annette K. Brenner, Kristin Paulsen Rye, Marie Hagen, Håkon Reikvam, Emmet McCormack, Øystein Bruserud, Tor Henrik Anderson Tvedt

**Affiliations:** 1 Acute Leukemia Research Group, Department of Clinical Science, University of Bergen, 5021 Bergen, Norway; sbartaulabrevik@gmail.com (S.B.-B.); mariahv@ugr.es (M.H.-V.); elise.aasebo@uib.no (E.A.); annette.brenner@uib.no (A.K.B.); kristin.rye@uib.no (K.P.R.); marie.hagen@uib.no (M.H.); hakon.reikvam@uib.no (H.R.); totved@ous-hf.no (T.H.A.T.); 2 Department of Clinical Science, Centre for Pharmacy, University of Bergen, 5015 Bergen, Norway; calum.leitch@uib.no (C.L.); emmet.mc.cormack@uib.no (E.M.); 3 The Proteomics Facility of the University of Bergen (PROBE), University of Bergen, 5009 Bergen, Norway; frode.berven@uib.no (F.S.B.); frode.selheim@uib.no (F.S.); 4 The Department of Biomedicine, University of Bergen, 5009 Bergen, Norway; 5 Department of Physical Chemistry, University of Granada, Avenida de la Fuente Nueva S/N, 18071 Granada, Spain; 6 Instituto de Investigación Biosanitaria ibs.GRANADA, 18012 Granada, Spain; 7 Section for Hematology, Department of Medicine, Haukeland University Hospital, 5021 Bergen, Norway

**Keywords:** acute myeloid leukemia, vacuolar ATPase, bafilomycin A1, concanamycin, proliferation, apoptosis, cytokine, casein kinase 2, toxicity

## Abstract

Vacuolar ATPase (V-ATPase) is regarded as a possible target in cancer treatment. It is expressed in primary acute myeloid leukemia cells (AML), but the expression varies between patients and is highest for patients with a favorable prognosis after intensive chemotherapy. We therefore investigated the functional effects of two V-ATPase inhibitors (bafilomycin A1, concanamycin A) for primary AML cells derived from 80 consecutive patients. The V-ATPase inhibitors showed dose-dependent antiproliferative and proapoptotic effects that varied considerably between patients. A proteomic comparison of primary AML cells showing weak versus strong antiproliferative effects of V-ATPase inhibition showed a differential expression of proteins involved in intracellular transport/cytoskeleton functions, and an equivalent phosphoproteomic comparison showed a differential expression of proteins that regulate RNA processing/function together with increased activity of casein kinase 2. Patients with secondary AML, i.e., a heterogeneous subset with generally adverse prognosis and previous cytotoxic therapy, myeloproliferative neoplasia or myelodysplastic syndrome, were characterized by a strong antiproliferative effect of V-ATPase inhibition and also by a specific mRNA expression profile of V-ATPase interactome proteins. Furthermore, the V-ATPase inhibition altered the constitutive extracellular release of several soluble mediators (e.g., chemokines, interleukins, proteases, protease inhibitors), and increased mediator levels in the presence of AML-supporting bone marrow mesenchymal stem cells was then observed, especially for patients with secondary AML. Finally, animal studies suggested that the V-ATPase inhibitor bafilomycin had limited toxicity, even when combined with cytarabine. To conclude, V-ATPase inhibition has antileukemic effects in AML, but this effect varies between patients.

## 1. Introduction

Acute myeloid leukemia (AML) is a heterogeneous and aggressive malignancy characterized by proliferation of transformed immature hematopoietic cells in the bone marrow [1,2]. The overall long-term AML-free survival is only 40–50%, even for younger patients who can receive the most intensive chemotherapy (possibly combined with allogeneic stem cell transplantation) [2]. However, the large group of elderly and unfit patients cannot receive intensive treatment; instead, many of them only receive leukemia-stabilizing therapy and survive for less than one year [1,2]. Thus, there is a need for new therapeutic strategies both to increase the efficiency of the intensive treatment and to prolong the survival for elderly/unfit patients who only receive stabilizing treatment.

Vacuolar ATPase (V-ATPase) is a proton pump that is regarded as a possible therapeutic target in human malignancies, and the available evidence suggests that this strategy has effects in various solid tumors and possibly hematological malignancies [3,4,5,6,7,8,9,10]. V-ATPases are present both in intracellular membranes (e.g., lysosomes, endosomes, secretory vesicles) and in the plasma membrane, and they are involved in a wide range of physiological processes in normal and malignant cells [4,5,6,7,8,9,10]. Thus, the V-ATPases use energy from hydrolysis of ATP to ADP to pump protons across membranes, and ATP is thereby transported out of the cytosol and into the lumen of various intracellular compartments or into the extracellular space. Proton gradients across both lysosomal and secretory vesicle membranes drive the coupled transmembrane transport of several small molecules, including ions and nutrients, and they are thereby important for intracellular transport and trafficking, proteolytic processing of promediators, receptor-mediated endocytosis and intracellular ligand-receptor dissociation with receptor recycling [4,5,6,7,8,9,10].

The expression of V-ATPase in the plasma membrane is not only important for stabilization of pH gradients but also for the molecular organization of the membrane, e.g., formation of lipid rafts, interactions with caveola and regulation of receptor signaling and recycling [11,12,13,14]. V-ATPase supports migration [15,16], invasion and/or chemoresistance in several human malignancies, including breast cancer, oral squamous cell carcinoma, hepatocellular and pancreatic carcinoma, lung cancer, esophageal cancer and sarcomas [8,17]. However, experimental studies have shown that V-ATPase inhibition may even upregulate certain intracellular prosurvival signals, including upregulation of cholesterol-associated genes [18,19].

Bafilomycins (including the A1, B, C and D forms) are macrolide antibiotics [20,21,22,23,24,25,26,27] that inhibit V-type ATPases [23,28]. Bafilomycin A1 thereby prevents the fusion between lysosomes and autophagosomes [20,24,28,29]. Previous in vitro studies of neuronal cells suggest that this organellar fusion is not observed at concentrations ≤ 1 nM but only at concentrations ≥ 10 nM, and autophagosome accumulation together with induction of apoptosis is then observed for these higher concentrations [20,26]. Chloroquine is a lysosomotrophic agent that compromises normal lysosomal degradation and induces apoptosis even in human AML cells [30,31,32,33,34], and bafilomycin seems to inhibit this chloroquine effect [20,26,35]. Furthermore, concanamycin A is another macrolide with antiproliferative effects in normal human cells [21]. Bafilomycins and concanamycins are regarded as structurally related molecules that inhibit V-ATPase at nanomolar concentrations, whereas P-ATPase is inhibited only at micromolar concentrations of these two agents [20,21,25].

A recent proteomic study suggested that low expression of V-ATPase is associated with clinical chemoresistance in AML (i.e., high risk of leukemia relapse) [3,35], and two recent small experimental studies of primary AML cells suggest that V-ATPase inhibition has anti-AML effects [28,30]. Therefore, in the present study, we have further investigated the functional in vitro effects of the two well-characterized V-ATPase inhibitors bafilomycin A1 and concanamycin A on proliferation and survival of primary human AML cells derived from a large group of consecutive/unselected patients. Our experimental studies included in vitro effects of V-ATPase inhibition on AML cell proliferation (Section 3.1, Section 3.2, Section 3.3, Section 3.4 and Section 3.5), mRNA expression of the V-ATPase interactome (Section 3.6), effects on regulation of apoptosis (Section 3.7), constitutive extracellular mediator release (Section 3.8 and Section 3.9) and communication with neighboring stromal cells (Section 3.10 and Section 3.11), as well as the toxic (Section 3.12) and potential antileukemic effects (Section 3.13) effects of V-ATPase inhibition in animal models. As described above V-ATPase inhibition may have both pro- and anti-apoptotic effects [17,18,19]; for this reason, we focus on patient heterogeneity together with treatment toxicity.

## 2. Materials and Methods

### 2.1. Primary AML Cells

The local Ethics Committee (Regional Ethics Committee, University of Bergen, Bergen, Norway) approved the study (REK Vest 2017/305 14022017), and all patient samples were collected after written informed consent. AML blasts were derived from 80 consecutive patients (35 females, 45 males; median age 64 years). Our department is responsible for AML therapy in a defined geographical area, and our patient inclusion should therefore be regarded as population-based. The clinical and biological characteristics of the patients and their leukemia cells are summarized in Table 1, and the characteristics of the individual patients are presented in Table S1. Enriched AML cells were isolated from peripheral blood by density gradient separation (Lymphoprep; Axis-Shield, Oslo, Norway; specific density 1.077 g/mL); all patients had a relatively high percentage and concentration of circulating leukemia cells and the AML cell populations therefore contained at least 95% blasts [36,37,38]. The cells were cryopreserved and stored in liquid nitrogen until used in the experiments.

### 2.2. Reagents

The medium used for suspension culture of AML cells alone was serum-free Stem Span SFEM (StemSpan™ SFEM; Stem Cell Technologies, Vancouver, BC, Canada). Concanamycin A (CCA), Bafilomycin A1 (BAF) and chloroquine for the in vitro experiments were purchased from Sigma-Aldrich (St. Louis, MO, USA). The two drugs were initially dissolved in DMSO and further diluted in culture medium.

AML cells derived from 6 patients and showing detectable cytokine-dependent proliferation were investigated in dose–response experiments by using the ^3^H-thymidine incorporation assay described below (Section 2.3). In these pilot dose–response experiments, both drugs were tested at final concentrations of 100, 50, 20, 15, 10, 5 and 1 nM. An antiproliferative effect was observed for the majority of patients when the drugs were tested at concentrations ≥10 nM, corresponding to at least 20% inhibition or a relative response ≤ 0.80. However, there was a wide variation between patients especially when testing concentrations ≤ 10 nM, and the antiproliferative responses were generally stronger for concanamycin A. Based on these results, the two drugs were tested at final concentrations of 10, 5 and 1 nM in the later experiments, all three corresponding DMSO concentrations were ≤0.01%, and DMSO 0.01% alone showed no effect on AML cells proliferation and viability (i.e., <10% difference from the corresponding DMSO-free control cultures). Finally, this concentration range of bafilomycin A1 may then reflect differences in cellular effects because in other cell types, effects on autophagosome/lysosome fusion are seen only at concentrations ≥ 10 nM [20,26]. Previous studies have also demonstrated that the patient heterogeneity of AML cell viability is also detectable when testing higher bafilomycin concentrations [28], and the concentration 10 nM is similar to [35] or close to [28] in terms of the concentration used in previous AML studies.

The chloroquine concentration was based on a dose–response experiment, and the selected concentration reflected the variation in antiproliferative effects between patients [33]. Cytarabine (Fresenius Kabi, Bad Homburg vor der Höhe, Germany) could be dissolved directly in culture medium.

### 2.3. Proliferation Assay

This assay has been described in detail previously [39]. Briefly, primary AML cells were seeded in flat-bottomed 96-well microtiter plates (VWR 734-2327; VWR^®^; Radnor, PA, USA) (50,000 cells/well; 200 μL medium/well). The culture medium was Stem Span SFEM™ supplemented with granulocyte–macrophage colony-stimulating factor (GM-CSF), Flt3 ligand (Flt3-L) and stem cell factor (SCF); all cytokines were purchased from PeproTech (Rocky Hill, NJ, USA) and used at a final concentration of 20 ng/mL. After six days of incubation at 37 °C in an atmosphere of 5% CO_2_, 37 kBq/well of ^3^H-thymidine (Perkin Elmer; Waltham, MA, USA) was added in 20 μL, and the cells were thereafter incubated for 18 h before they were harvested and nuclear radioactivity measured by liquid scintillation counting (presented as counts per minute, cpm). All experiments were performed in triplicates and the median cpm was used for all calculations.

Repeated negative controls showed a nuclear radioactivity corresponding to <180 cpm (controls with dead primary AML cells, and control wells only containing medium alone without cells). Significant proliferation was therefore defined as a nuclear radioactivity corresponding to >1000 cpm. The results are presented as the relative response (RR), i.e., the median cpm for drug-containing cultures relative to the cpm for the corresponding drug-free control cultures.

### 2.4. Analysis of AML Colony Formation

AML cells were first cultured in suspension cultures, i.e., 1 × 10^6^ AML cells seeded in 2 mL/well (24-well culture plates) of Stem Span SFEM medium supplemented with GM-CSF+Flt3-L+SCF, with or without concanamycin A 1 nM or bafilomycin A1 10 nM. The cells were cultured in 24-well culture plates (Nunc, Roskilde, Denmark) for one week before harvesting, centrifugation and resuspension in 0.5 mL RPMI medium (Sigma Aldrich, St. Louis, MO, USA) before 2.5 mL methylcellulose medium was added (MethoCult™ H4534 Classic medium without erythropoietin; StemCell Technologies, Vancouver, BC, Canada) to reach a final concentration corresponding to 0.33 × 10^6^ cells/mL of seeded cells from the initial suspension cultures. Cells were seeded in duplicates in 24-well plates (0.5 mL/well corresponding to 0.17 × 10^6^ originally seeded cells/well). After 14 days of additional in vitro incubation, the number of colonies with ≥20 cells (corresponding to at least 4 cell divisions) was estimated via light microscopy. The results are presented as the number of colonies per well, i.e., the number of detectable clonogenic cells per 0.16 × 10^6^ seeded cells at the initiation of suspension cultures.

### 2.5. Analysis of AML Cell Viability by Flow Cytometry

Primary AML cells were cultured in flat-bottomed 24-well culture plates (VWR 734-2325; VWR^®^) (1 × 10^6^ cells/mL, 1 mL/well) in Stem Span SFEM culture medium supplemented with GM-CSF, Flt3-L and SCF as described above. Cells were stained with propidium iodide (PI) and fluorescein isothiocyanate-conjugated Annexin V (Tau Technologie BV; Kattendijke, the Netherlands), and the percentages of viable Annexin V^−^PI^−^, early apoptotic Annexin V^+^PI^−^ and late apoptotic/necrotic Annexin V^+^PI^+^ cells were determined by flow cytometry, as described in detail previously [40].

### 2.6. Mutational Analyses

The molecular genetic analysis has been described in detail previously [38,41,42].

### 2.7. Analysis of Cytokine Levels

As described in detail previously [36], primary AML cells were cultured in flat-bottomed 24-well culture plates (VWR 734-2325; VWR^®^) (1 × 10^6^ cells/mL, 1 mL/well) in Stem Span SFEM medium supplemented with GM-CSF, Flt3-L and SCF. Supernatants were harvested after 48 h and stored at −80 °C until mediator levels were determined via Luminex analysis (R&D Systems; Minnesota, MN, USA).

### 2.8. Transwell Co-Cultures of Primary Human AML Cells and Mesenchymal Stem Cells

Normal human mesenchymal stem cells (MSC) (Lonza, Cambrex BioScience, Walkersville, MD, USA; healthy donor MSC24539, negative testing for mycoplasma, bacteria, and fungi) were expanded in complete mesenchymal stem cell growth medium (MSCGM^TM^; Lonza) containing 10% inactivated fetal bovine serum (FBS) and 4 mM L-glutamine. Expanded MSCs were trypsinated and later used in our experiments in passage four.

Co-cultures were prepared in transwell plates (Costar 3401 plates; Costar, Cambridge, MA, USA). MSC (2 × 10^4^ cells/well) were added to the lower chamber and 1 × 10^6^ AML cells to the upper chamber; the two cell populations were separated by a semipermeable membrane (0.4 μm pore size) and each transwell contained a total of 1.6 mL MSCGM™ medium [43]. For the proliferation assay, 275 kBq of ^3^H-thymidine (PerkinElmer, Waltham, MA, USA) was added in 150 μL medium to each well after 6 days of culture, and the cells were incubated for an additional 18 h before nuclear ^3^H-thymidine incorporation was measured. The MSCs did not reach confluence in any experiments during the seven days of culture.

### 2.9. Gene Expression Analyses

Our gene expression analyses of the V-ATPase interactome are based on global gene expression analyses that were performed as described in detail previously [41].

### 2.10. Proteomic and Phosphoproteomic Analyses

The analyses of AML cell proteomes and phosphoproteomes spiked with a super-stable isotope labeling with amino acids in cell culture (SILAC) mix were performed as described in detail previously [35,44,45].

### 2.11. Animal Studies

All experiments were approved by the Norwegian Animal Research Authority and performed in accordance with The European Convention for the Protection of Vertebrates Used for Scientific Purposes in an AAALAC accredited institution. The studies were approved by the Norwegian Food Safety Authority (FOTS), project number 28742, granted from 19082022–18082026). The animal models and the evaluation methodology have been described in detail previously [46]. Briefly, intravenous injection of MV4-11 or HL-60 AML cells (5 × 10^6^ cells/100 μL/mouse) was performed on female NOD/SCID IL2Rγnull (NSG) mice (Vivarium, University of Bergen; originally a generous gift of Dr. Leonard D. Shultz, The Jackson Laboratory). The AML cell lines were obtained from ATCC (USA) and cultured in accordance with the suppliers’ guidelines prior to engraftment. Both AML cell lines were routinely assessed for mycoplasma infection and were transduced using lentiviral particles to express red-shifted firefly luciferase and GFP as reporter genes (RediFect Red-FLuc-GFP, PerkinElmer Inc., Waltham, MA, USA). Animals harboring engrafted AML cells were randomized according to weight prior to being assigned to study groups.

For in vivo studies, Bafilomycin A1 (MedChemTronica, Stockholm, Sweden) was dissolved in DMSO (10%), PEG 400 (40%) tween (5%) and saline (45%) and sterile filtered. The daily intraperitoneal doses used in the experiments were 0.1, 1.0 and 2.0 mg/kg. Mice were regularly evaluated by weight and bioluminescence for the ANL cell experiments as described in detail in a previous publication [46]. The IVIS Spectrum (Perkin Elmer) was used for bioluminescence imaging (BLI). Ventral and dorsal BLI images of the mice were acquired weekly, 10 min after intraperitoneal (i.p.) administration of 150 mg/kg D-luciferin (Biosynth Carbosynth; Cat No. L-8220). All images were analyzed with Living Image Software (version 4.1). Gated region of interest was quantified with photon counts per second (p/sec). For the toxicity studies, the mice were evaluated by body weight and regular blood sampling for analysis of normal peripheral blood cell counts. Blood counts were measured from whole blood collected on EDTA, using an IDEXX Proceeded Hematology Analyzer (IDEXX Laboratories, Inc., Westbrook, ME, USA).

Animals inoculated with leukemic cells will suffer degrees of stress associated with disease progression and leukemia load. These animals were therefore scored daily and euthanized according to predefined and highly standardized criteria/endpoints including physical appearance/ruffled fur, behavior/activity, body movements (e.g., unstable walking, reduced mobility) and nutrition/weight loss.

### 2.12. Statistical and Bioinformatical Analyses

*Analysis of proteomic and phosphoproteomic data.* The Perseus 2.0.7.0 platform was used to clean, normalize and run two-sample unequal variance *t*-test with the proteomic and phosphoproteomic data [47]. The identification of differentially expressed proteins/phosphosites and the use of hierarchical clustering, protein–protein interaction (PPI analyses), sequence motif analyses and kinase–substrate enrichment have been described in detail in previous publications [35,44,45]. Gene ontology (GO) term enrichment and volcano plots were performed with Enrichr and VolcaNoseR web applications, respectively [48,49]. The Wilcoxon’s test for paired samples was used for statistical comparisons. The Mann–Whitney U test was used to compare different groups. Χ^2^ tests (Pearson’s χ^2^ test and the likelihood ratio) were used to investigate correlations for continuous data. Fisher’s exact test was used for analysis of categorized data. Differences were regarded as statistically significant when *p* < 0.05.

*Additional statistical and bioinformatical analyses.* Unsupervised hierarchical clustering analysis was conducted in J-Express (Molmine, Bergen, Norway). Statistical differences in averages between animal treatment groups were determined using a two-tailed Student *t* test via Pearson correlation (GraphPad Prism^®^ 7.0, GraphPad Software, La Jolla, CA, USA). A one-way analysis of variance (ANOVA) was performed to ensure no statistically significant difference in weights between the animals in the treatment groups.

## 3. Results

### 3.1. AML Cell Proliferation: V-ATPase Inhibition Decreases Cytokine-Dependent Proliferation of Primary Human AML Cells, but the Susceptibility to V-ATPase Inhibition Shows a Wide Variation between Patients

**Figure 1:**
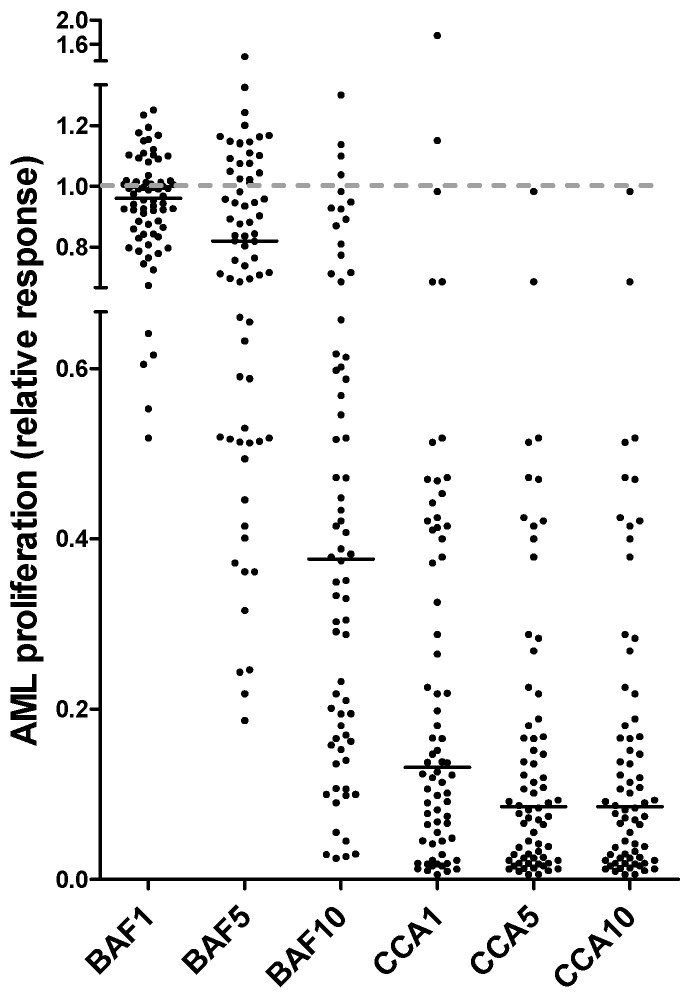
The effect of the V-ATPase inhibitors bafilomycin A1 and concanamycin A (both tested at 10/5/1 nM) on cytokine-dependent proliferation of primary human AML cells. Leukemic cells derived from 80 patients were examined in the ^3^H-thymidine incorporation assay; the figure presents the results for the 70 patients showing detectable proliferation (nuclear radioactivity > 1000 cpm) in the drug-free controls (median response 13,683 cpm, range 1317–173,197 cpm). ^3^H-thymidine was added after 6 days of culture and nuclear radioactivity determined 18 h later. The results are presented as the relative proliferative response, i.e., cpm in drug-containing cultures relative to the radioactivity for corresponding drug-free controls. Each dot presents the results for one patient; no effect of the agent (i.e., corresponding to a relative response of 1.0) is indicated by the dotted line in the figure.

Detectable cytokine-dependent proliferation in the ^3^H-thymidine incorporation assay was defined as nuclear radioactivity corresponding to at least 1000 cpm [39]. Seventy of the 80 patients showed detectable proliferation in drug-free control cultures. We investigated the effects of the two V-ATPase inhibitors, bafilomycin A1 and concanamycin A, on cytokine-dependent proliferation. Based on initial dose–response experiments (see Section 2.2) both drugs were tested at the three concentrations 10, 5 and 1 nM. The overall results are summarized in Table 2 and presented in more detail as the relative responses of individual patients in Figure 1. The median proliferation in the drug-free control cultures corresponded to 11,683 cpm (range 1317–173,197 cpm). When analyzing the overall results, both drugs showed statistically significant antiproliferative effects for all concentrations examined (Table 2; *p* ≤ 0.001). However, the antiproliferative effect showed a wide variation between patients, and this variation was observed especially for bafilomycin, whereas concanamycin showed generally stronger effects for all three concentrations examined. Thus, even though the two inhibitors are regarded as structurally related [20,21,26], they differ with regard to the strength of their antiproliferative effect.

### 3.2. AML Cell Proliferation: Patients Showing a Strong Antiproliferative Effect of V-ATPase Inhibition Are Heterogeneous, but a Strong Effect Is Seen Especially for Patients with Secondary/Relapsed AML

There was a wide variation between patients with regard to the antiproliferative effect of V-ATPase inhibition when this effect was estimated as the relative responses (Table 2, Figure 1). The patient heterogeneity was most clearly observed when testing bafilomycin A1 10 nM; a relatively wide variation could also be seen for bafilomycin A1 5 nM, whereas concanamycin A had a generally stronger antiproliferative effect than bafilomycin when comparing equimolar concentrations (Figure 1). Our analysis of patient heterogeneity with regard to the antiproliferative effect of V-ATPase inhibition was therefore based on the relative responses when testing bafilomycin A1 10 nM, i.e., the proliferation (cpm) for cytokine-supplemented cultures containing bafilomycin A1 10 nM relative to the proliferation (cpm) for the corresponding bafilomycin A1-free cytokine-supplemented culture.

Based on the relative antiproliferative effect of bafilomycin A1 10 nM (Figure 1), we classified the patients as strong (relative responses ≤ 0.30 of the corresponding control cultures), intermediate (relative response 0.31–0.60), and weak/non-responders (relative response > 0.60). According to these definitions, 37 patients who showed detectable AML cell proliferation in control cultures were classified as strong responders to V-ATPase inhibition, and we compared the clinical and biological characteristics of these patients with the 33 intermediate/weak/non responders:

- Secondary AML includes patients with antecedent myeloid malignancies or previous exposure to cytotoxic treatment, and clinical studies have shown that these patients have reduced AML-free survival after intensive therapy [50,51,52,53,54,55,56,57,58,59] due to chemoresistance and increased relapse risk [2,60]. The strong responders showed a significantly increased frequency of patients with secondary/relapsed AML (17 out of 37 patients, see Table S2) compared with the intermediate/weak responders (6 out of 33 patients; Fisher’s exact test, *p* = 0.0211). A significant difference was also seen when only the secondary AML patients but not the patients with AML relapse were compared in the statistical analysis (*p* = 0.0327).
- The strong responders showed a wide variation with regard to genetic abnormalities; 15 of them showed a normal karyotype, whereas the other patients had both adverse (e.g., 5 with complex chromosomal abnormalities) and favorable (1 patient with normal karyotype, no Flt3 abnormality but NPM1 insertion) genotypes [1,2,60]. The frequencies of Flt3 and NPM1 mutations did not differ either (Table 2).
- Targeted next-generation sequencing were available for 33 consecutive patients; a total of 57 mutations were analyzed, and 32 of them were detected for at least 1 of the patients (Table S3). These analyses further illustrate the genetic heterogeneity of strong responders to V-ATPase inhibition. Furthermore, the antiproliferative effect of bafilomycin A1 10 nM did not differ between patients with and without MDS-related mutations (i.e., ASXL1, BCOR, EZH2, RUNX1, SF3B1, SRSF2, STAG2, U2AF1 orZRSR2) (Table S3) [2,57]. Finally, MDS-related mutations were detected for 17 patients (Table S4), but the antiproliferative effect of these 17 patients did not differ significantly from the effect for patients without MDS-associated mutations (Fisher’s exact test, *p* = 0.0899).
- A previous study showed that AML patients could be subclassified based on the constitutive activation of the PI3K-Akt-mTor pathway in the AML cells [61], and there is also crosstalk between this pathway and V-ATPase [62,63]. A consecutive subset of 45 patients was classified as showing either high or low general constitutive PI3K-Akt-mTOR activation in their AML cells [61]. The strong responders included 11 patients with high and 12 patients with low constitutive pathway activation, and this was not different from the other patients showing a weak antiproliferative effect of bafilomycin A1 (Table S2).
- The two groups (37 strong responders versus 33 intermediate/weak/no responders) did not differ with regard to age, sex or AML blast differentiation (FAB classification, CD34 expression) (Table 2). The cytokine-dependent proliferation in drug-free control cultures showed a wide variation both for patients showing strong (median cytokine-dependent proliferation 10,901 cpm, range 1459–173,197 cpm) and intermediate/weak/no (median 11,890 cpm, range 1317–78,345 cpm) antiproliferative effects of bafilomycin A1 10 nM and did not differ significantly between these two patient subsets (Table S2).
- Chloroquine is regarded as an inhibitor of autophagy [28,64,65,66], and a consecutive subset of 39 of the present 70 patients were also cultured with and without chloroquine 2.5 μM in our ^3^H-thymidine incorporation assay. The relative antiproliferative effect of chloroquine was classified as weak or strong (>0.30 versus ≤0.30) based on the same criteria as used for bafilomycin A1 (see above); the majority of strong responders to chloroquine were also strong responders to bafilomycin 10 nM (15 out of 20), and the majority of weak responders to chloroquine were also classified as weak responders to bafilomycin A1 10 nm (14 out of 18; Fishers exact test, *p* = 0.0029), thus indicating that there is an association between the antiproliferative effects of chloroquine and bafilomycin A1.

To conclude, the antiproliferative effect of V-ATPase inhibition showed no association with the characterized cytogenetic or molecular genetic abnormalities, but there was an association between a strong antiproliferative effect and secondary AML.

### 3.3. AML Cell Proliferation: Effects on AML Cell Proliferation of Combining V-ATPase Inhibition with Low-Dose Cytarabine, the Two Drugs Show Additive Antiproliferative Effects

We investigated the effect of combining concanamycin A 1 nM with cytarabine 1 μM on primary AML cell proliferation. All 70 patients showing detectable cytokine-dependent proliferation were included in these experiments. The antiproliferative effect of concanamycin A showed a wide variation between patients when comparing the relative responses (Figure 1); for 48 of the 70 patients, the nuclear radioactivity/proliferation in the concanamycin-containing cultures corresponded to <1000 cpm (i.e., defined as undetectable levels; it was not possible to demonstrate an additional antiproliferative effect by cytarabine), whereas for the other 22, patients the proliferation in both concanamycin and cytarabine cultures reached detectable levels. The overall results are summarized in Table S11; and the following observations were made via a more detailed analysis:

- For the 48 patients with undetectable proliferation in concanamycin-containing cultures, the proliferation in cytarabine-containing cultures reached detectable levels for all patients and corresponded to a median relative response of 0.62 (range 0.06–1.26). A similar low/undetectable proliferation as for the concanamycin-containing cultures was also seen for all the corresponding cultures supplemented with concanamycin + cytarabine.
- The overall results for the 22 patients with detectable proliferation in the concanamycin-containing and cytarabine and concanamycin + cytarabine cultures are presented in Table 3. Concanamycin A 1 nM had a generally stronger antiproliferative effect than cytarabine 1 μM for these patients. The antiproliferative effect of combining concanamycin A and cytarabine was significantly stronger than the effect of each drug tested alone (Table 3). Thus, concanamycin A and cytarabine show additive antiproliferative effects, as was observed for all these 22 patients.

Thus, even when using the stronger V-ATPase inhibitor, concanamycin A with low-dose cytarabine seems to have additive antiproliferative effects compared with the V-ATPase inhibition alone, at least for patients with relatively weak antiproliferative effects of concanamycin alone, and we found no evidence for counteracting effects between the two drugs.

### 3.4. AML Cell Proliferation: Possible Protein Biomarkers for Susceptibility to V-ATPase Inhibition Identified by Proteomic and Phosphoproteomic Comparisons of Primary AML Cells Showing Weak versus Strong Antiproliferative Effects of V-ATPase Inhibition

Proteomic and phosphoproteomic profiles were available for a consecutive subset of 15 younger AML patients (below 60 years of age), and we compared two contrasting patient groups with an antiproliferative effect corresponding to a relative response (i.e., the proliferation in the presence of bafilomycin A 10 nM being) <0.30 versus >0.60, respectively. These comparisons therefore included eight strong responders (Tables S1 and S2; patients 5, 15, 55, 59, 64, 69, 71) and seven weak responders (Table S1, patients 2, 23, 31, 34, 36, 41, 51, 73).

**Figure 2:**
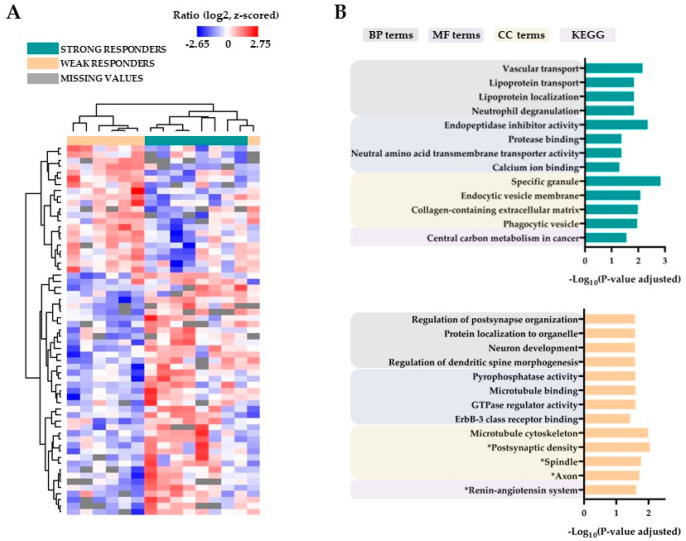
A comparison of the global primary AML cell proteome for leukemic cells showing strong antiproliferative effects of bafilomycin A1 10 nM (eight patients, relative responses ≤ 0.30) and weak/no antiproliferative effects of bafilomycin (seven patients, relative responses ≥ 0.60). (**A**) Unsupervised hierarchical clustering analysis that included all 15 patients and, based on the expression of 61 differentially expressed proteins, identified two main patient clusters. The clustering of the differentially expressed proteins is presented in the left part of the figure, and the proteins are listed from the top of the clustering analysis and downwards in Table S6. (**B**) Gene ontology (GO) analyses of differentially expressed proteins showing significantly increased levels in AML cells characterized by strong antiproliferative effects of bafilomycin A1 10 nM (upper part) and cells characterized by weak/no antiproliferative effects (lower part). These analyses were based on identification of biological processes (BP), molecular functions (MF), cell compartment (CC) and KEGG classification, as indicated at the top of the figure (* Significant GO terms and KEGG pathway with unadjusted *p*-value).

The 61 differentially expressed proteins are listed in Table S5. The results from hierarchical clustering and GO term analyses are presented in Figure 2 (see also Table S6), the results from the volcano plot and protein interaction analyses are presented in Figure S1, and the differentially expressed proteins also identified in the volcano plot/PPI analyses are described in detail in Table S5. First, the clustering analysis based on all differentially expressed proteins showed that the eight strong responders clustered together in the same main cluster; six of the seven weak responders also formed a separate main cluster but with the last weak responder clustering alone (Figure 2A). Second, the GO term analyses showed that the differentially expressed proteins in strong and weak responders are involved especially in intracellular organellar trafficking and cytoskeletal functions, respectively (Figure 2B). Third, PPI analyses identified strong interactions between annexin A3 (ANXA3)-annexin A11 (ANXA11) and cyclin-dependent kinase 5 (CDK5)-prelamin-A/C (LMNA) in strong and weak responders, respectively (Figure S1; Tables S5–S7 first part). Several of these differentially expressed proteins were also identified in the volcano plot analysis (Tables S5 and S7, Figure S1). Finally, four of the identified proteins have been associated with prognosis or chemoresistance in previous AML studies (Table S7 first part): (i) transferrin receptor/iron metabolism is associated with prognosis in human AML; (ii) experimental studies suggest that fibronectin/integrin interactions are involved in AML chemoresistance; and (iii) both chitinase 3-like-1 (CHI3L1) and ANXA3 are associated with prognosis in human AML.

Thus, weak and strong responders did not show differential expression of proteins in the V-ATPase interactome, but they differed in the functional/molecular context of V-ATPase (i.e., regulation of organellar functions), as well as in previously identified potential prognostic AML markers.

**Figure 3:**
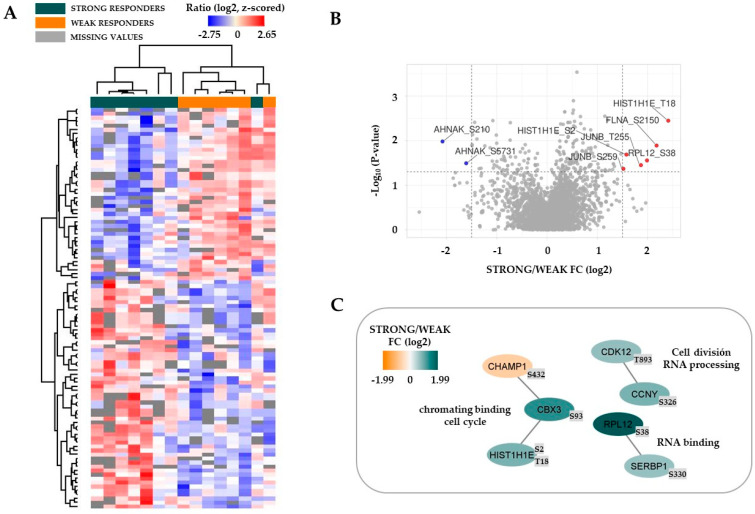
A comparison of the global primary AML cell phosphoproteome for leukemic cells characterized by either a strong antiproliferative effect of bafilomycin A1 10 nM (eight patients, relative response ≤ 0.30) or a weak effect (seven patients, ≥0.60). (**A**) Unsupervised hierarchical clustering analysis including all 15 patients and based on all 100 differentially expressed phosphorylation sites identified two main patient clusters. The proteins are listed from the top of the heatmap and downwards in Table S9. (**B**) The figure presents the results from a volcano plot analysis based on all differentially expressed phosphorylation sites, and the indicated points above the non-axial horizontal grey line represent protein sites with significantly different phosphorylation (*p* < 0.05). (**C**) Protein–protein interaction (PPI) analyses of differentially phosphorylated proteins.

We thereafter compared the protein phosphorylation profiles for the same two contrasting patient groups, and 100 differentially regulated phosphorylation sites were identified. Fifty-seven phosphosites were increased for patients with a strong antiproliferative effect of bafilomycin A1 10 nM, whereas fourty-three phosphosites were increased for the low responders (Table S8). First, an unsupervised hierarchical clustering analysis based on all 100 phosphosites identified two main patient subsets, and all except one strong responder constituted the left main patient cluster (Figure 3A; for the phosphosite listing see Table S9). Second, several of the differentially expressed proteins were also identified in the volcano plot and PPI analyses (Figure 3B,C, Table S7 second part, Table S8). Finally, several of the identified proteins are important in AML: the JunB proto-oncogene (JUNB) and cyclin dependent kinase 12 (CDK12) are both regulators of AML stem cells, whereas filamin A (FLNA) is a fusion partner in AML-associated translocations (see Tables S7 and S8 for details).

**Figure 4:**
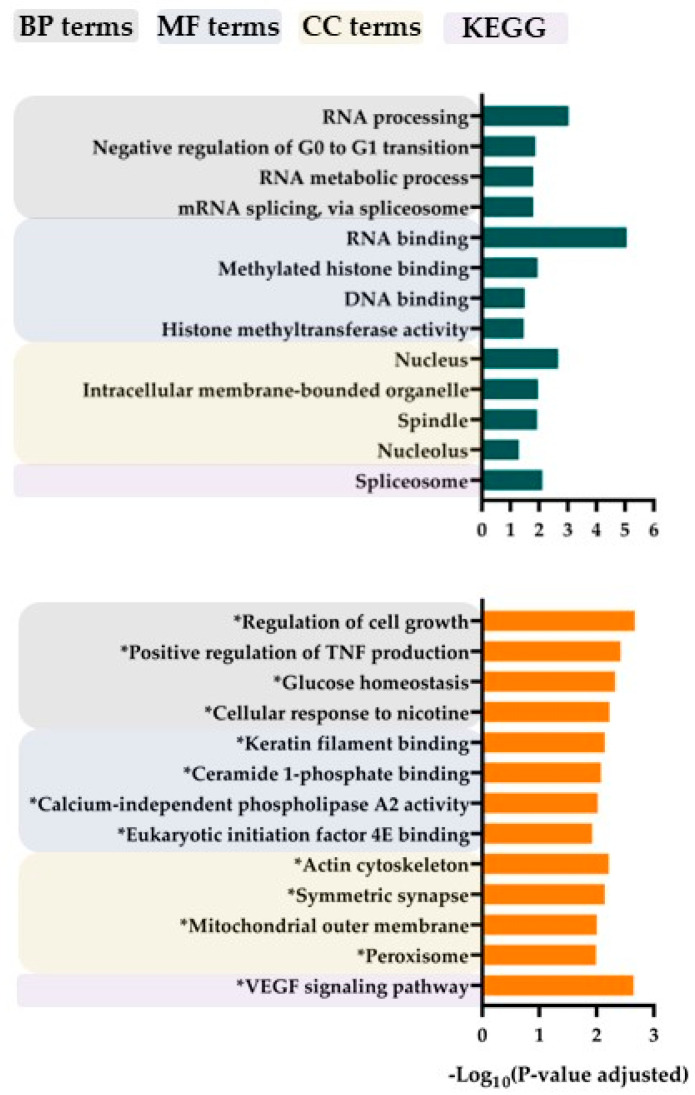
A comparison of the global primary AML cell phosphoproteome for leukemic cells characterized by either a strong antiproliferative effect of bafilomycin A1 10 nM (eight patients, relative response ≤ 0.30) or a weak effect (seven patients, ≥0.60). GO analyses of differentially expressed phosphorylation sites showing significantly increased levels for AML cells/patients showing strong antiproliferative effects of bafilomycin are presented on (**upper part**, dark green color), the results for AML patients/cells showing weak effects are presented on the (**lower part,** orange color). These analyses were based on biological processes (BP), molecular functions (MF), cell compartment (CC) and KEGG classification, as indicated at the (**top**) of the figure (* indicates that the term reached statistical significance according to an uncorrected *p*-value).

**Figure 5:**
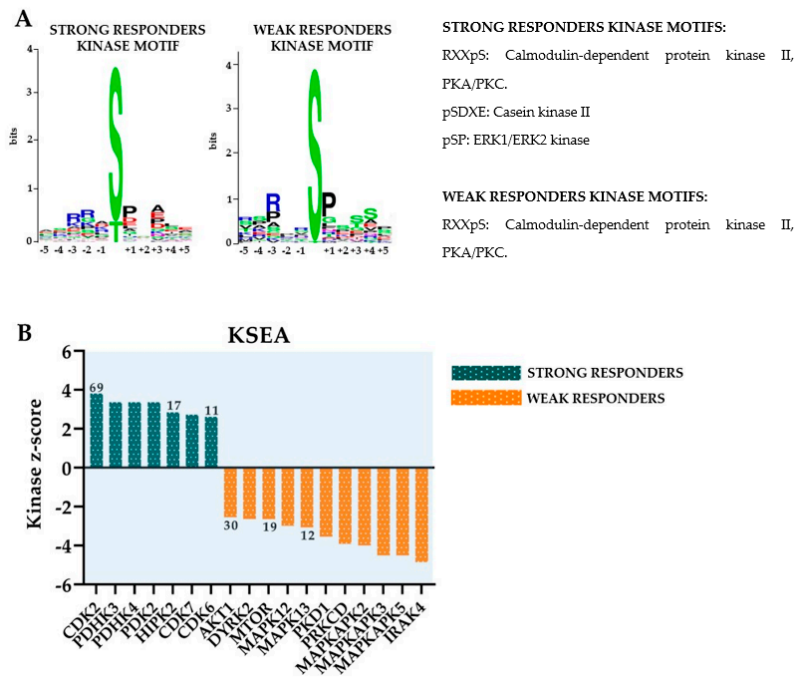
A comparison of the global primary AML cell phosphoproteome for AML cells characterized by either a strong antiproliferative effect of bafilomycin A1 10 nM (eight patients, relative response ≤ 0.30) or a weak effect (seven patients, relative responses ≥ 0.60); the results from sequence motif analyses and kinase substrate enrichment analyses (KSEA). (**A**) The figure presents the results from sequence motif analyses (the letters in the kinase motifs indicate amino acid identity, size of the letter indicates the relative strength of the association) for AML cells/patients showing strong (**left part**) and weak (**right part**) antiproliferative effects of bafilomycin A1. The most significant kinase motifs are indicated to the right in the figure. (**B**) KSEA of differentially regulated phosphorylation sites. The kinase z-score (*y* axis) is the normalized score for each kinase (*x* axis), weighted by the number of identified substrates. Numbers close to the bars indicated the number of substrates; this is only shown for kinases with at least 10 substrates.

GO term analyses of differentially expressed phosphorylation sites showed that strong responders to bafilomycin A1 were characterized by increased phosphorylation, especially for proteins involved in transcriptional regulation/RNA functions, whereas the weak responders showed increased levels of phosphorylation of proteins involved in the regulation of proliferation/cytoskeleton/signal transduction/metabolism (Figure 4). Furthermore, the differences in protein phosphorylation are also characterized by analysis of the sequence motifs and seem to be caused by effects of various protein kinases (Figure 5). Although calmodulin-dependent protein kinase II (CAMK2), cAMP-dependent protein kinase (PKA), protein kinase C delta type (PKCD) and mitogen-activated protein kinases 3/1 (ERK1/2) appear to be active in both strong and weak responders groups, casein kinase 2 activity seems to be increased only in the strong responders group.

### 3.5. AML Cell Proliferation: V-ATPase Inhibition Has an Antiproliferative Effect on the More Immature Clonogenic AML Cell Subset

**Figure 6:**
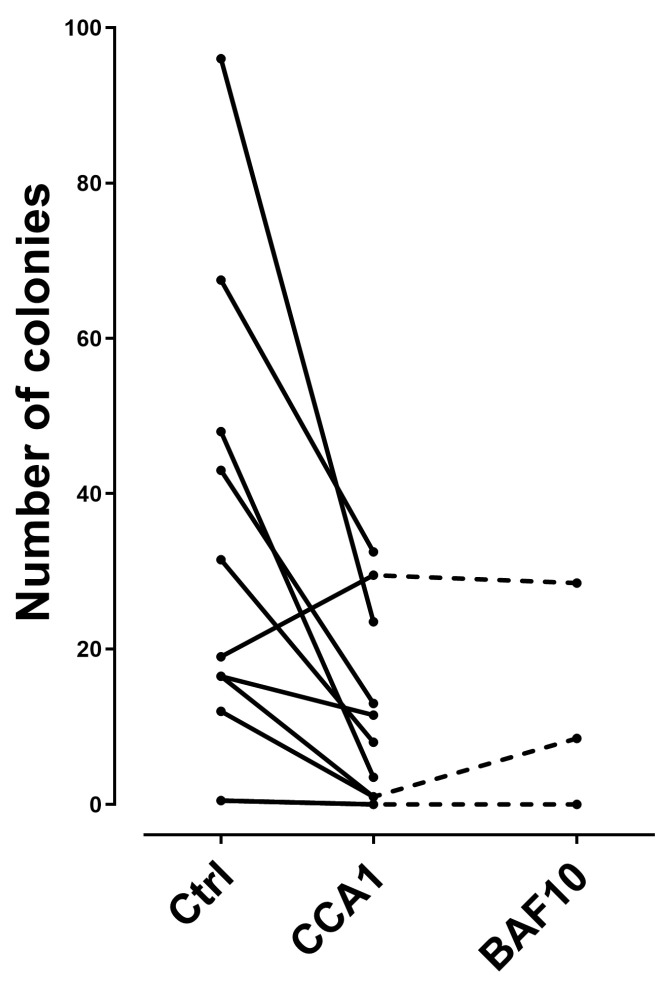
The effects of V-ATPase inhibitors on clonogenic AML cell proliferation. Primary human AML cells derived from 10 patients were precultured in suspension cultures prepared either in medium alone or medium with V-ATPase inhibitors in suspension cultures for 7 days before the cells were washed and transferred to the colony formation assay; the medium used in this last assay did not contain V-ATPase inhibitors. The number of clonogenic cells was determined for control suspension cultures prepared with medium alone and for suspension cultures prepared with either concanamycin A 1 nM (10 patients) or bafilomycin 10 nM (3 patients). All colony formation cultures were prepared in duplicates, and the results are presented as the mean number of colonies containing at least 20 cells; i.e., a number corresponds to at least four cell divisions. The results are presented as a comparison of the number of clonogenic cells per 0.16 × 10^6^ seeded AML cells in the initial suspension cultures.

We investigated the effect of V-ATPase inhibitors on the clonogenic proliferation of primary human AML cells derived from 10 unselected patients that show an antiproliferative effect of concanamycin A I nM corresponding to a relative response < 0.30 in the suspension culture assay. The overall results are presented in Figure 6. When analyzing the overall results, preincubation of primary AML cells reduced the number of colonies, i.e., they caused a significant inhibition of clonogenic AML cell proliferation (Wilcoxon’s test for paired samples, *p* = 0.012). The number of colonies was reduced by concanamycin A for all except one patient. Finally, the effect of bafilomycin A 10 nM was similar to the concanamycin A effect for the three patients examined. Thus, V-ATPase inhibition has an antiproliferative effect also when analyzing the clonogenic AML cell subset.

### 3.6. The V-ATPase Interactome: Primay AML Cells Are Heterogeneous with Regard to the mRNA Expression of Various Components of the V-ATPase Interactome, and Secondary AML Is Associated with a Specific mRNA Profile

**Figure 7:**
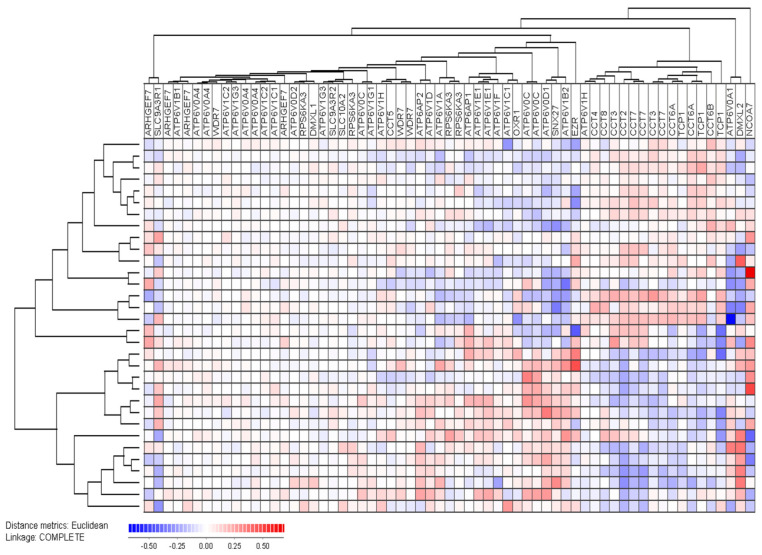
The mRNA expression of molecules included in the V-ATPase interactome: an unsupervised hierarchical clustering analysis based on the mRNA levels of 63 genes belonging to the V-ATPase interactome in primary human AML cells derived from 32 unselected/consecutive patients. The gene identity is indicated at the top of the figure, and the identification of two main patient clusters can be seen from the clustering to the left in the figure. The identity and characteristics of the patients included in these two main patient clusters are presented in detail in Table S10.

Analyses of the global gene expression profiles were available for 32 consecutive patients, and we did an unsupervised hierarchical clustering analysis based on identified members of the V-ATPase interactome (Figure 7) [3,67,68]. The main molecular classes of the interactome include 19 transporters (18 ATP molecules plus SLC10A), 9 chaperonin-containing TCP (CCT) complex proteins, 6 trafficking proteins and 5 V-ATPase specific accessory proteins. The majority of ATP transporters clustered close to each other in the left main interactome cluster, whereas most CCT molecules clustered together in the right main cluster (Figure 7).

Our clustering analysis identified two main patient subsets including 18 (upper) and 14 patients (lower), respectively. The upper main cluster was characterized by relatively high levels of CCT expression, whereas the lower main cluster showed relatively low CCT expression but generally high expression of ATP molecules/genes. The clinical and biological characteristics of the patients in each of the two main patient clusters are presented in Table S8. The lower subset included a significantly increased number of patients with secondary AML (1/18 in upper versus 9/14 in the lower cluster; Fisher’s exact test *p* = 0.0016). Furthermore, the frequency of patients with Flt3-ITD did not differ between the two main clusters, but the majority of seven out of the 10 Flt3-ITD positive patients clustered close to each other in the upper main cluster (i.e., they showed similarities in their V-ATPase interactome expression).

### 3.7. Apoptosis Regulation: V-ATPase Inhibition Decreases the Viability of Primary Human AML Cells, but These Proapoptotic Effects Show a Wide Variation between Patients

We used cryopreserved primary AML cells in all our present experiments. Previous studies have demonstrated that cryopreserved AML cells usually include at least 70% viable cells (Annexin V^−^PI^−^) together with minor subsets of early apoptotic (Annexin^+^PI^−^) and late apoptotic/necrotic cells (Annexin V^+^PI^+^) immediately after thawing [40]. During the first days of in vitro culture, cryopreserved AML cells undergo spontaneous or stress-induced in vitro apoptosis mainly due to chaperon-induced apoptosis [40,69]. There is a wide variation between patients with regard to the degree of spontaneous apoptosis during culture. We investigated the fraction of viable, early apoptotic and late apoptotic/necrotic cells after 48 h of in vitro culture in medium with and without bafilomycin A1 and concanamycin A, and the viability in drug-containing cultures is then determined by a combined effect of spontaneous and drug-induced apoptosis.

**Figure 8:**
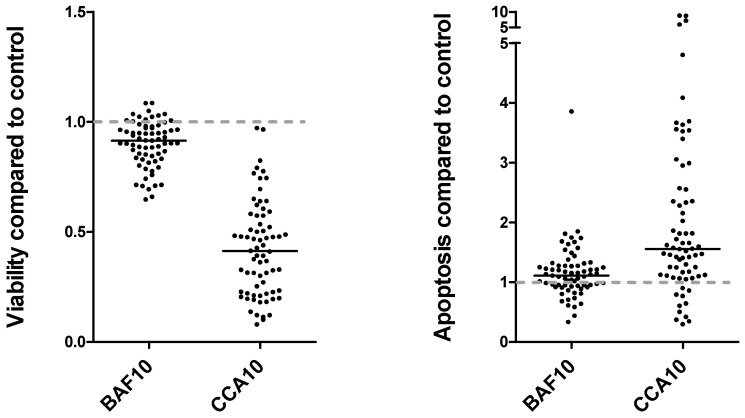
The effects of V-ATPase inhibitors on primary AML cell viability. Primary AML cells derived from all the 80 patients were incubated in medium alone or medium with bafilomycin A1 10 nM or concanamycin A 10 nM. The AML cell viability was investigated after 48 h of incubation by using our Annexin V/PI flow cytometric assay. The (**left**) part of the figure shows the relative number of viable cells, i.e., percentage of viable Annexin-V^−^PI^−^ cells in drug-containing cultures relative to the drug-free controls. The (**right**) part of the figure shows the relative number of early apoptotic Annexin-V^+^PI^−^ cells in the same cultures. No effect of the agents is indicated by the grey dotted line (i.e., corresponding to a relative response of 1.0).

Primary human AML cells from all 80 patients were also incubated with bafilomycin A1 10 nM and concanamycin A 10 nM for 48 h before viability was examined by flow cytometry. The overall results when comparing the relative viability (i.e., viability in drug-containing versus drug-free controls) are presented in Figure 8 (left) and Table S10. Even though both drugs decreased the viability significantly (*p* < 0.005), the effect was generally weaker for bafilomycin A1 (i.e., less variation between patients) than for concanamycin A, which showed a wider variation with a strong viability-decreasing effect for several patients. The relative viability in concanamycin A-containing cultures showed no significant association with the degree of spontaneous apoptosis in drug-free control cultures.

To investigate the variation in spontaneous in vitro apoptosis between our 80 patients, we analyzed the results for concanamycin A 10 nM in more detail. Sixty of the eighty patients showed more than 30% viable cells (variation range 97.9–31.5%), whereas only twenty of the patients showed a viability below 30% (range 28.6–2.6%) after 48 h of culture in medium alone. This variation of viability in control cultures did not show any significant associations with secondary versus de novo AML, differentiation (FAB classification, CD34 expression), genetic abnormalities (karyotype, NPM1 mutations, Flt3-ITD) or cytokine-dependent proliferation.

We also compared the percentage of early apoptotic (i.e., Annexin V^+^, propidium-iodide^−^) cells for drug-containing and control cultures. The percentage of early apoptotic cells was significantly increased especially for concanamycin A-containing cultures compared with the corresponding drug-free controls (Figure 8 right, Table S11, *p* < 0.005), suggesting that the decreased viability associated with V-ATPase inhibition was caused by increased apoptosis. Finally, as expected, the percentage of late apoptotic/necrotic cells was significantly increased for the drug-containing cultures compared with the drug-free controls (Table S11, *p* < 0.0005 for both drugs). The effects on apoptotic/necrotic cells did not show any significant associations with differentiation (morphology, CD34 expression), genetic abnormalities (karyotype, NPM1 mutations, Flt3-ITD) or antiproliferative effects. Finally, the percentage of viable cells in bafilomycin A1 and concanamycin A cultures showed significant correlation with the viability in drug-free control cultures (Table 4), and even though cytarabine 10 nM had an antiproliferative effects, its effect on cell viability was very weak (Table 4).

### 3.8. Extracellular Mediator Release: V-ATPase Inhibition Increases Constitutive Release of Several Soluble Mediators by Primary AML Cells, but This Effect Is Relatively Weak and the Wide Variation between Patients in Constitutive Release Is Maintained in the Presence of V-ATPase Inhibitors

We investigated the release of 19 soluble mediators by primary human AML cells derived from the 80 patients; the cells were then cultured under standardized in vitro conditions for 48 h before the supernatants were harvested. All mediators showed an expected wide variation between patients in the control cultures [36], and this wide variation was also maintained in the presence of bafilomycin A1 10 nM and concanamycin A 10 nM (Table S12). It can be seen that the two V-ATPase mediators showed similar effects and increased the absolute levels for several of the soluble mediators, especially for chemokines and interleukins. The effect on the levels of proteases/protease inhibitors/growth factors varied between the two agents.

We also did unsupervised hierarchical clustering analyses based on the absolute soluble mediator levels in medium control cultures (Figure S3) and the levels in bafilomycin A1- and concanamycin 1-containing cultures (Figure S4). The patient clustering showed a similar pattern in all three clustering analyses. The patients formed two main clusters in all three analyses, and the lower main clusters included a minority of patients with generally low mediator levels. On the other hand, the upper main patient clusters could be further divided into two subclusters: one subcluster included patients with generally high mediator levels, whereas the other subcluster included patients with intermediate levels. It can be seen that most patients classified as low/intermediate/high releasers based on the medium control analysis (Figure S3) clustered together or close to one another also in the presence of V-ATPase inhibitors; i.e., all the originally classified high and low releaser patients from the medium control cultures clustered in different main clusters also in the presence of the two V-ATPase inhibitors (Figure S3). These results show that not only the wide variation ranges but also the original (i.e., medium control culture) differences between patients in their overall soluble mediator release profiles are largely maintained when V-ATPase inhibitors are present during culture.

**Figure 9:**
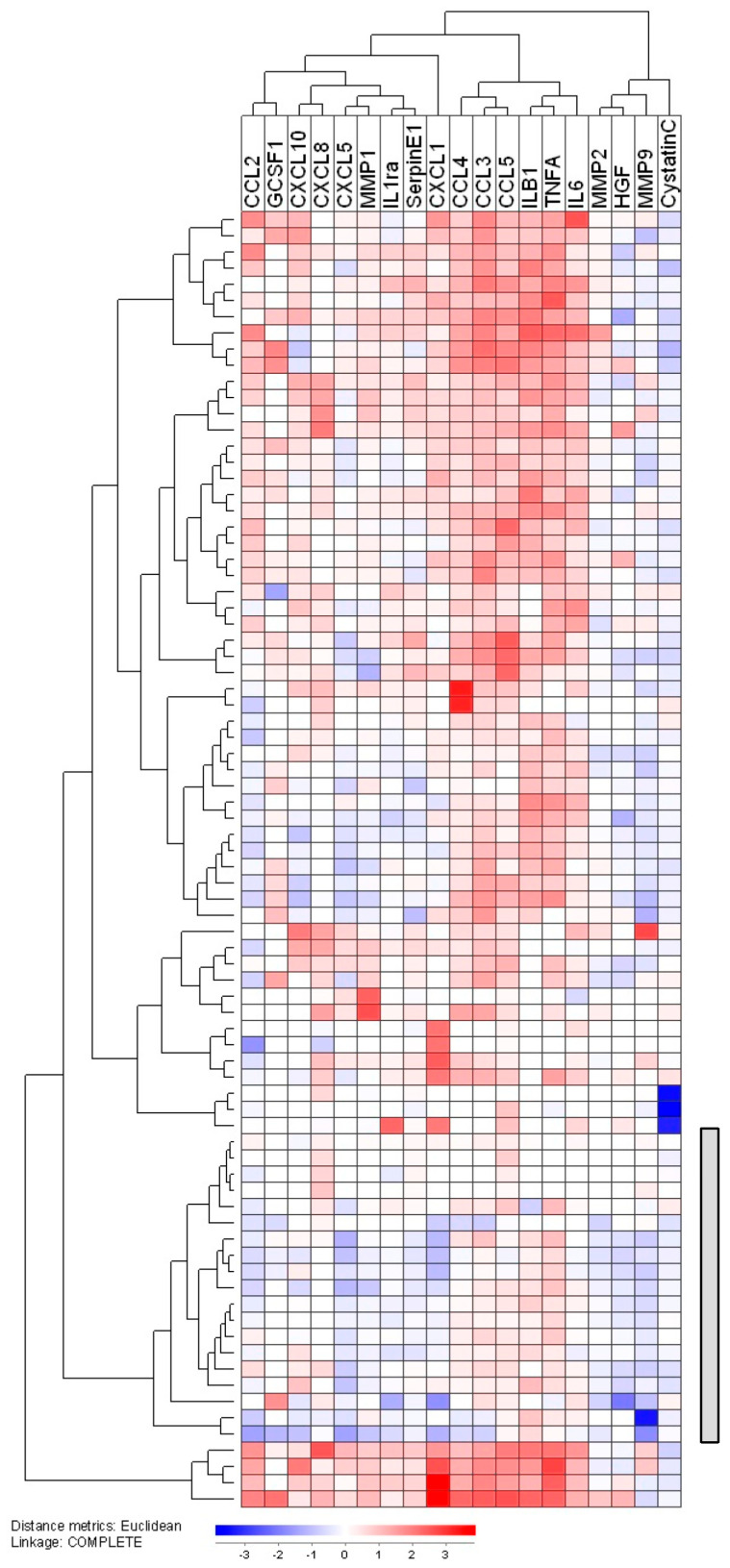
Effects of concanamycin A on the constitutive cytokine release by primary human AML cells. Leukemic cells from 80 patients were cultured for 48 h in cytokine-supplemented growth medium with and without concanamycin A 10 nM before mediator levels were determined in the culture supernatants. The relative mediator level was determined for each patient and cytokine; i.e., the mediator level for the drug-containing culture relative to the same mediator level in the corresponding medium control cultures. An unsupervised hierarchical clustering analysis was performed based on these relative mediator levels; the white color indicates that the level in control and concanamycin A cultures did not differ. A subset/subcluster of 19 patients with weak/no effect of concanamycin A on the cytokine levels formed a separate subcluster (indicated by the grey column to the right), and 11 of these 19 patients were also included among 23 patients that formed a separate low-effect cluster when testing bafilomycin A1 (Figure S12).

Finally, we estimated the mediator ratio for all the mediators and all patients investigated, i.e., the mediator level in inhibitor-containing cultures relative to the corresponding level in drug-free controls. This was conducted both for bafilomycin A1 and concanamycin A, and we then conducted unsupervised hierarchical clustering analyses based on the overall relative responses for bafilomycin A1 (Figure S5) and concanamycin A (Figure 9). In both these analyses, we identified patient subsets that showed no or only minor effects of V-ATPase inhibitors; this was seen for a subset of 23 patients when bafilomycin A1 was present and for 19 patients in the presence of concanamycin A. However, these patients characterized by no/minor effects of V-ATPase inhibition did not differ significantly with regard to their clinical and biological characteristics (Tables S13 and S14), and they did not differ from the other patients with regard to the antiproliferative effect of bafilomycin A1/concanamycin A.

### 3.9. Extracellular Mediator Release: Effects of Combining Low-Dose Cytarabine and V-ATPase Inhibition on Constitutive Mediator Release; V-ATPase Inhibitions Modulates the Constitutive Release also in the Presence of Cytoarabine, but the Wide Variation between Patients Is Maintained also in the Presence of Combined Therapy

We investigated the effect of cytarabine 10 nM on the constitutive soluble mediator release by primary AML cells derived from all 80 patients. These experiments showed that the wide variation in soluble mediator release (19 mediators examined) was maintained also in the presence of cytarabine, but when comparing the overall results, cytarabine significantly increased the levels of TNFα, whereas the levels of SerpinE1, MMP2, MMP1, IL1RA, CCL3 and CCL4 were significantly decreased (Figure S6). However, it should be emphasized that the effects of cytarabine were relatively small compared with the wide variations in soluble mediator levels between patients.

We then investigated the effect of concanamycin A 1 nM on the constitutive AML cell release of soluble mediators in the presence of cytarabine 1 nM. All 80 patients were included in these studies. A wide variation in the constitutive release of 10 soluble mediators were seen both when cytarabine was tested alone and in combination with concanamycin. Concanamycin A altered the soluble mediator profile of primary AML cells also in the presence of cytarabine (Figure S7); when comparing the overall results, significantly decreased levels were seen for HGF, cystatin C and CXCL8, whereas the levels of GCSF1, MMP1, TNFα, IL6, IL1RA, IL1β, CXCL10, CXCL8, CXCL1, CCCL5, CCL4 and CCL3 were significantly increased. Thus, concanamycin A can modulate the constitutive soluble mediator release profile of primary human AML cells even in the presence of cytarabine; most mediators then show increased levels, and this is similar to the effect of concanamycin A observed for AML cells cultured in medium alone without cytarabine (Figure S2).

### 3.10. AML Cell Communication: The Antiproliferative Effect of V-ATPase Inhibitors on Primary AML Cells Is Maintained in the Presence of Leukemia-Supporting MSC

**Figure 10:**
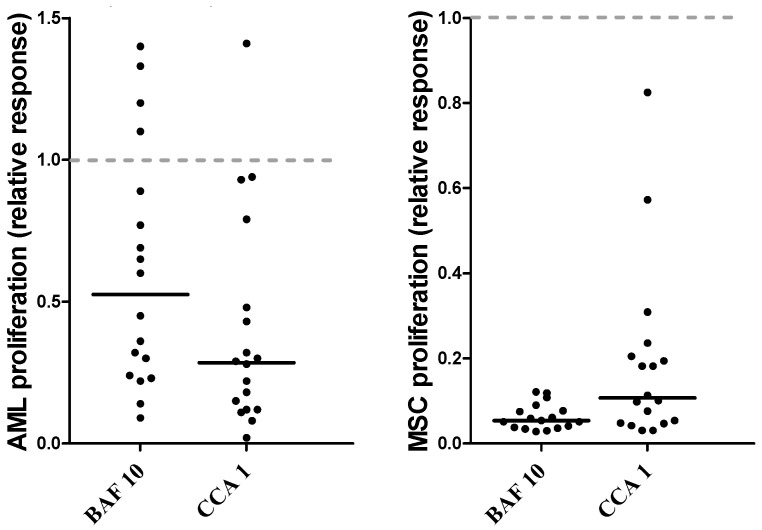
The effects of V-ATPase inhibitors on AML cells (**left**) and MSC proliferation (**right**) when testing AML cell/MSC co-cultures. Primary human AML cells were co-cultured in transwell cultures with normal MSCs, and proliferation of both cell types was assayed via ^3^H-thymidine incorporation. The two cell populations were separated by a semipermeable membrane. We investigated the effects of bafilomycin A1 10 nM (BAF10) and concanamycin A 1 nM (CCA1). All results are presented as the relative cell proliferation, i.e., the proliferation in drug-containing cultures relative to the proliferation in corresponding drug-free control cultures. We investigated AML cells derived from 18 unselected patients, and the MSCs were derived from the bone marrow of a healthy individual. The median AML cell proliferation in control cultures corresponded to 4271 cpm (range 1009–27,684), and the median MSC proliferation corresponded to 2451 cpm (range 1107–6424 cpm). The black lines indicate median responses, the dotted line indicates no effect of the agent (i.e., a relative response corresponding to 1.0).

The local soluble mediator network enhances the cell proliferation of primary human AML cells during co-culture of the leukemic cells with normal MSCs [43]. For this reason, we investigated whether V-ATPase inhibitors could inhibit AML cell proliferation even in the presence of leukemia-supporting MSCs. For these experiments, we used an experimental model where the two cell populations were separated by a semipermeable membrane where the AML-supporting MSC effect is mediated by intercellular crosstalk through the local network of the soluble mediator [43]. The median AML cell proliferation for the 18 investigated patients (see Table S15), when tested in the drug-free control cultures of these experiments, corresponded to 4271 cpm (range 1309–27,684 cpm), and when comparing the overall relative responses, both bafilomycin A1 (Figure 10 left part; binomial test, *p* = 0.0126) and concanamycin A (*p* = 0.000068) had significant antiproliferative effects on primary human AML cells, even in the presence of the leukemia-supporting MSCs.

We also investigated whether bafilomycin A1 10 nM and concanamycin A 1 nM had any effect on bone marrow MSC proliferation when these cells were co-cultured with the same 18 primary human AML cells (Table S15). The median MSC proliferation in the drug-free control cultures corresponded to 2451 cpm (range 1107–6424 cpm). Both bafilomycin A1 and concanamycin A (*p* < 0.005) significantly decreased the relative proliferative responses of the MSCs (Figure 9 right; binomial test with *p* = 0.000062 for both drugs).

Taken together, these observations show that (i) V-ATPase inhibition has an antiproliferative effect on primary AML cells even in the presence of leukemia-supporting MSCs; and (ii) the antiproliferative effect of V-ATPase inhibitors is not specific for AML cells but is also observed for non-leukemic bone marrow MSCs.

### 3.11. AML Cell Communication: Effects of V-ATPase Inhibitors on the Cytokine Network in MSC/AML Cell Co-Cultures; Secondary AML Is Associated with Increased Levels of Several Soluble Mediators

We investigated the effect of the two inhibitors on the release of 17 soluble mediators during co-culture of AML cells and MSCs; the leukemia cells derived from 18 unselected/consecutive patients were then examined (Table S15). The overall results are summarized in Table S16. It can be seen that bafilomycin A1 10 nM, but especially concanamycin A 1 nM, altered the levels of various soluble mediators; several mediators (including chemokines and interleukins) showed decreased levels in the presence of the V-ATPase inhibitors (Table S16), whereas increased levels where seen when AML cells alone were cultured in the presence of the inhibitors (see Table S10). However, there was still a wide variation between individual patients with regard to the soluble mediator levels both in the drug-free and drug-containing co-cultures.

**Figure 11:**
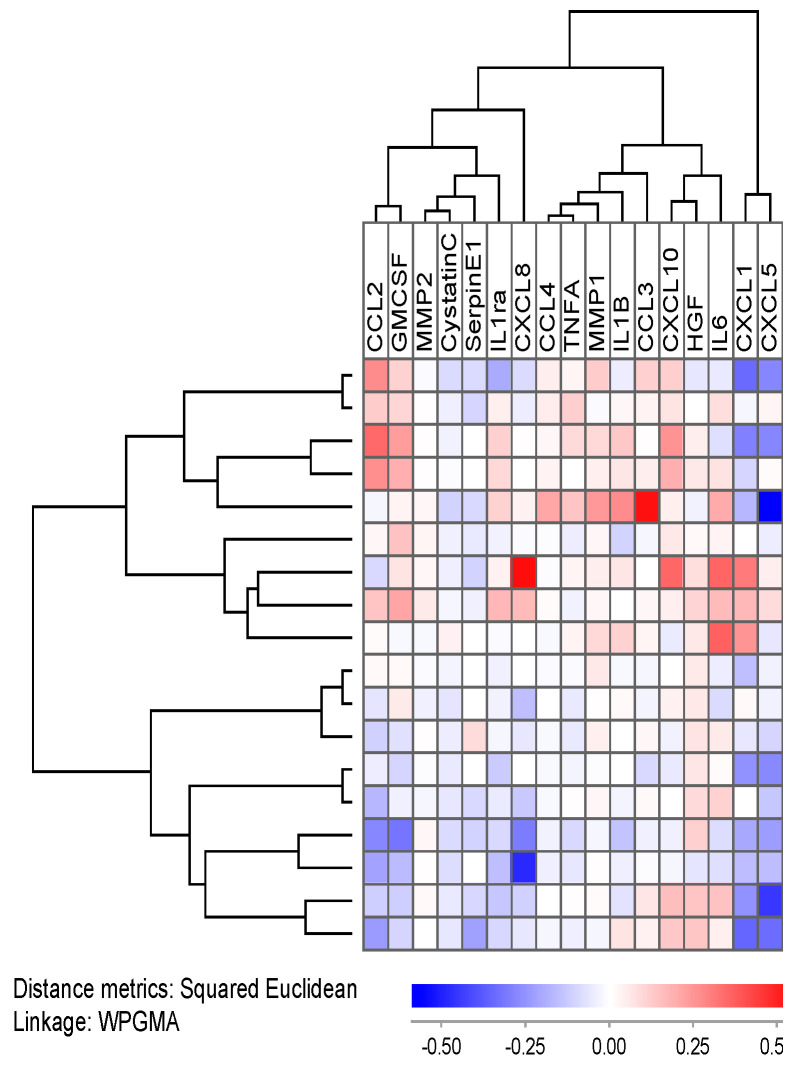
The cytokine network in transwell co-cultures of primary AML cells and normal MSCs. Primary AML cells were derived from 18 unselected patients, and the levels of 17 mediators were determined in the supernatants harvested after 48 h of co-culture. This clustering is based on the relative mediator levels, i.e., the levels in supernatants derived from inhibitor-containing co-cultures relative to the levels in supernatants derived from drug-free control cultures. Bafilomycin (**left**) and concanamycin (**right**) were tested at a final concentration of 10 nM.

We also investigated the effects of concanamycin A 1 nM on the co-culture mediator levels for individual patients. These analyses were based on the relative responses, i.e., the level of a certain mediator in drug-containing co-cultures relative to the levels of the same mediator in the corresponding drug-free control culture. The results for concanamycin A are presented in Figure 11. The effect of concanamycin A varied between patients; the upper main patient cluster included nine patients with increased levels for several mediators in the presence on concanamycin A, whereas the nine patients in the lower main cluster showed decreased levels in drug-containing co-cultures for several mediators. The clinical and biological characteristics of the patients in the two main clusters are compared in Table S15, and it can be seen that the upper main cluster included a higher fraction of patients with secondary AML than the lower main cluster (five versus no patients; Fisher’s exact test, *p* = 0.294).

To summarize, V-ATPase inhibitors can modulate the extracellular network of soluble mediators both for primary AML cells cultured alone and when AML cells are co-cultured with MSCs. However, there is a considerable variation between AML patients with regard to the capacity of their leukemic cells to constitutively release soluble mediator (for several mediators, up to 100-fold difference; see Figure S2). These variations between individual patients are generally larger than the differences induced by the V-ATPase inhibitors (often less than 3-fold; see Figure 9 and Figure S5). The effects of bafilomycin A1 on soluble mediators in MSC/AML cell co-cultures are even smaller (generally less than 2-fold; see Figure 11). Thus, the variation between patients with regard to the AML cell capacity of constitutive soluble mediator release in monocultures is probably more important than the relatively small modulations caused by the V-ATPase inhibition (Figures S3 and S4). The most important observation from our cytokine studies is possibly that these results further confirm that there is a functional difference between secondary and de novo AML cells with regard to the biological effect of V-ATPase inhibition.

### 3.12. Animal Models: Bafilomycin Monotherapy Shows Dose-Dependent Hematological Toxicity in Mice

**Figure 12:**
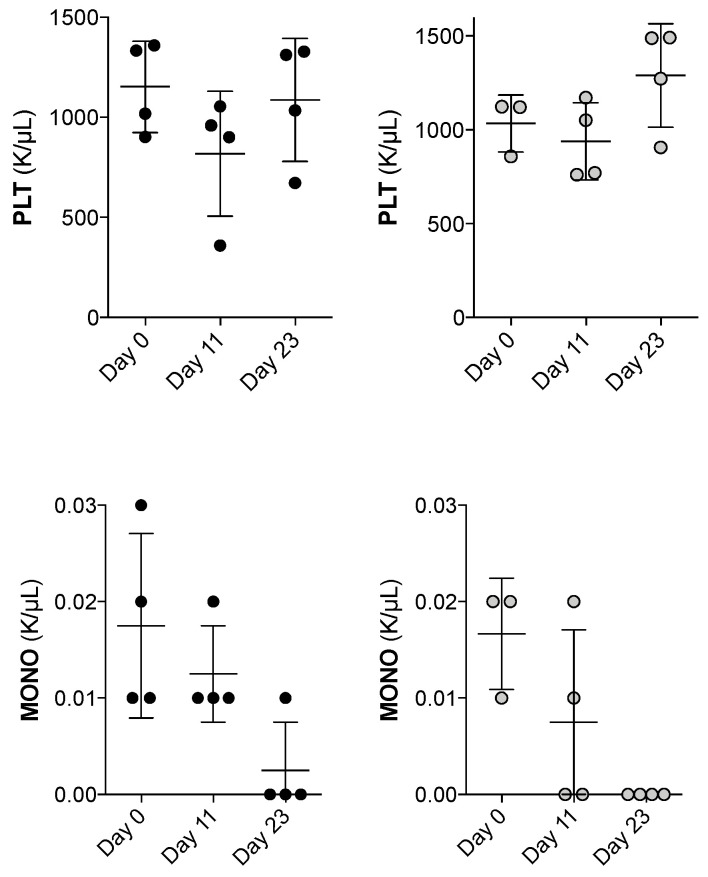
The effect of bafilomycin 0.1 (**left**) and 1.0 mg/kg (**right**) on platelet and monocyte peripheral blood counts. The animals (four mice for each concentration) were treated with bafilomycin for days 1–3 and 8–10, and peripheral blood cell counts were determined on day 0 (pretreatment), day 11 immediately after the second treatment and day 23 during regeneration. The mean and standard deviation is also indicated in the figure (lines) together with the measured values (black and open dots).

For the toxicity studies, bafilomycin was administered as indicated in Figure S8; the drug was administered for three consecutive days (0.1, 1.0 and 2.0 mg/kg on days 1–3 and days 8–10, and normal peripheral blood cell counts were evaluated on day 0 (before treatment) and on days 11 (the day after end of treatment) and days 23. Each group included four mice; three of the four mice receiving 2 mg/kg died early, consistent with the toxicity, and for this reason, we only present the results for the two lower doses. First, the body weight did not change during the observation period. Second, the hemoglobin level, erythrocyte count and reticulocyte count were stable during the 23 days observation period (Figure S9), whereas there was a transient reduction in the platelet count for bafilomycin 1.0 mg/kg with normalization on day 23 (Figure 12, upper part). Third, the total white blood cell, neutrophil and lymphocyte counts were stable during the treatment period (Figure S10, presentation of the complete data), but for both bafilomycin 0.1 and 1.0 mg/kg, we observed a significant reduction in monocyte counts, and these levels remained low even on day 23 (Figure 12, lower part). Finally, three of the four mice receiving bafilomycin A1 2.0 mg/kg died early after drug administration consistent with toxicity from a high maximal concentration of bafilomycin A1, whereas for the only surviving mouse receiving this dose, we observed a gradual reduction during the whole observation period, with decreased levels of erythrocyte parameters (hemoglobin level, erythrocyte and reticulocyte counts) and the levels of circulating total leukocytes, neutrophils and platelet counts.

No additional organ toxicities were observed for the mice receiving bafilomycin A1 0.1 or 1.0 mg/kg during the entire observation period.

### 3.13. Animal Models: Bafilomycin Monotherapy Has Only Weak Antileukemic Effects on Xenografted AML Cells

The effect of bafilomycin alone (daily doses 1 mg/kg) on xenografted MV4-11 cells was tested; during the experiment, we evaluated body weight, AML progression by bioluminescence, with quantification of leukemia cell burden, and survival. Drug administration was started 20 days post-transplant to allow engraftment of the leukemic cells, and the drug was administered for 3 days every week (Q.Dx3) for the next three weeks, i.e., the overall observation period was five weeks. This pilot study included three mice in each group, and after five weeks, neither the body weight, the leukemia cell burden evaluated by bioluminescence imaging nor the survival (Figure S11) differed between the two groups, as was expected based on our experience with this AML model [46].

**Figure 13:**
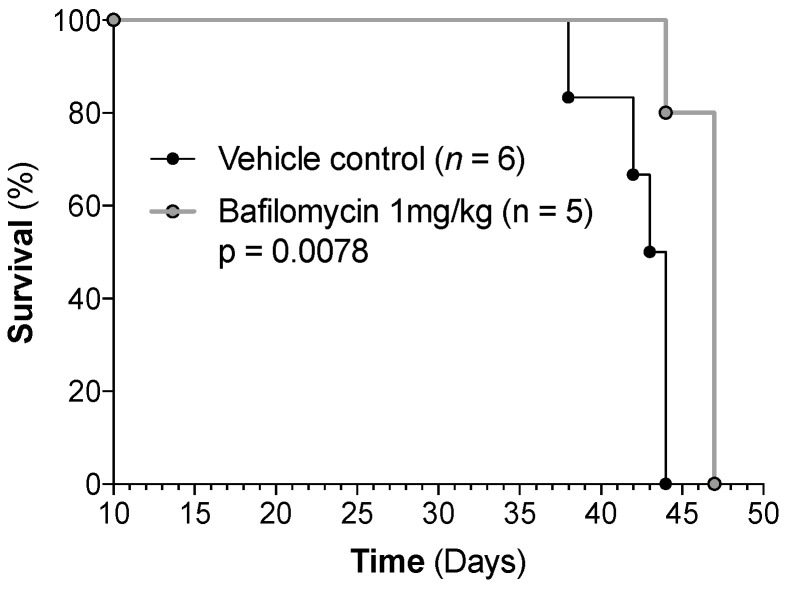
The antileukemic effect of bafilomycin monotherapy in vivo. Survival data presented in a Kaplan–-Meyer curve illustrating survival in vehicle control animals (*n* = 6) and bafilomycin monotherapy (*n* = 5) in the HL-60 orthotopic model of AML (log rank *p* = 0.0078 for bafilomycin 1 mg/kg vs. vehicle controls).

The effect of bafilomycin alone (daily doses 1 mg/kg) on xenografted HL-60 cells was also tested by using the same drug regimen as for the MV4-11 xenograft model. In this experiment, bafilomycin treatment was initiated on day 13 post-transplant, and animals received four cycles of therapy before the first control group animals reached endpoint criteria. Six mice were included per group, though one animal in the bafilomycin group was lost prematurely (day 28), likely due to the toxicity associated with the drug administration procedure because leukemic burden, as determined by bioluminescence imaging, was comparable with other animals within the group. Interestingly, the survival curve (Figure 13) indicates a modest antileukemic effect of bafilomycin monotherapy (*p* = 0.0078). However, this effect was not reflected in bioluminescence imaging, where no significant difference was observed between groups (Figure S12). Thus, the animals developed expected signs of progressive/advanced leukemia and had to be euthanized according to the predefined standardized criteria/endpoints described in Section 2.10. No additional signs of organ toxicity were identified by the regular clinical examination or the postmortem examination/autopsy.

To conclude, our studies of two different animal models indicate that there is also an in vivo diversity in the antileukemic effect of bafilomycin, but we would emphasize that the antileukemic effect of bafilomycin alone in the HL-60 model is weak and its clinical relevance should be regarded as uncertain. Even though only three mice were examined in each group of MV4-11 xenografted mice, the clinical course of the leukemic mice was as would be expected from previous studies [46], and neither body weight registration, imaging of leukemia burden nor survival showed any significant difference between the two groups. Thus, the results from our MV4-11 experiments (as well as previous studies of the MOLM13 cell line [28]) are also consistent with the conclusion that bafilomycin A1 monotherapy has no or only a weak antileukemic effect.

## 4. Discussion

V-ATPase is regarded as a possible therapeutic target in cancer treatment [3,4,8,9,10,11,70,71,72], and our present study suggests that V-ATPase inhibition is also a possible strategy in AML. We observed that V-ATPase inhibitors have antiproliferative (Section 3.1, Section 3.2, Section 3.3, Section 3.4 and Section 3.5) as well as proapoptotic (Section 3.7 and Section 3.8) effects and modulate the communication between leukemic and stromal cells (Section 3.9, Section 3.10 and Section 3.11). Strong antiproliferative effects together with a specific mRNA expression profile (Section 3.6) of the V-ATPase interactome are observed, especially for secondary AML. Furthermore, our animal studies suggest that the toxicity of V-ATPase inhibition is acceptable, although the possibility of hematological toxicity needs further evaluation (Section 3.12 and Section 3.13).

We investigated enriched primary human AML cells derived from consecutive and thereby unselected patients. Our hospital is responsible for diagnosis and treatment of AML in a defined geographical area, and the study should therefore be regarded as population-based. However, we only included patients with a relatively high level of circulating leukemic cells [36,38,73]; enriched AML cells could therefore be prepared by standardized gradient separation alone. Despite this, we regard our patients to be representative with regard to clinical chemosensitivity because the association between circulating blast level and clinical chemosensitivity is relatively weak [74]. Finally, we used cryopreserved AML cells [40,69], and this allowed us to compare different experiments (e.g., control cultures showing expected results) and to reduce interpatient variations by including several patients in each experimental set-up.

AML cell populations have hierarchical organization and include a minority of clonogenic cells (often <1% of the population) together with a small minority of leukemic stem cells [75]. Even though AML relapse is thought to be derived from AML stem cells, one would still regard it as relevant to investigate chemosensitivity for the whole AML cell population. First, all cells will probably have the same fundamental genetic abnormalities [1,2]. Second, the chemosensitivity of the whole AML cell population (i.e., slow reduction of AML cells after induction therapy) reflects an increased relapse risk [37]. Even the general modulation of intracellular signaling of the total AML cell population 24 h post-chemotherapy is a predictor of long-term patient survival [76]. Third, the gene expression/epigenetic/proteomic profiles of the total pretherapy AML cell populations reflect clinical chemosensitivity/relapse risk [77,78,79]. Finally, the majority of more mature AML cells have a main influence on the AML cell microenvironment (e.g., the local cytokine network). For these reasons, a combination of a standardized cell separation procedure and examination of the total AML cell population was used as a methodological strategy for most of our experiments.

Our utilized V-ATPase inhibitor concentrations were based on dose–response experiments, and concanamycin A then seemed more potent when testing equimolar doses. We selected concentrations that reflected the variation in antiproliferative effects between patients (Section 3.2). Studies in neuronal cells suggest that inhibition of the fusion between lysosomes and autophagosomes is seen at concentrations ≥ 10 nM [20,26], whereas effects at concentrations ≤ 1 nM are due to other cellular mechanisms, e.g., altered intracellular signaling, membrane structure or intracellular trafficking [11,12,13,14,15,75].

Both bafilomycin A1 and chloroquine inhibit autophagy in human AML cells [28,64,65,66,80,81]. We observed a similar wide variation between the antiproliferative effects of these two drugs and a significant covariation with regard to the strength of their antiproliferative effects. This covariation supports the hypothesis that their shared effect of autophagy inhibition contributes to their antiproliferative effects [4,5]. However, there is an ongoing process of spontaneous endoplasmic stress-induced apoptosis during AML cell culture that also may contribute to covariation [69], although this is probably less important when relative responses are compared. The difference between the two agents in antiproliferative effect for certain exceptional patients can be explained by the additional effects of bafilomycin A1 on intracellular signaling (e.g., Wnt/β-cathenin, Notch, PI3K/Akt/mTOR signaling) [4,11,12,13,14,15], intracellular trafficking and receptor recycling [4], cellular nutrition and regulation of cellular metabolism [4] and/or on the regulation of other forms of programmed cell death [5].

V-ATPase mediates apoptosis resistance/chemoresistance in various malignant cells, but it can also have proapoptotic functions under certain conditions [5,18,19] and be a regulator of other forms of cell death, including anoikis, alkaliptosis, ferroptosis and lysosome-dependent cell death [5]. Our two V-ATPase inhibitors had significant antiproliferative and anti-survival effects when analyzing the overall results, even though the strength of these effects varied between patients. Furthermore, we could not detect any association between the antiproliferative and proapoptotic effects; possible explanations for this lack of association could be that the two effects (i) are caused by different molecular mechanisms or (ii) reflect pharmacological effects on different AML cell subsets, or else (iii) the covariation is masked due to additional spontaneous in vitro apoptosis [69]. BafilomycinA1 and concanamycin A had divergent effects on the constitutive release of soluble mediators by AML cells; both agents caused generally decreased levels for a minor patient subset but increased levels for most patients. This heterogeneity showed no strong association with any clinical or biological characteristics or with antiproliferative/proapoptotic effects. Studies in other cell types (including malignant cells) have demonstrated that V-ATPase inhibition can affect soluble mediators release through various mechanisms [82,83,84,85] including increased mediator accumulation in the endoplasmic reticulum [82], altered intracellular signaling (e.g., altered SAPK/JNK activation) [83] and inhibition of intracellular proteolytic cleavage of pro-cytokines [84,85]. Thus, differences between individual patients with regard to the targeted cellular mechanism(s) can possibly explain the variation between patients with regard to the effect of V-ATPase inhibition on soluble mediator release.

We investigated the effect of V-ATPase inhibition on the soluble mediator network in MSC/AML cell co-cultures. Hierarchical clustering analysis then identified two main patient subsets characterized by generally increased and decreased mediator levels, respectively. The high-release profile was associated with secondary AML, an association not seen when AML cells were cultured alone. Thus, the effects of V-ATPase inhibition on the extracellular mediator network of the AML cell microenvironment is further modulated by effects on neighboring non-leukemic cells, including MSCs but possibly also endothelial cells [15,86] and osteoblasts [87,88,89].

Our studies of AML cell monocultures showed that V-ATPase inhibition had a direct antiproliferative effect that was particularly strong for secondary AML. On the other hand, MSCs mediate AML supporting effects through their release of various soluble mediators [43], and secondary AML was associated with generally increased levels of extracellular cytokines/chemokines/growth factors in MSC/AML cell co-cultures (i.e., a potentially growth enhancing/antiapoptotic effect). However, the extracellular mediators showed a relatively small increase in the co-cultures (always <2-fold), and for this reason, we regard this indirect and potentially growth-enhancing effect to be relatively weak and less important than the direct antiproliferative effects of V-ATPase inhibition.

Secondary AML is a heterogeneous group [54] that includes AML as a complication after chemotherapy/radiotherapy and AML occurring after antecedent myeloid disease [1,2]. The patients are characterized by older age, increased frequency of high-risk cytogenetic abnormal, different mutational profiles [1,53,54,90] and increased risk of leukemia relapse after conventional intensive chemotherapy [50,52,53,55] and allogeneic stem cell transplantation [56,57]. Due to their common inferior outcome, they have been handled as a separate group in many clinical studies [53,55,56,57,59]. For this reason, they were also handled as a single group our present study.

Patients with secondary AML (i.e., a subset with adverse prognosis) showed several statistically significant differences from patients with de novo AML and were associated with a strong antiproliferative effect of V-ATPase inhibition, a different V-ATPase interactome mRNA profile and increased levels of several extracellular soluble mediators in bafilomycin-containing MSC/AML cell co-cultures. A previous proteomic study also showed that a different patient subset with adverse prognosis (i.e., later chemoresistant relapse) showed low protein levels of certain ATP transporters and strong antiproliferative effects of V-ATPase inhibitors in pretreatment AML cells compared with patients later becoming long-term AML-free survivors after [35]. Thus, both these high-risk patient subsets have different/altered V-ATPase expression/function as a part of their pretherapy AML cell phenotype.

Our conclusion that the effect of V-ATPase inhibition differs between de novo and secondary AML has to be made with care, even though it was observed via various experimental approaches. Our study population included mainly patients with secondary AML following MDS/myeloproliferative neoplasias but only two patients with previous chemotherapy (see Table S1). Furthermore, secondary AML is a heterogeneous group even with regard to adverse prognosis [52,53,54,55,56,57], where at least NPM1-mutated chemotherapy-associated secondary AML seem to have a better prognosis, similar to de novo NPM1-mutated AML [91,92,93]. Our present observations should therefore be confirmed in larger studies that allow further comparisons of various subsets of secondary AML.

We conducted a proteomic and phosphoproteomic comparison of pretherapy AML cells showing weak and strong antiproliferative effects of V-ATPase inhibitors. Several molecules that influence the same cellular structures/processes as V-ATPase differed significantly between these two contrasting patient groups. The proteomic comparison identified the differential expression of several proteins involved in molecular (transmembrane) transport, degranulation and cytoskeletal function. On the other hand, the phosphoproteomic profiling identified differences in RNA metabolism/function, cytoskeletal function and growth regulation, and these differences partly reflected increased CK2 activity (Figure 5). The final functional effects of V-ATPase inhibition thus seem to depend on the intracellular context of V-ATPase (e.g., regulation of gene expression, cytoskeletal functions and intracellular transport/trafficking). In contrast, our previous study of pretherapy AML cells showed decreased levels of several V-ATPase components, together with strong antiproliferative effects of V-ATPase inhibitors for patients with adverse prognosis (i.e., later relapse after intensive therapy) [35]. We could not detect similar quantitative V-ATPase differences in our present study when comparing patients with strong (including several patients with secondary AML) versus weak antiproliferative effects of V-ATPase inhibitors. Possible explanations for this difference could be differences with regard to patient inclusion (selection of only younger patients fit for intensive treatment in the previous study) or the lower number of patients our present study. Finally, the association of CK2 activity with a high-risk AML cell phenotype is similar to our previous phosphoproteomic study [35].

Our proteomic comparison identified four potential biomarkers with increased levels in AML cells showing strong antiproliferative effects of V-ATPase inhibition. First, transferrin receptor levels possibly reflect differences in iron metabolism that are important for chemosensitivity [94,95], extracellular mediator release and non-relapse survival after intensive therapy [96,97,98,99]. Second, Chitinase 3-like level is associated with survival for AML patients receiving intensive therapy [100,101]; cellular expression or systemic levels of the soluble form may function as markers of susceptibility to V-ATPase inhibition. Third, the secreted protein C type lectin domain containing 11A functions as a hematopoietic growth factor and high expression is associated with prognosis in AML [102,103]. Finally, the protease inhibitor Serpin family A member 3 is expressed together with several other proteases and protease regulators in AML [104]. The combination of such markers (cellular expression or systemic levels of soluble forms) should be further investigated in future clinical studies.

We combined V-ATPase inhibition with cytarabine that was tested at concentrations corresponding to the in vivo levels during low-dose cytarabine therapy [105]; the observed additive antiproliferative effects suggest that this combined treatment should be further explored for elderly or unfit AML patients. However, the possible combination of V-ATPase inhibitors with higher cytarabine doses or with other cytotoxic drugs used in intensive AML therapy needs to be further investigated.

We investigated the in vivo toxicity of bafilomycin A1 that can be administered intraperitoneally without severe toxicity [7,70,71,72]. Our observations in healthy mice receiving bafilomycin monotherapy suggest that V-ATPase inhibition is associated with a dose-dependent risk of hematological toxicity, especially thrombocytopenia but also persistent monocytopenia that is uncommon in conventional AML therapy [106,107]. Furthermore, animal studies have shown that the V-ATPase inhibitors concanamycin A and B inhibit the cytotoxic function and reduce the levels of circulating CD8^+^ cytotoxic T lymphocytes, whereas the levels/functions of CD4^+^ helper T lymphocytes and B lymphocytes are not affected [108,109]. The T cell effects were associated with modulation of autophagy, and some of these effects were further enhanced by aging [110]. Previous studies have described long-lasting CD4^+^ T lymphopenia after cytotoxic anticancer therapy [111]. A pan-T cell defect may thus be a risk after combined treatment with V-ATPase inhibitors and conventional cytotoxic drugs. Finally, our observations also suggest that V-ATPase inhibition may, in addition, be considered for treatment of the monocyte-mediated component of cancer-associated or cancer cell supporting inflammation [112,113,114].

Bafilomycin A1 monotherapy following xenotransplantation of MV4-11 and HL-60 AML cells had limited or no effect on subsequent leukemia progression (Section 3.13). Another study described no effect of bafilomycin on xenoengraftment of the MOLM13 AML cell line [28], but this study showed an additional antileukemic effect of bafilomycin when combined with cytarabine. Taken together, these observations suggest that bafilomycin monotherapy has a limited antileukemic effect, although our HL-60 experiments suggest that AML cell lines are heterogeneous similar to the patient heterogeneity. However, AML cell lines may not be representative for primary AML cells because they have extensive cytogenetic abnormalities [115], whereas primary cells usually have none or only a limited number of cytogenetic abnormalities [1,2].

Our present study has limitations that should be addressed in future studies. First, we did not investigate effects of V-ATPase inhibitors on AML stem cells that are thought to be responsible for chemoresistant leukemia relapse [36], even though observations for the whole AML cell population are also relevant with regard to relapse risk (see page 25). Additional studies on AML stem cells interactions with the non-leukemic AML-supporting cells in the stem cell niches should also be conducted [15,86,87,88,89]. Second, a larger study investigating the biological heterogeneity of secondary AML and the molecular mechanisms behind the effects of V-ATPase inhibition in this patient subset is also needed. Third, the combination of V-ATPase inhibitors with other antileukemic drugs, not only cytarabine, is of interest, especially the combination with venetoclax [116,117]. Finally, the mechanisms of regulated AML cell death and their modulation/regulation by V-ATPase need further characterization [4,5]. Several forms of programmed cell death may be operative in human AML [118,119,120,121,122,123,124,125,126,127,128,129,130,131], including endoplasmic reticulum stress-induced apoptosis [69]; possibly autophagy-associated apoptosis, although autophagy is generally regarded as a survival mechanism [120]; ferroptosis due to lethal iron accumulation [5,118,119,120,121,122,123,124]; inflammatory necroptosis [124,125,126,127] and pyroptosis [128,129]; oncosis [130]; apoptosis-like anoikis due to loss of cell anchorage [5,131]; and pH-dependent alkaliptosis [5]. Thus, programmed cell death in AML cells can have a mixed etiology, including apoptosis (which seems particularly important), together with other forms of programmed cell death. The contribution of these various forms is not reflected in our present experimental models, and the balance between various forms may differ between patients. Both in vitro-associated endoplasmic reticulum stress [69] and V-ATPase inhibition influence AML cell viability in our experimental models and contribute to the variation between patients in our viability studies.

## 5. Conclusions

Our experimental studies show that V-ATPase inhibition has antiproliferative, proapoptotic and cytokine-modulating effects on primary human AML cells. Both our present study and a small previous study [28] support the hypothesis that the effects of V-ATPase inhibitors differ between patients. Strong antiproliferative effects are seen especially for patients with secondary AML; these patients also have a specific mRNA V-ATPase interactome profile and a specific cytokine network profile when AML cells are co-cultured with AML-supporting MSCs. Furthermore, our animal studies suggest that future clinical studies have to carefully evaluate the possibility of hematological toxicity. A final question is how to use V-ATPase inhibition in future clinical studies. Our present study suggests that monotherapy has a limited antileukemic activity, but combination therapy with conventional cytotoxic drugs may be a possibility. Another possibility is combination with other targeted therapies, including venetoclax [116,117]. Our phosphoproteomic studies suggest that a strong antiproliferative effect of V-ATPase inhibition is associated with high CK2 activity (Figure 4). Combination of V-ATPase and CK2 inhibition is further supported by several observations: (i) CK2 inhibition will cause an additional inhibition of several intracellular signaling pathways that are important in AML [132]; (ii) high CK2 activity seems to be a part of a chemoresistant AML cell phenotype and is associated with clinical chemoresistance and reduced survival [133]; and (iii) the initial clinical studies suggest that CK2 inhibition has an acceptable toxicity [134,135].

## Figures and Tables

**Table 1:** Biological and clinical characteristics of the 80 AML patients included in the study. Detailed information about the clinical and biological characteristics and the diagnosis of secondary AML is given in Table S1.

Patient Characteristics		Cell Morphology		Cell Genetics	
***Age***		*FAB classification*		*Cytogenetics*	
Median (yrs.)	64	M0	7	Favorable	6
Range (yrs.)	18–87	M1	16	Adverse	8
		M2	18	Intermediate	18
*Gender*		M4	19	Normal	40
Females	35	M5	15	n.d.	8
Males	45	n.d.	5		
				*Flt3 mutations*	
				ITD	20
De novo *AML*	58	*CD34 receptor*		Wild-type	44
*Secondary AML* *Relapse*	20 3	Negative (≤20%) Positive (>20%)	24 48	n.d.	16
		n.d.	8	*NPM1 mutations*	
				Mutated	24
				Wild-type	39
				n.d.	17

n.d.: not determined.

**Table 2:** The effect of the V-ATPase inhibitors, bafilomycin A1 (BAF) and concanamycin A (CCA), on cytokine-dependent proliferation of primary human AML cells; a summary of the overall results. Eighty patients were tested in the ^3^H-thymidin incorporation assay; seventy of them showed detectable proliferation corresponding to ^3^H-thymidine incorporation > 1000 cpm in the drug-free controls. The table presents a summary of the results for these 70 patients with detectable cytokine-dependent proliferation. The left part of the table presents the results as the nuclear incorporation of ^3^H-thymidine (counts per minute, cpm) together with the results from the statistical analysis, whereas the right part of the table presents the results as the relative responses, i.e., the nuclear radioactivity for drug-containing cultures relative to the nuclear radioactivity in the corresponding drug-free control cultures. The Wilcoxon’s test for paired samples was used for all statistical comparisons, and the median cpm of triplicate cultures was used for all calculations and statistical comparisons (nd/not detectable proliferation).

Drugs Added	Proliferation in Drug-Free Controls and in Drug-Containing Cultures (cpm)			Proliferation in Drug-Containing Relative to Proliferation in Corresponding Drug-Free Controls (Relative Response)	
	Median	Range	*p*-Value	Median	Range
Drug-free control	11,683	1317–173,197		1.00	-
BAF 10 nM	3177	nd-81,388	<0.0005	0.30	0.03–1.30
BAF 5 nM	7208	nd-137,101	<0.0005	0.82	0.19–1.40
BAF 1 nM	11,505	nd-161,956	0.001	0.96	0.52–1.25
CCA 10 nM	<1000 cpm	<1000 cpm	<0.0005	0.09	0.01–0.98
CCA 5 nM	<1000 cpm	<1000 cpm	<0.0005	0.9	0.01–0.98
CCA 1 nM	<1000 cpm	<1000 cpm-66,606	<0.0005	0.13	0.01–1.74

**Table 3:** The antiproliferative effect of combining V-ATPase inhibition with cytarabine: a summary of the results for primary AML cells derived from 22 of the 80 patients included in the study. All 22 patients showed detectable proliferation (corresponding to >1000 cpm) in control cultures as well as in the presence of concanamycin A 1 nM and cytarabine 1 μM. The proliferation was examined in the ^3^H-thymidine incorporation assay, and the median cpm of triplicate cultures was used for all statistical calculations and for the presentation of the results in the table. The results are presented as the median and range of the nuclear radioactivity (cpm), and the corresponding relative responses (median and range) are given in parentheses. The Wilcoxon’s test for paired samples was used for all statistical comparisons, and the *p*-values refer to statistical analyses based on the cpm data.

Drugs Added	Proliferation		*p*-Value		
	Median (cpm)	Range (cpm)	Versus Drug- Free Control	Versus Combination	Versus Concanamycin Alone
Drug-free control	20,315	3530–162,418			
Cytarabine	8482 (0.74)	835–69,150 (0.01–1.46)	0.00194	<0.00001	0.0188
Concanamycin A	3766 (0.24)	1167–66,606 (0.02–1.74)	0.00086	0.0006	
Concanamycin A + cytarabine	1625 (0.17)	427–25,673 (0.01–0.64)	<0.00001		

**Table 4:** The effect of AML cell viability of the V-ATPase inhibitors bafilomycin A1 10 nM and concanamycin A 10 nM: a summary of the overall results. AML cells derived from 80 patients were tested after 48 h of incubation in cytokine-supplemented medium with and without drugs. The results are presented as the percentage of viable Annexin^−^PI^−^ cells, and the table summarized the overall results for the 80 patients (median and variation range). The Wilcoxon’s test for paired samples was used for all statistical comparisons, and Pearson’s test was used for correlation analyses.

Drugs Added	Viability (%)		*p*-Values for the Various Statistical Comparisons			
	Median	Range	Versus Drug- Free Control	Versus Concanamycin	Versus Cytarabine	Correlation with Control
Drug-free control	54.1	2.0–97.2				
Bafilomycin A1 10 nM	51.0	0.6–95.2	<0.00001	<0.00001	0.00112	<0.00001 (r = 0.98)
Concanamycin A 10 nM	16.4	1.9–97.2	<0.00001	-	<0.00001	<0.00001 (r = 0.74)
Cytarabine 10 nM	53.1	2.6–97.9	<0.00001	<0.00001	-	<0.00001 (r = 0.99)

## Data Availability

Data can only be made available by request to the corresponding author due to Norwegian privacy restrictions.

## Supplementary Materials

The following supporting information can be downloaded at https://www.mdpi.com/article/10.3390/jcm12175546/s1: Table S1: Clinical and biological characteristics of the 80 consecutive patients included in the study; Table S2: Clinical and biological characteristics of patients showing a strong antiproliferative effect of V-ATPase inhibition (bafilomycin 10 nM); Table S3: The results from molecular genetic analysis from 33 AML patients; Table S4: The results from molecular genetic analyses of primary AML cells derived from 33 AML patients; Table S5: Differentially expressed proteins when comparing AML cells showing strong (eight patients, relative response ≤ 0.30) and weak (seven patients, relative response ≥ 0.60) antiproliferative effect of the V-ATPase inhibitor bafilomycin A1 10 nM; Table S6. Unsupervised hierarchical clustering of differentially expressed proteins when comparing AML patient cells showing string versus weak antiproliferative effects of V-ATPase inhibition; Table S7: Differentially expressed proteins when comparing primary AML cells showing a weak and a strong antiproliferative effect of V-ATPase inhibition (bafilomycin A1 10 nM); Table S8: Differentially expressed protein phosphorylation sites when comparing AML cells showing a weak (seven patients, relative response ≥ 0.60) and strong (eight patients, relative response ≤ 0.30) antiproliferative effect of the V-ATPase inhibitor bafilomycin A1 10 nM; Table S9. Unsupervised hierarchical clustering of differentially expressed phosphosites. when comparing AML patient cells showing string versus weak antiproliferative effects of V-ATPase inhibition. Table S10: Gene expression analysis of the V-ATPase interactome; analyses of the results for 32 unselected/consecutive AML patients; Table S11: The effect of V-ATPase inhibitors on the viability of primary human AML cells derived from all 80 patients included in our study; Table S12: Constitutive release of soluble mediators by primary human AML cells; comparison of cultures with the two V-ATPase inhibitors bafilomycin A1 and concanamycin A (10 nM) with drug-free medium controls; Table S13: Effects of concanamycin A on the constitutive cytokine release by primary human AML cells; Table S14: Effects of bafilomycin A1 on the constitutive cytokine release by primary human AML cells; Table S15: Clinical and biological characteristics of the 18 patients included in the studies soluble mediator release during co-culture of primary AML cells and normal MACs; Table S16; Constitutive release of soluble mediators by MSC co-culture of primary human AML cells derived from 18 unselected patients; Figure S1: A comparison of the global primary AML cell proteome for leukemic cells characterized by either a strong antiproliferative effect of bafilomycin A1 10 nM (eight patients, relative response ≤ 0.30) or a weak effect (seven patients, ≥0.60); Figure S2: The constitutive release of soluble mediators by primary AML cells derived from 80 patients; Figure S3: The patient profiles of absolute soluble mediator levels when primary AML cells were cultured either in medium alone, in the presence of bafilomycin 10 nM or concanamycin A 10 nM; Figure S4: The patient profiles of absolute soluble mediator levels when primary AML cells were cultured in the presence of bafilomycin 10 nM (LEFT) or concanamycin A 10 nM (RIGHT). Figure S5: Effects of bafilomycin A1 on the constitutive cytokine release by primary human AML cells; Figure S6: Cytarabine has only minor effects on the soluble mediator release profile of primary human AML cells; Figure S7: V-ATPase inhibition causes an increased constitutive soluble mediator release by primary human AML cells also in the presence of cytarabine; Figure S8: Evaluation of in vivo bafilomycin toxicity; studies of normal peripheral blood cell counts; Figure S9: Effects on bafilomycin on erythrocytes and platelets; Figure S10: Effects on bafilomycin on normal leukocytes; Figure S11: The effect of bafilomycin on xenografted MV411 AML cells, studies of survival and disease burden as determined by bioluminescence (BLI) intensity, photons per second (p/sec); Figure S12. The effect of bafilomycin on bioluminescence imaging of xenografted HL-60 AML cells. References [94,95,96,100,101,102,103,131,136,137,138,139,140,141,142,143,144,145,146,147,148,149] are cited in Supplementary Materials.
