# Supplementary material for: Vacuolar ATPase Is a Possible Therapeutic Target in Acute Myeloid Leukemia: Focus on Patient Heterogeneity and Treatment Toxicity
Source: J Clin Med. 2023 Aug 25;12(17):5546. doi: 10.3390/jcm12175546 (PMC10488188; doi:10.3390/jcm12175546)
Supplement: Supplementary file 1 [file jcm-12-05546-s001.zip › jcm-2524297-supplementary.pdf]

SUPPLEMENTARY INFORMATION

VACUOLAR ATP-ASE IN ACUTE MYELOID LEUKEMIA (AML): PHARMACOLOGICAL INHIBITION HAS ANTIPROLIFERATIVE AND PROAPOPTOTIC EFFECTS THAT DIFFER BETWEEN PATIENTS

Table S1: Clinical and biological characteristics of the 80 consecutive patients included in the study; **Table S2:** Clinical and biological characteristics of patients showing a strong antiproliferative effect of V-ATPase inhibition (bafilomycin 10 nM); **Table S3:** The results from molecular genetic analysis from 33 AML patients; **Table S4:** The results from molecular genetic analyses of primary AML cells derived from 33 AML patients; **Table S5:** Differentially expressed proteins when comparing AML cells showing strong (eight patients, relative response ≤0.30) and weak (seven patients, relative response ≥0.60) antiproliferative effect of the V-ATPase inhibitor bafilomycin A1 10 nM; **Table S6.** Unsupervised hierarchical clustering of differentially expressed proteins when comparing AML patient cells showing string versus weak antiproliferative effects of V-ATPase inhibition; **Table S7:** Differentially expressed proteins when comparing primary AML cells showing a weak and a strong antiproliferative effect of V-ATPase inhibition (bafilomycin A1 10 nM); **Table S8:** Differentially expressed protein phosphorylation sites when comparing AML cells showing a weak (seven patients, relative response ≥0.60) and strong (eight patients, relative response ≤0.30) antiproliferative effect of the V-ATPase inhibitor bafilomycin A1 10 nM; **Table S9.** Unsupervised hierarchical clustering of differentially expressed phosphosites. when comparing AML patient cells showing string versus weak antiproliferative effects of V-ATPase inhibition. **Table S10:** Gene expression analysis of the V-ATPase interactome; analyses of the results for 32 unselected/consecutive AML patients; **Table S11:** The effect of V-ATPase inhibitors on the viability of primary human AML cells derived from all 80 patients included in our study; **Table S12:** Constitutive release of soluble mediators by primary human AML cells; comparison of cultures with the two V-ATPase inhibitors bafilomycin A1 and concanamycin A (10 nM) with drug-free medium controls; **Table S13:** Effects of concanamycin A on the constitutive cytokine release by primary human AML cells; **Table S14:** Effects of bafilomycin A1 on the constitutive cytokine release by primary human AML cells; **Table S15:** Clinical and biological characteristics of the 18 patients included in the studies soluble mediator release during co-culture of primary AML cells and normal MACs; **Table S16;** Constitutive release of soluble mediators by MSC co-culture of primary human AML cells derived from 18 unselected patients; **Figure S1:** A comparison of the global primary AML cell proteome for leukemic cells characterized by either a strong antiproliferative effect of bafilomycin A1 10 nM (eight patients, relative response ≤0.30) or a weak effect (seven patients, ≥0.60); **Figure S2:** The constitutive release of soluble mediators by primary AML cells derived from 80 patients; **Figure S3:** The patient profiles of absolute soluble mediator levels when primary AML cells were cultured either in medium alone, in the presence of bafilomycin 10 nM or concanamycin A 10 nM; **Figure S4**: The patient profiles of absolute soluble mediator levels when primary AML cells were cultured in the presence of bafilomycin 10 nM (LEFT) or concanamycin A 10 nM. **Figure S5:** Effects of bafilomycin A1 on the constitutive cytokine release by primary human AML cells; **Figure S6:** Cytarabine has only minor effects on the soluble mediator release profile of primary human AML cells; **Figure S7:** V-ATPase inhibition causes an increased constitutive soluble mediator release by primary human AML cells also in the presence of cytarabine; **Figure S8:** Evaluation of in vivo bafilomycin toxicity; studies of normal peripheral blood cell counts; **Figure S9:** Effects on bafilomycin on erythrocytes and platelets; **Figure S10:** Effects on bafilomycin on normal leukocytes; **Figure S11:** The effect of bafilomycin on xenografted MV411 AML cells, studies of survival and disease burden as determined by bioluminescence (BLI) intensity, photons per second (p/sec); **Figure S12.** The effect of bafilomycin on bioluminescence imaging of xenografted HL-60 AML cells.

1.

Table S1. Clinical and biological characteristics of the 80 consecutive patients included in the study. Patients with MDS-associated mutations are marked with green. All patients with the diagnosis of secondary AML had a duration of at least four months from diagnosis of MDS/chronic myeloproliferative neoplasia or from previous chemotherapy until development of AML. The diagnosis of MDS and chronic myeloproliferative neoplasia was based on the World Health Organization (WHO) classification of myeloid neoplasms and acute leukemia.

Id	Sex	Age	Secondary/relapsed AML	FAB	Karyotype	FLT3	NPM-1	CD34
1	M	42		M2	Normal	ITD	wt	+
2	M	81	Secondary AML (PV)	M2	Monosomy 7/-7	wt	wt	-
3	F	80	Secondary AML (CMF)	M2	nt	nt	nt	+
4	M	82	Secondary AML (CMF)	M2	t(9;22)	wt	wt	+
5	M	60		M5	t(10;11), +8	wt	wt	nt
6	M	33		M4	Normal	ITD	INS	+
7	M	78	Secondary AML (CMML)	M4	+8	nt	nt	-
8	F	49	Relapse	M2	Complex	nt	nt	+
9	M	32		M2	Normal	wt	wt	+
10	F	51	Relapse	M0	Complex	wt	wt	+
11	M	78	Secondary AML (MDS)	M1	Nt	ITD	nt	+
22	M	24		M2	Complex	nt	wt	+
13	F	77	Secondary AML (MDS)	M1	Normal	wt	wt	+
14	M	46		M1	Normal	wt	INS	nt
15	M	46		M1	Normal	wt	INS	nt
16	M	68	Secondary AML (CMF)	M4	Normal	Asp835	wt	+
17	F	70		M4	Normal	wt	INS	-
18	F	71	Secondary AML (MDS)	nt	del(12)	nt	nt	+
19	F	86		M2	Nt	nt	nt	+
20	M	65		M5	t(16;16)	wt	wt	+
21	F	55		M2	Normal	ITD	INS	-
22	M	71	Secondary AML (chemotherapy)	M4/5	Nt	nt	nt	nt
23	F	18		M4	Inv16 (p13,1q22)	wt	wt	+
24	F	77	Secondary AML (MDS)	M1/2	Normal	ITD	INS	nt
25	M	71		nt	nt	nt	nt	nt
26	F	46		M1	inv16	wt	wt	+
27	M	65	Secondary AML (MDS)	M1	Normal	wt	INS	-
28	F	75	Secondary AML (CMF)	M2	nt	nt	nt	+
29	F	45		M4	Normal	wt	INS	-
30	F	78		M1	Normal	ITD	INS	-
31	F	67		M0	+21	wt	wt	+
32	M	60	Secondary AML (MDS)	M2	Normal	nt	nt	+
33	M	60		M4	del9	ITD	wt	+
34	F	59		M5	Normal	ITD	INS	-
35	M	41		M1	t(8;21) del9-20 -22 -3mar	wt	wt	+
36	M	35		M2	Normal	wt	wt	+
37	M	76	Secondary AML (MDS)	nt	Normal	nt	nt	+
38	F	82		M4	Normal	ITD	wt	+
39	M	72	Secondary AML (MDS)	M1	Complex	wt	nt	+
40	F	71	Secondary AML (MDS)	nt	t(1;5), t(2;3)	nt	nt	+
41	M	41		M4	Normal	wt	wt	nt
42	M	53		M0	pluss 13	wt	wt	+
43	M	82		nt	+8	wt	wt	+
44	M	58		M5	Normal	wt	wt	+
45	F	55		M5	Normal	ITD	INS	-
46	M	62		M1	Normal	wt	wt	+
47	M	65		M4	Normal	wt	INS	-
48	M	64		M5	Normal	wt	INS	-
49	M	48		M5	Normal	ITD	INS	-
50	M	78		M1	t(9;22) +8, +10,+12+19+20+21 der22	nt	nt	+
51	M	36		M4	3inv16	wt	wt	+
52	F	92	Secondary AML (chemotherapy)	M1	nt	nt	nt	-
53	F	59		M4	Normal	ITD	INS	-
54	M	61		M4	Normal	wt	wt	-
55	F	57		M4	Inv16	wt	wt	+
56	M	20		M2	Normal	ITD	wt	+
57	F	64		M2	Normal	ITD	INS	-
58	F	29		M5	Normal	ITD+Asp835	wt	+

59	F	63		M1	Normal	wt	wt	+
60	M	76	Secondary AML (CMML)	M5	del12, -7	nt	nt	+
61	F	74		M4	t(8;21)	ITD	INS	+
62	M	78		M0	Complex	wt	wt	-
63	F	68		M5	Normal	wt	INS	-
64	M	65		M5	Complex	wt	INS	nt
65	F	64	Relapse	M4	42-46, XX, -16,-22 [cp25]	wt	wt	+
66	M	72	Secondary AML (MDS)	M4	Normal	nt	nt	-
67	M	77		M2	+8, +6, +15, +20, +21, +22	ITD	wt	+
68	F	60		M5	Normal	ITD	INS	-
69	F	55		M1	Normal	ITD	INS	+
70	M	68		M1	Normal	wt	wt	+
71	M	54		M5	Normal	wt	INS	-
72	M	76		M0	Normal	wt	wt	+
73	M	62		M4	Trisomy 8	wt	wt	+
74	F	87		M0	del5 (q13q33)	wt	wt	+
75	M	59	Secondary AML (CMML)	M5	del20, +8	ITD	wt	+
76	F	71		M0	Normal	wt	INS	-
77	F	46		M2	i(13) subclone	wt	wt	+
78	F	77		M1	nt	wt	INS	-
79	F	50		M2	-7, t(3;3)	wt	wt	+
80	M	19		M5	Normal	wt	wt	-

Abbreviations: CMF, chronic myelofibrosis; CMML, chronic myelomonocytic leukemia; MDS, myelodysplastic syndrome; nt, not tested; PV, polycytemia vera; wt, wild type.

Table S2. Clinical and biological characteristics of patients showing a strong antiproliferative effect of V-ATPase inhibition (bafilomycin 10 nM). The table presents the data for all 37 patients included in the study, showing detectable cytokine-dependent proliferation (70 patients) and showing a relative response (proliferation in bafilomycin cultures relative to medium cultures) ≤0.30 (37 of the 70 patients). The patients are listed based on the antiproliferative effect, i.e. the relative response in decreasing order. The ^{3}H-thymidine incorporation in cultures containing only dead cells corresponded to <150 cpm, and the background count for wells prepared with medium alone without cells was <100 cpm. Patients with MDS-associated mutations are marked with green.

Id	Relative response BAF 10 nM	Proliferation (cpm) BAF10nM	Proliferation (cpm) Controls	Sex	Age	Secondary/relapsed AML	Karyotype	FLT3 mutations	NPM-1 mutation	FAB classification	CD34 expression	PI3K-Akt-mTOR activity
7	0.30	4620	15.164	M	78	Secondary AML (CMML)	+8	nt	nt	M4	-	High
32	0.30	1995	6590	M	60	Secondary AML (MDS)	Normal	nt	nt	M2	+	nt
19	0,29	8822	30.314	F	86		nt	nt	nt	M2	+	nt
52	0,29	431	1459	F	92	Secondary AML (chemotherapy)	nt	nt	nt	M1	-	nt
8	0,24	826	3472	F	49	Relapse	Complex	nt	nt	M2	+	High
58	0,24	2534	102.303	F	29		Normal	ITD+Asp835	wt	M5	+	nt
69	0,23	1898	8155	F	55		Normal	ITD	INS	M1	+	High
66	0,21	2717	12.919	M	72	Secondary AML (MDS)	Normal	nt	nt	M4	-	Low
41	0,20	1453	7231	M	41		Normal	wt	wt	M4	nt	nt
2	0,19	15.709	80.671	M	81	Secondary AML (PV)	-7	wt	wt	M2	-	Low
18	0,19	31.547	162.418	F	71	Secondary AML (MDS)	del(12)	nt	nt	nt	+	Low
78	0,18	1780	9839	F	77		nt	wt	INS	M1	-	High
73	0,17	13.820	81.412	M	62		+8	wt	wt	M4	+	nt
25	0,16	372	2374	M	71		nt	nt	nt	nt	nt	nt
27	0,16	1318	8350	M	65	Secondary AML (MDS)	Normal	wt	INS	M1	-	nt
51	0,16	1504	9257	M	36		inv16 (p13,1q22)	wt	wt	M4	+	Low
31	0,15	6685	43.862	F	67		+21	wt	wt	M0	+	nt
60	0,14	24.287	173.197	M	76	Secondary AML (CMML)	del12, -7	nt	nt	M5	+	High
30	0,12	2357	23.879	F	78		Normal	ITD	INS	M1	-	High
6	0,11	681	6042	M	33		Normal	ITD	INS	M4	+	nt
36	0,11	10.368	97.008	M	35		Normal	wt	wt	M2	+	Low
62	0,11	221	1929	M	78		Complex	wt	wt	M0	-	nt
23	0,10	1222	12.225	F	18		Inv16	wt	wt	M4	+	Low
34	0,10	275	2639	F	59		Normal	ITD	INS	M5	-	Low
39	0,10	218	2117	M	72	Secondary AML (MDS)	Complex	wt	nt	M1	+	Low
22	0,09	210	2409	M	24		Complex	nt	wt	M2	+	High
74	0,09	423	4584	F	87		del5 (q13q33)	wt	wt	M0	+	High
38	0,08	1273	2128	F	82		Normal	ITD	wt	M4	+	Low
72	0,07	699	9407	M	76		Normal	wt	wt	M0	+	Low
75	0,07	530	7344	M	59	Secondary AML (CMML)	del20, +8	ITD	wt	M5	+	nt

40	0,05	919	18.044	F	71	Secondary AML (MDS)	t(1;5), t(2;3)	nt	nt	nt	+	Low
37	0,03	3807	10.901	M	76	Secondary AML (MDS)	Normal	nt	nt	nt	+	High
65	0,03	857	34.137	F	64	Secondary AML (MDS)	42-46, XX, -16,-22 [cp25]	wt	wt	M4	+	nt
79	0,03	1421	52.411	F	50		-7, t(3;3) (q21.3;q26.2)	wt	wt	M2	+	nt
9	0,02	342	22.135	M	32		Normal	wt	wt	M2	+	nt
10	0,02	678	33.452	F	51	Relapse	Complex	wt	wt	M0	+	High
24	0,02	260	11.102	F	77	Secondary AML (MDS)	Normal	ITD	INS	M1/2	nt	Low
13	0,01	5302	53.050	F	77	Secondary AML (MDS)	Normal	wt	wt	M1	+	High

Abbreviations: CMML, chronic myelomonocytic leukemia; MDS, myelodysplastic syndrome; nt, not tested; PV, polycytemia vera; wt, wild type.

Table S3. The results from molecular genetic analysis from 33 AML patients. We investigated a panel of 57 mutations and the table presents the results for those 32 mutations that could be detected among these patients. Patients with MDS-associated mutations are marked with green (PMID 35797463) (Tumor suppr, tumor suppressors; TF, transcription factors). Patients showing a strong antiproliferative effect of bafilomycin 1A 10 nM are marked with *.

Patient		Signaling							Tumor suppr.				DNA Methylation				Chromatin			TFs		Spliceosome						Cohesin			Others	
	NPM1	*FLT3-ITD*	*FLT3-TKD*	*HRAS*	*KIT*	*KRAS*	*NRAS*	*PTPN11*	*IKZF1*	*PHF6*	*TP53*	*WT1*	*DNMT3A*	*TET2*	*IDH1*	*IDH2*	*ASXL1*	*EZH2*	*GATA2*	*CEBPA*	*RUNX1*	*BCOR*	*BCORL1*	*CSF3R*	*SF3B1*	*SRSF2*	*ZRSR2*	*RAD21*	*SMC1A*	*STAG2*	*SETBP1*	*CDKN2A*
1		■																		■												
2*																	■									■		■				
4														■			■			■						■				■		
10*											■																					
11		■												■			■										■			■		
12															■		■															
13*							■							■			■				■	■				■				■		
14	■						■						■																			
15	■						■						■			■																
16						■												■											■		■	
18*						■	■	■									■													■		
19*								■									■				■					■						
23*	■				■							■																				
24*	■	■											■												■							
25*	■		■													■										■						
26							■																									
30*	■	■											■																			
31*																				■												
33		■							■				■																			■
36*				■					■					■					■	■	■											
38*		■	■									■	■																			
39*		■	■										■								■											
41*																	■				■		■									
51*							■																									
53	■	■											■	■																		
55																																
58*			■									■									■											
59			■							■					■															■		
65*							■																		■							
70		■					■																									
71	■												■											■								
72*																■																
73*														■			■	■		■	■							■				

Table S4. The results from molecular genetic analyses of primary AML cells derived from 33 AML patients. We investigated a panel of 57 mutations and the table presents the results for those MDS-associated mutations (PMID 35797463) that could be detected among these patients. Patient id is presented in the left column, and the 17 patients with MDS-associated mutations are marked with green. The relative antiproliferative effect of bafilomycin A1 10 nM is indicated in this right column, and a strong antiproliferative effect is indicated by a green color in the column. Patients with MDS associated abnormalities showed a higher frequency of strong responders to bafilomycin than the other 16 patients (Fisher's test, p=0.0799).

	ASXL1	*EZH2*	*RUNX1*	*BCOR*	*SF3B1*	*SRSF2*	*ZRSR2*	*STAG2*	
2									0.19
4									0.52
11									0.39
12									0.09
13									0.01
16									0.90
18									0.19
19									0.29
24									0.02
25									0.16
36									0.11
39									0.05
41									0.20
58									0.24
59									0.59
65									0.03
73									0.17
1									0.37
10									0.19
14									0.89
15									0,71
23									0.10
26									0.38
30									0.12
31									0.15
33									0.33
38									0.08
51									0.16
53									0.72
55									0.57
70									0.43
71									0.61
72									0.07

Table S5. Differentially expressed proteins when comparing AML cells showing strong (eight patients, relative response ≤0.30) and weak (seven patients, relative response ≥0.60) antiproliferative effect of the V-ATPase inhibitor bafilomycin A1 10 nM. The proteins were identified by fulfilling the following criteria: (i) a significant difference when using a t-test to analyze the expression levels between the two contrasting patient groups (the corresponding p-value is presented); (ii) a statistically significant fold change (FC; the corresponding z score is indicated); and reaching detectable levels for at least five patients in each group. The proteins are listed according to their FC value, i.e., the value for strong responders relative to the value for weak responders. The table presents the protein identity, the protein name, the corresponding p-value for the protein level comparison (t-test), and the FC between the means for patients showing a strong versus a weak antiproliferative effect of bafilomycin A1e. Proteins showing increased levels for AML cells with a strong antiproliferative effect are indicated by green color, proteins showing a high level in AML cells with a weak antiproliferative effect are indicated by blue color.

Identity	Name	p-value t test	Fold change STRONG/WEAK	z-value FC
APOB	Apolipoprotein B-100;Apolipoprotein B-48	0,03369306	3,3614	0
ALDH2	Aldehyde dehydrogenase, mitochondrial	0,00563231	3,0260	0
LTF	Lactotransferrin;Lactoferricin-H;Kaliocin-1;Lactoferroxin-A;Lactoferroxin-B;Lactoferroxin-C	0,04805837	2,3385	1,7653E-14
SERPINA3	Alpha-1-antichymotrypsin;Alpha-1-antichymotrypsin His-Pro-less	0,02803989	2,1620	1,3769E-12
CLEC11A	C-type lectin domain family 11 member A	0,02377505	2,0517	1,7663E-11
ANXA3	Annexin A3	0,02359296	1,9522	1,5728E-10
CHI3L1	Chitinase-3-like protein 1	0,01003779	1,9006	4,6944E-10
TFRC	Transferrin receptor protein 1;Transferrin receptor protein 1, serum form	0,00781391	1,6989	2,5442E-08
FN1	Fibronectin;Anastellin;Ugl-Y1;Ugl-Y2;Ugl-Y3	0,02396454	1,5495	3,6939E-07
SERPINB9	Serpin B9	0,02571954	1,4178	3,2046E-06
SLC29A1	Equilibrative nucleoside transporter 1	0,03325502	1,4119	3,516E-06
AKR1C3	Aldo-keto reductase family 1 member C3	0,00995353	1,3868	5,1903E-06
IFITM1;IFITM2;IFITM3	Interferon-induced transmembrane protein 1;Interferon-induced transmembrane protein 2;Interferon-induced transmembrane protein 3	0,01477258	1,3255	1,304E-05
CPNE3	Copine-3	0,03230634	1,2459	4,0726E-05
HLA-DPA1	HLA class II histocompatibility antigen, DP alpha 1 chain	0,01382042	1,1867	9,0838E-05
SNX20	Sorting nexin-20	0,01517024	1,1767	0,00010375
SLC7A5	Large neutral amino acids transporter small subunit 1	0,02468821	1,1063	0,00025506
RBM34	RNA-binding protein 34	0,0398698	1,1006	0,00027365
ASS1	Argininosuccinate synthase	0,01791451	1,0951	0,00029275
		0,02751016	1,0950	0,00029318
IGFBP2	Insulin-like growth factor-binding protein 2	0,02895331	1,0030	0,00086895
CST7	Cystatin-F	0,0377201	0,9841	0,00107481
TOP2A	DNA topoisomerase 2-alpha	0,01263248	0,9632	0,00135328
STOM	Erythrocyte band 7 integral membrane protein	0,02525846	0,9592	0,0014137
SLC1A5	Neutral amino acid transporter B(0)	0,017686	0,8944	0,00280314
PLSCR1	Phospholipid scramblase 1	0,04158713	0,8718	0,00351925
APMAP	Adipocyte plasma membrane-associated protein	0,02530763	0,8715	0,00353019
HK2	Hexokinase-2	0,04768652	0,7857	0,008018
IGKC	Ig kappa chain C region	0,03154742	0,7555	0,01050468
MIA3	Melanoma inhibitory activity protein 3	0,03580034	0,7033	0,016428
BAZ1A	Bromodomain adjacent to zinc finger domain protein 1A	0,01841654	0,7028	0,01649612
NOB1	RNA-binding protein NOB1	0,01808787	0,6718	0,02122889
CFAP99	Cilia- and flagella-associated protein 99	0,04821181	0,6679	0,02190257
DDX55	ATP-dependent RNA helicase DDX55	0,01010196	0,6607	0,02317923
HDAC6	Histone deacetylase 6	0,04962441	0,6362	0,02802034
ANXA11	Annexin A11	0,01904658	0,6188	0,03195898
RPL36A	60S ribosomal protein L36a	0,01729885	0,5800	0,04239539

HIST1H2AJ; HIST1H2AH; HIST1H2AD; HIST1H2AG; H2AFJ	Histone H2A type 1-J;Histone H2A type 1-H;Histone H2A type 1-D;Histone H2A type 1;Histone H2A.J	0,02034757	0,5679	0,0461572
TM9SF2	Transmembrane 9 superfamily member 2	0,02499579	0,5642	0,04736818
SRI	Sorcin	0,04087411	0,5594	0,04897732
MED20	Mediator of RNA polymerase II transcription subunit 20	0,03125701	-0,4380	0,04937587
ARFGAP1	ADP-ribosylation factor GTPase-activating protein 1	0,03952714	-0,4701	0,03948502
TRPV2	Transient receptor potential cation channel subfamily V member 2	0,02896324	-0,4749	0,03815388
ARHGAP15	Rho GTPase-activating protein 15	0,03885734	-0,4839	0,03575859
BICD2	Protein bicaudal D homolog 2	0,03142263	-0,4879	0,03473463
RAP1GDS1	Rap1 GTPase-GDP dissociation stimulator 1	0,04518305	-0,5451	0,02251536
PPA1	Inorganic pyrophosphatase	0,01849544	-0,5963	0,01487974
CDK5	Cyclin-dependent-like kinase 5	0,02107095	-0,6375	0,01046886
NLN	Neurolysin, mitochondrial	0,02861415	-0,6463	0,00968454
ITPA	Inosine triphosphate pyrophosphatase	0,03639413	-0,6555	0,00892475
MAP4	Microtubule-associated protein 4	0,03755862	-0,6872	0,00669273
WDR44	WD repeat-containing protein 44	0,02118769	-0,7069	0,00557244
SCYL2	SCY1-like protein 2	0,04733707	-0,7409	0,00401972
PDLIM5	PDZ and LIM domain protein 5	0,01764782	-0,9958	0,00024002
TNS3	Tensin-3	0,02667578	-1,0009	0,00022544
TAGLN2	Transgelin-2	0,03806605	-1,0243	0,00016815
LMNA	Prelamin-A/C;Lamin-A/C	0,04129316	-1,0983	6,4145E-05
ARL3	ADP-ribosylation factor-like protein 3	0,00726666	-1,1364	3,8177E-05
ABHD11	Alpha/beta hydrolase domain-containing protein 11	0,04435703	-1,1717	2,3293E-05
DMXL2	DmX-like protein 2	0,00828619	-1,4240	4,6817E-07
TUBB6	Tubulin beta-6 chain	0,01114038	-1,5831	2,8219E-08

Table S6. Unsupervised hierarchical clustering of differentially expressed proteins when comparing AML patient cells showing string versus weak antiproliferative effects of V-ATPase inhibition. The table lists the differentially expressed proteins (identified by the gene names) from the top and downwards for the heatmap presented in Figure 2A.

**UPPER MAIN CLUSTER OF FIGURE 2A** Proteins showing significantly increased levels in AML cells with a weak antiproliferative effect of V-ATPase inhibition (21 proteins)	**LOWER MAIN CLUSTER OF FIGURE 2A** Proteins showing significantly increased levels in AML cells with a strong antiproliferative effect of V-ATPase inhibition (40 proteins)
NLN	CLEC11A
ABHD11	MIA3
DMXL2	SERPINB9
ARL3	NOB1
SCYL2	DDX55
PDLIM5	SNX20
MED20	AKR1C3
TRPV2	APMAP
TUBB6	LTF
LMNA	SLC29A1
ARFGAP1	RPL36A
TNS3	TOP2A
BICD2	ALDH2
PPA1	HK2
ITPA	SERPINA3
RAP1GDS1	HLA-DPA1
WDR44	SLC1A5
TAGLN2	CST7
MAP4	TM9SF2
ARHGAP15	ANXA11
CDK5	CPNE3
	PLSCR1
	SLC7A5
	ASS1
	RBM34
	HDAC6
	BAZ1A
	SRI
	IGFBP2
	HIST1H2AJ
	TTR
	FN1
	IFITM1
	STOM
	ANXA3
	TFRC
	APOB
	IGKC
	CHI3L1
	CFAP99

Table S7. Differentially expressed proteins when comparing primary AML cells showing a weak and a strong antiproliferative effect of V-ATPase inhibition (bafilomycin A1 10 nM). The information is based on the Gene database (accessed 280323) and selected references from the PubMed database (accessed March 28 2023), and the table includes the proteins that were identified both by the statistical comparison, the volcano analyses and the protein-protein interaction analyses. The table presents the differentially expressed protein/phosphosite identity, the altered residues (only for the phosphoproteomic comparisons) and the ratio between the medians for patients/cells showing a strong versus a weak antiproliferative effect of bafilomycin (left column), a description of the protein function (middle column) and key words with regard to the molecular function (right). These proteins and phosphosites showed differential expression and phosphorylation, respectively, and they were in addition identified in the protein-protein interaction analyses and the volcano plot analyses.

	**PROTEOMIC COMPARISON**	
**Protein id**	**Biological characterization**	**Key words**
ALD2<br>3.026	*Aldolase 2.* The protein is possibly involved in fructose 1,6-bisphosphate metabolic process and glycolytic process.	Metabolism?
ANXA3<br>1.9522	*Annexin A3.* This protein is a member of the annexin family. Members of this calcium-dependent phospholipid-binding protein family play a role in the regulation of cellular growth and in signal transduction pathways. This protein functions in the inhibition of phospholipase A2 and cleavage of inositol 1,2-cyclic phosphate to form inositol 1-phosphate. The ANXA3 level is increased especially in AML-M2 leukemic cells (Handschuh 2018), and the AML cell level seems to be associated with prognosis after intensive chemotherapy (Chen T 2022).	Proliferation<br>Signal transduction<br>AML prognosis
ANXA11<br>0.6188	*Annexin A11.* This protein is a member of the annexin family, a group of calcium-dependent phospholipid-binding proteins. Annexins have unique N-terminal domains and conserved C-terminal domains, which contain calcium-dependent phospholipid-binding sites.	Proliferation<br>Signal transduction
APOB<br>3,3614	*Apolipoprotein B.* This gene product is the main apolipoprotein of chylomicrons and low density lipoproteins (LDL), and is the ligand for the LDL receptor. High expression of this gene is seen in AML cells with Flt3-ITD (Chen S 2022).	Lipid metabolism
CHI3L1<br>1.9006	*Chitinase 3 like 1 (also called YKL-40).* The glycoside hydrolase 18 family of chitinases includes eight human family members. This gene encodes a glycoprotein member of the glycosyl hydrolase 18 family. The protein is secreted by myeloid cells, including activated macrophages and neutrophils, but also chondrocytes and synovial cells. The protein is thought to play a role in inflammation and tissue remodeling. High AML cell expression of this gene is a part of a chemoresistant phenotype, and high systemic/serum levels in untreated AML are also associated with decreased long-term survival (Chen 2020, Bergmann 2005). Regulation of iron metabolism seems to have a prognostic impact in human AML (Brenner 2017).	Inflammation<br>AML survival
CLEC11A<br>2.0517	*C-type lectin domain containing 11A.* This gene encodes a member of the C-type lectin superfamily. This protein is a secreted sulfated glycoprotein and functions as a growth factor for primitive hematopoietic progenitor cells. High AML cell levels are associated with a favorable prognosis (Zhang 2009, Yin 2021).	Lectin<br>Secreted<br>Favorable AML
FN1<br>1.5495	*Fibronectin 1.* This glycoprotein is present in a soluble dimeric form in plasma, and in a dimeric or multimeric form at the cell surface and in extracellular matrix. The encoded preproprotein is proteolytically processed to generate the mature protein. Fibronectin is involved in cell adhesion and migration, wound healing, blood coagulation, host defense, and metastasis. The gene has three regions subject to alternative splicing, with the potential to produce 20 different transcript variants. Experimental studies suggest that VLA-4-positive AML cells can acquire chemoresistance by the interaction of VLA-4 and fibronectin; this is possibly mediated through the phosphatidylinositol-3-kinase (PI-3K)/AKT/Bcl-2 signaling pathway (Becker 2009, Kuzelova 2020, Matsunags 2003).	Cell adhesion<br>Migration<br>Extracellular matrix<br>AML chemoresistance
SERPINA3<br>1.4178	*Serpin family A member 3.* The encoded protein is a member of the serpin family of proteins, a group that inhibit serine proteases. This gene is one in a cluster of serpin genes located on the q arm of chromosome 14. Polymorphisms in this protein appear to influence protease targeting.	Serine protease inhibitor
TFRC<br>1.6989	*Transferrin receptor.* This gene encodes a cell surface receptor necessary for cellular iron uptake by the process of receptor-mediated endocytosis. Multiple alternatively spliced variants have been identified. V-ATPase is involved in the regulation of iron metabolism, modulation of iron metabolism is important for the anticancer effect of V-ATPase inhibitors, and chemosensitive and chemoresistant AML cells seem to differ with regard to regulation of iron metabolism (Brenner 2017, Schneider 2015, Straud 2010).	Iron uptake<br>Endosome<br>Anticancer effect<br>AML prognosis

CDK5<br>-0.6375	*Cyclin dependent kinase 5.* This gene encodes a proline-directed serine/threonine kinase that is a member of the cyclin-dependent kinase family. This protein does not directly control cell cycle regulation, instead the protein functions in diverse processes through phosphorylation of proteins required for cytoskeletal organization, endocytosis and exocytosis, and apoptosis. Experimental AML studies suggest that this protein has a proapoptotic effect, and increased levels are associated with monocytic differentiation (Chen F 2000, Sandal 2002)	Cytoskeleton<br>Endo-/exocytosis<br>Apoptosis
LMNA<br>-1.0983	*Lamin A/C.* The protein is part of the nuclear lamina, a two-dimensional matrix of proteins located next to the inner nuclear membrane. During mitosis, the lamina matrix is reversibly disassembled as the lamin proteins are phosphorylated. Lamin proteins are thought to be involved in nuclear stability, chromatin structure and gene expression.	Nuclear lamina Mitosis<br>Chromatin structure
TUBB6<br>-1.5831	*Tubulin beta 6 class V.* Predicted to enable GTP binding activity and to be a structural constituent of cytoskeleton. It is predicted to be involved in microtubule cytoskeleton organization and mitotic cell cycle. The protein is located in microtubule.	Cytoskeleton<br>Mitosis
	**PHOSPHOPROTEOMIC COMPARISON**	
CBX3<br>S93<br>1.3063	*Chromobox 3.* The nuclear lamina and heterochromatin are adjacent to the inner nuclear membrane, and the encoded protein binds DNA and is a component of heterochromatin. It can also bind the lamine B receptor, an integral membrane protein found in the inner nuclear membrane. The dual binding functions of the protein may explain the association of heterochromatin with the inner nuclear membrane. This protein binds histone H3 tails methylated at Lys-9 sites.	Nuclear membrane<br>Chromatin<br>Histones
CDK12<br>T893<br>0.7160	*Cyclin dependent kinase 12.* The protein has RNA polymerase II CTD heptapeptide repeat kinase activity and cyclin binding activity. It is involved in phosphorylation of RNA polymerase II C-terminal domain and regulation of MAP kinase activity. The protein is located in nuclear speck, is a part of the cyclin K-CDK12 complex. Inhibition of CDK12 may have an inhibitory effect on AML stem cells (He 2022).	RNA<br>MAP kinase activity<br>Nuclear protein<br>AML stem cells
CCNY<br>S326<br>0.8762	*Cyclin Y.* Cyclins, such as CCNY, control cell division cycles and regulate cyclin-dependent kinases.	CDK regulation<br>Mitosis
FLNA<br>S2150<br>2.1794	*Filamin A.* This is an actin-binding protein that crosslinks actin filaments and links actin filaments to membrane glycoproteins. It is involved in remodeling the cytoskeleton to effect changes in cell shape and migration. This protein interacts with integrins, transmembrane receptor complexes, and second messengers. The gene is a fusion partner in AML-associated translocations (Lentes 2016, Matveeva 2015).	Actin<br>Cytoskeleton<br>Cell migration<br>AML
HIST1H1E<br>S2,T18<br>2.419	*H1.4 linker histone, cluster member.* Two molecules of each of the four core histones (H2A, H2B, H3, and H4) form an octamer, around which approximately 146 bp of DNA is wrapped in repeating units, called nucleosomes. The H1 histone linker interacts with DNA between nucleosomes and functions in the compaction of chromatin. Low mRNA levels are associated with adverse prognosis in human AML, this is possibly caused by crosstalk with histone methylation and/or the effect of these epigenetic modulations on metabolic regulation (Chang 2019, Ye 2019).	Histone linker<br>Adverse prognosis
JUNB<br>T255,S259<br>1.868	*JunB proto-oncogene, AP-1 transcription factor subunit.* The protein has sequence-specific double-stranded DNA binding activity and is involved in regulation of transcription by RNA polymerase II. It is located in nucleoplasm and is a part of the transcription factor AP-1 complex. JunB suppresses the formation of AML stem cells (Sommervaille 2006, Steidl 2006)	Transcription<br>AML stem cells
RP1<br>S38<br>1.9893	*RP1 axonemal microtubule associated.* This gene encodes a member of the doublecortin family. The protein binds microtubules and regulates microtubule polymerization.	Microtubules
RPL12<br>S38<br>1.9890	*Ribosomal protein L12.* Ribosomes consist of a small 40S subunit and a large 60S subunit. Together these subunits are composed of 4 RNA species and approximately 80 structurally distinct proteins. This gene encodes a ribosomal protein that is a component of the 60S subunit. The protein belongs to the L11P family of ribosomal proteins. It is located in the cytoplasm. The protein binds directly to the 26S rRNA.	Ribosome<br>Cytoplasm
SERBP1<br>S330<br>0.6736	*SERPINE1 mRNA binding protein 1.* The protein enables SUMO, mRNA 3'-UTR and ribosome binding activities. It is involved in PML body organization. The protein seems to be involved in chromatin modulation and is located in cytosol, perinuclear area and nucleus (Lemos).	Ribosom<br>Chromatin<br>Cytoplasm, nucleus

AHNAK<br>S210,<br>S5731<br>-0.9083	*AHNAK nucleoprotein.* The protein is a large (700 kDa) structural scaffold protein. It may play a role in cell migration, calcium channel regulation, and tumor metastasis.	Cell migration
CHAMP1<br>S432<br>-0.6928	*Chromosome alignment maintaining phosphoprotein 1.* This gene encodes a zinc finger protein that functions as a regulator of chromosome segregation in mitosis. The encoded protein is required for correct alignment of chromosomes on the metaphase plate, and plays a role in maintaining the attachment of sister kinetochores to microtubules from opposite spindle poles.	Chromosome segregation<br>Microtubules

References to the table

Becker PS, Kopecky KJ, Wilks AN, Chien S, Harlan JM, Willman CL, Petersdorf SH, Stirewalt DL, Papayannopoulou T, Appelbaum FR. Very late antigen-4 function of myeloblasts correlates with improved overall survival for patients with acute myeloid leukemia. Blood. 2009 Jan 22;113(4):866-74. doi: 10.1182/blood-2007-12-124818. Epub 2008 Oct 16. PMID: 18927435; PMCID: PMC2630271.

Bergmann OJ, Johansen JS, Klausen TW, Mylin AK, Kristensen JS, Kjeldsen E, Johnsen HE. High serum concentration of YKL-40 is associated with short survival in patients with acute myeloid leukemia. Clin Cancer Res. 2005 Dec 15;11(24 Pt 1):8644-52. doi: 10.1158/1078-0432.CCR-05-1317. PMID: 16361549.

Brenner AK, Tvedt TH, Nepstad I, Rye KP, Hagen KM, Reikvam H, Bruserud Ø. Patients with acute myeloid leukemia can be subclassified based on the constitutive cytokine release of the leukemic cells; the possible clinical relevance and the importance of cellular iron metabolism. Expert Opin Ther Targets. 2017 Apr;21(4):357-369. doi: 10.1080/14728222.2017.1300255. Epub 2017 Mar 3. PMID: 28281897.

Chang S, Yim S, Park H. The cancer driver genes IDH1/2, JARID1C/ KDM5C, and UTX/ KDM6A: crosstalk between histone demethylation and hypoxic reprogramming in cancer metabolism. Exp Mol Med. 2019 Jun 20;51(6):1-17. doi: 10.1038/s12276-019-0230-6. PMID: 31221981; PMCID: PMC6586683.

Chen F, Rao J, Studzinski GP. Specific association of increased cyclin-dependent kinase 5 expression with monocytic lineage of differentiation of human leukemia HL60 cells. J Leukoc Biol. 2000 Apr;67(4):559-66. doi: 10.1002/jlb.67.4.559. PMID: 10770290.

Chen S, Chen Y, Zhu Z, Tan H, Lu J, Qin P, Xu L. Identification of the key genes and microRNAs in adult acute myeloid leukemia with FLT3 mutation by bioinformatics analysis. Int J Med Sci. 2020 May 18;17(9):1269-1280. doi: 10.7150/ijms.46441. PMID: 32547322; PMCID: PMC7294926.

Chen T, Zhang J, Wang Y, Zhou H. Identification of Survival-Related Genes in Acute Myeloid Leukemia (AML) Based on Cytogenetically Normal AML Samples Using Weighted Gene Coexpression Network Analysis. Dis Markers. 2022 Sep 29;2022:5423694. doi: 10.1155/2022/5423694. PMID: 36212177; PMCID: PMC9537620.

Handschuh L, Kaźmierczak M, Milewski MC, Góralski M, Łuczak M, Wojtaszewska M, Uszczyńska-Ratajczak B, Lewandowski K, Komarnicki M, Figlerowicz M. Gene expression profiling of acute myeloid leukemia samples from adult patients with AML-M1 and -M2 through boutique microarrays, real-time PCR and droplet digital PCR. Int J Oncol. 2018 Mar;52(3):656-678. doi: 10.3892/ijo.2017.4233. Epub 2017 Dec 28. PMID: 29286103; PMCID: PMC5807040.

He L, Arnold C, Thoma J, Rohde C, Kholmatov M, Garg S, Hsiao CC, Viol L, Zhang K, Sun R, Schmidt C, Janssen M, MacRae T, Huber K, Thiede C, Hébert J, Sauvageau G, Spratte J, Fluhr H, Aust G, Müller-Tidow C, Niehrs C, Pereira G, Hamann J, Tanaka M, Zaugg JB, Pabst C. CDK7/12/13 inhibition targets an oscillating leukemia stem cell network and synergizes with venetoclax in acute myeloid leukemia. EMBO Mol Med. 2022 Apr 7;14(4):e14990. doi: 10.15252/emmm.202114990. Epub 2022 Mar 7. PMID: 35253392; PMCID: PMC8988201.

Kuželová K, Obr A, Marková J, Gašová Z. Integrin expression and adhesivity to fibronectin in primary acute myeloid leukemia cells: Impact of NPM1 and FLT3 mutations. Eur J Haematol. 2020 Nov;105(5):578-587. doi: 10.1111/ejh.13488. Epub 2020 Aug 11. PMID: 32668024.

Lemos TA, Passos DO, Nery FC, Kobarg J. Characterization of a new family of proteins that interact with the C-terminal region of the chromatin-remodeling factor CHD-3. FEBS Lett. 2003 Jan 2;533(1-3):14-20. doi: 10.1016/s0014-5793(02)03737-7. PMID: 12505151.

Lentes J, Thomay K, Schneider DT, Bernbeck B, Reinhardt D, Marschalek R, Meyer C, Schlegelberger B, Göhring G. Identification of a Cryptic Insertion ins(11;X)(q23;q28q12) Resulting in a KMT2A-FLNA Fusion in a 13-Month-Old Child with Acute Myelomonocytic Leukemia. Cytogenet Genome Res. 2016;150(3-4):281-286. doi: 10.1159/000458165. Epub 2017 Mar 3. PMID: 28253492.

Matveeva E, Kazakova A, Olshanskaya Y, Tsaur G, Shelikhova L, Meyer C, Marschalek R, Novichkova G, Maschan M, Maschan A. A new variant of KMT2A(MLL)-FLNA fusion transcript in acute myeloid leukemia with ins(X;11)(q28;q23q23). Cancer Genet. 2015 Apr;208(4):148-51. doi: 10.1016/j.cancergen.2015.03.001. Epub 2015 Mar 7. PMID: 25892123.

Matsunaga T, Takemoto N, Sato T, Takimoto R, Tanaka I, Fujimi A, Akiyama T, Kuroda H, Kawano Y, Kobune M, Kato J, Hirayama Y, Sakamaki S, Kohda K, Miyake K, Niitsu Y. Interaction between leukemic-cell VLA-4 and stromal fibronectin is a decisive factor for minimal residual disease of acute myelogenous leukemia. Nat Med. 2003 Sep;9(9):1158-65. doi: 10.1038/nm909. Epub 2003 Aug 3. Erratum in: Nat Med. 2005 May;11(5):578. PMID: 12897778.

Sandal T, Stapnes C, Kleivdal H, Hedin L, Døskeland SO. A novel, extraneuronal role for cyclin-dependent protein kinase 5 (CDK5): modulation of cAMP-induced apoptosis in rat leukemia cells. J Biol Chem. 2002 Jun 7;277(23):20783-93. doi: 10.1074/jbc.M112248200. Epub 2002 Mar 21. PMID: 11909854.

Schneider LS, von Schwarzenberg K, Lehr T, Ulrich M, Kubisch-Dohmen R, Liebl J, Trauner D, Menche D, Vollmar AM. Vacuolar-ATPase Inhibition Blocks Iron Metabolism to Mediate Therapeutic Effects in Breast Cancer. Cancer Res. 2015 Jul 15;75(14):2863-74. doi: 10.1158/0008-5472.CAN-14-2097. Epub 2015 May 27. PMID: 26018087.

Somervaille TC, Cleary ML. PU.1 and Junb: suppressing the formation of acute myeloid leukemia stem cells. Cancer Cell. 2006 Dec;10(6):456-7. doi: 10.1016/j.ccr.2006.11.009. PMID: 17157786.

Steidl U, Rosenbauer F, Verhaak RG, Gu X, Ebralidze A, Otu HH, Klippel S, Steidl C, Bruns I, Costa DB, Wagner K, Aivado M, Kobbe G, Valk PJ, Passegué E, Libermann TA, Delwel R, Tenen DG. Essential role of Jun family transcription factors in PU.1 knockdown-induced leukemic stem cells. Nat Genet. 2006 Nov;38(11):1269-77. doi: 10.1038/ng1898. Epub 2006 Oct 15. PMID: 17041602.

Straud S, Zubovych I, De Brabander JK, Roth MG. Inhibition of iron uptake is responsible for differential sensitivity to V-ATPase inhibitors in several cancer cell lines. PLoS One. 2010 Jul 16;5(7):e11629. doi: 10.1371/journal.pone.0011629. PMID: 20661293; PMCID: PMC2905441.

Ye C, Ma S, Xia B, Zheng C. Weighted Gene Coexpression Network Analysis Identifies Cysteine-Rich Intestinal Protein 1 (CRIP1) as a Prognostic Gene Associated with Relapse in Patients with Acute Myeloid Leukemia. Med Sci Monit. 2019 Oct 2;25:7396-7406. doi: 10.12659/MSM.918092. PMID: 31577790; PMCID: PMC6790098.

Yin C, Zhang J, Guan W, Dou L, Liu Y, Shen M, Jia X, Xu L, Wu R, Li Y. High Expression of CLEC11A Predicts Favorable Prognosis in Acute Myeloid Leukemia. Front Oncol. 2021 Mar 2;11:608932. doi: 10.3389/fonc.2021.608932. PMID: 33747924; PMCID: PMC7966831.

Zhang Y, Xiao L. Identification and validation of a prognostic 8-gene signature for acute myeloid leukemia. Leuk Lymphoma. 2020 Aug;61(8):1981-1988. doi: 10.1080/10428194.2020.1742898. Epub 2020 Apr 8. PMID: 32268820.

Table S8. Differentially expressed protein phosphorylation sites when comparing AML cells showing a weak (seven patients, relative response ≥0.60) and strong (eight patients, relative response ≤0.30) antiproliferative effect of the V-ATPase inhibitor bafilomycin A1 10 nM. The phosphosites were identified by fulfilling two criteria: (i) a significant difference when using a t-test to analyze the phosphorylation levels between the two contrasting patient groups (the corresponding p-value is presented); and (ii) a statistically significant FC (the corresponding z score is indicated). The phosphosites are listed according to their fold change value, i.e., the value for strong responders relative to the value for weak responders. The table presents the protein identity (Id), protein name, altered phosphorylation site (amino acid and residue) with the corresponding p-value for the phosphorylation level (t test comparison), and the FC between the means for patients showing a strong versus a weak antiproliferative effect of bafilomycin A1 10 nM. Proteins showing increased phosphorylation levels for AML cells with a strong antiproliferative effect are indicated by green color, proteins showing high phosphorylation levels in AML cells with a weak antiproliferative effect are indicated by blue color.

Id	Name	AA	Residue	p-value t test	FC strong vs. weak	z-value FC
HIST1H1E	Histone H1.4	T	18	0,00356455	2,4119	1,7238E-12
FLNA	Filamin-A	S	2150	0,01287898	2,1794	1,8977E-10
RPL12	60S ribosomal protein L12	S	38	0,02779176	1,9893	6,2331E-09
JUNB	Transcription factor jun-B	T	255	0,03540563	1,8680	4,8998E-08
HIST1H1E	Histone H1.4	S	2	0,02053688	1,5799	3,9492E-06
JUNB	Transcription factor jun-B	S	259	0,04280414	1,5200	8,9857E-06
HIST1H1E	Histone H1.4	T	18	0,01987535	1,4708	1,7255E-05
LAT2	Linker for activation of T-cells family member 2	S	102	0,0376035	1,3410	8,7377E-05
UNCX	Homeobox protein unc-4 homolog	S	443	0,03307762	1,3207	0,00011124
UNCX	Homeobox protein unc-4 homolog	S	449	0,03307762	1,3207	0,00011124
CBX3	Chromobox protein homolog 3	S	93	0,04451124	1,3063	0,00013159
SURF6	Surfeit locus protein 6	S	138	0,04575705	1,2432	0,00026977
MIA2; CTAGE1	Melanoma inhibitory activity protein 2;cTAGE family member 2	S	1243	0,00345716	1,2076	0,0003988
LSM14A	Protein LSM14 homolog A	S	182	0,01259774	1,2073	0,00040001
LSM14A	Protein LSM14 homolog A	S	183	0,02050757	1,2073	0,00040001
SLC38A1	Sodium-coupled neutral amino acid transporter 1	S	52	0,04769809	1,1721	0,00058223
SLC20A1	Sodium-dependent phosphate transporter 1	S	265	0,04785245	1,1577	0,00067681
SLC20A1	Sodium-dependent phosphate transporter 1	S	269	0,04480222	1,1577	0,00067681
NUMA1	Nuclear mitotic apparatus protein 1	S	1977	0,0028303	1,1502	0,00073161
GATAD2B	Transcriptional repressor p66-beta	S	129	0,0252796	1,0992	0,00122549
DDX54	ATP-dependent RNA helicase DDX54	S	782	0,04685855	1,0898	0,00134579
STMN1	Stathmin	S	63	0,04063644	1,0244	0,00251234
LSP1	Lymphocyte-specific protein 1	T	175	0,00887622	1,0186	0,00265206
PDCD4	Programmed cell death protein 4	S	302	0,03328899	1,0112	0,00283785
SF3B2	Splicing factor 3B subunit 2	S	307	0,04830446	0,9713	0,00406763
HNRNPK	Heterogeneous nuclear ribonucleoprotein K	S	379	0,01385815	0,9586	0,0045503
RLF	Zinc finger protein Rlf	S	634	0,02269552	0,9328	0,00568425
RBM6	RNA-binding protein 6	S	746	0,0075256	0,9115	0,00680332
HIST1H1C	Histone H1.2	S	36	0,01918055	0,9042	0,00723345
SF3B2	Splicing factor 3B subunit 2	S	309	0,0475504	0,9024	0,00733784
CCNY	Cyclin-Y	S	326	0,03434697	0,8762	0,00909311
HIST1H1E; HIST1H1D	Histone H1.4;Histone H1.3	S	36	0,01448219	0,8751	0,00917845
LSP1	Lymphocyte-specific protein 1	S	177	0,01617244	0,8727	0,00935537
TBC1D10C	Carabin	T	415	0,00573438	0,8689	0,00964486
SMARCA2	Probable global transcription activator SNF2L2	S	172	0,03762874	0,8573	0,01057682
SUZ12	Polycomb protein SUZ12	S	546	0,01939481	0,8546	0,01080264

NBAS	Neuroblastoma-amplified sequence	S	473	0,03500025	0,8510	0,01111995
NBAS	Neuroblastoma-amplified sequence	S	475	0,03500025	0,8510	0,01111995
BOD1L1	Biorientation of chromosomes in cell division protein 1-like 1	S	482	0,0385567	0,8317	0,0129263
NFRKB	Nuclear factor related to kappa-B-binding protein	S	298	0,01481046	0,8260	0,01349923
TRA2A	Transformer-2 protein homolog alpha	S	236	0,01910707	0,7855	0,01829778
PATL1	Protein PAT1 homolog 1	S	177	0,01683134	0,7785	0,01926288
CDYL	Chromodomain Y-like protein	S	147	0,01824127	0,7618	0,02171868
RYBP	RING1 and YY1-binding protein	S	123	0,04171127	0,7580	0,02232472
RYBP	RING1 and YY1-binding protein	S	127	0,04171127	0,7580	0,02232472
RYBP	RING1 and YY1-binding protein	S	130	0,04171127	0,7580	0,02232472
SP100	Nuclear autoantigen Sp-100	S	18	0,04355392	0,7426	0,02488413
FBL	rRNA 2'-O-methyltransferase fibrillarin	S	124	0,00544326	0,7414	0,02510096
CDK12	Cyclin-dependent kinase 12	T	893	0,01123924	0,7160	0,02990627
SUPT6H	Transcription elongation factor SPT6	S	1535	0,01708401	0,6992	0,03346758
SUPT6H	Transcription elongation factor SPT6	T	1532	0,01685933	0,6992	0,03346758
SERBP1	Plasminogen activator inhibitor 1 RNA-binding protein	S	330	0,02649759	0,6736	0,03958971
GLYR1	Putative oxidoreductase GLYR1	S	130	0,00415174	0,6703	0,04046286
HNRNPUL1	Heterogeneous nuclear ribonucleoprotein U-like protein 1	S	94	0,04548148	0,6699	0,04056366
ACIN1	Apoptotic chromatin condensation inducer in the nucleus	S	216	0,03973907	0,6680	0,04105238
NUMA1	Nuclear mitotic apparatus protein 1	S	1955	0,00288301	0,6655	0,04170558
ATRIP	ATR-interacting protein	S	391	0,04560225	0,6609	0,04294631
FOXK1	Forkhead box protein K1	S	428	0,01792013	-0,5322	0,04733161
BRAT1	BRCA1-associated ATM activator 1	S	742	0,03442753	-0,5540	0,0417927
BAD	Bcl2-associated agonist of cell death	S	99	0,04573268	-0,5687	0,03836696
FNBP1	Formin-binding protein 1	S	299	0,04439322	-0,5859	0,03465064
GABPA	GA-binding protein alpha chain	S	303	0,04455362	-0,5964	0,03253322
VIM	Vimentin	S	39	0,02334853	-0,6261	0,02712799
CHAMP1	Chromosome alignment-maintaining phosphoprotein 1	S	432	0,03369273	-0,6928	0,01765025
OGFR	Opioid growth factor receptor	S	378	0,02309682	-0,7287	0,01384632
ELAC2	Zinc phosphodiesterase ELAC protein 2	S	199	0,00394969	-0,7303	0,01369184
SIPA1	Signal-induced proliferation-associated protein 1	S	74	0,00972452	-0,7350	0,01325481
UHRF1BP1	UHRF1-binding protein 1	S	1103	0,03639917	-0,7823	0,00947679
UHRF1BP1	UHRF1-binding protein 1	S	1106	0,03639917	-0,7823	0,00947679
RCSD1	CapZ-interacting protein	S	216	0,0445204	-0,7910	0,00889582
HNRNPM	Heterogeneous nuclear ribonucleoprotein M	S	442	0,0457627	-0,8159	0,00740291
RCSD1	CapZ-interacting protein	S	116	0,02824694	-0,8316	0,00657954
RCSD1	CapZ-interacting protein	S	120	0,02472242	-0,8475	0,0058294
FAM21A; FAM21C	WASH complex subunit FAM21A;WASH complex subunit FAM21C	S	284	0,02748412	-0,8568	0,00542407
AHNAK	Neuroblast differentiation-associated protein AHNAK	S	5077	0,02235138	-0,8634	0,00515327
LMNB2	Lamin-B2	S	419	0,04473578	-0,8727	0,0047957
HNRNPM	Heterogeneous nuclear ribonucleoprotein M	S	413	0,0471349	-0,8985	0,00390831
AHNAK	Neuroblast differentiation-associated protein AHNAK	S	5110	0,02648221	-0,9068	0,00365606
ARFGEF2	Brefeldin A-inhibited guanine nucleotide-exchange protein 2	S	227	0,04088744	-0,9474	0,0026196

RCSD1	CapZ-interacting protein	S	120	0,01691161	-0,9871	0,00187154
AHNAK	Neuroblast differentiation-associated protein AHNAK	S	5780	0,02918025	-0,9977	0,00170719
ARFGEF2	Brefeldin A-inhibited guanine nucleotide-exchange protein 2	S	218	0,03733943	-0,9995	0,001681
MAVS	Mitochondrial antiviral-signaling protein	S	222	0,01142101	-1,0010	0,00165899
BIN2	Bridging integrator 2	S	277	0,00751008	-1,0089	0,00154859
RCSD1	CapZ-interacting protein	T	124	0,03476663	-1,0514	0,00106118
EIF4EBP1	Eukaryotic translation initiation factor 4E-binding protein 1	S	65	0,04118706	-1,0676	0,00091585
AHNAK	Neuroblast differentiation-associated protein AHNAK	S	210	0,00355255	-1,0780	0,00083229
ZNF318	Zinc finger protein 318	S	1945	0,02868004	-1,0886	0,00075418
FAM21A; FAM21C	WASH complex subunit FAM21A;WASH complex subunit FAM21C	S	284	0,01449117	-1,1326	0,00049789
PLA2G4A	Cytosolic phospholipase A2;Phospholipase A2;Lysophospholipase	S	729	0,01311363	-1,2380	0,0001743
FAM21A; FAM21C	WASH complex subunit FAM21A;WASH complex subunit FAM21C	S	288	0,00569644	-1,2525	0,00014997
RCSD1	CapZ-interacting protein	S	123	0,04176261	-1,3132	7,87E-05
FAM65B	Protein FAM65B	S	341	0,04239712	-1,3495	5,2849E-05
AHNAK	Neuroblast differentiation-associated protein AHNAK	S	5780	0,00919786	-1,3948	3,1753E-05
TEX2	Testis-expressed sequence 2 protein	S	196	0,00388061	-1,4401	1,8812E-05
BIN2	Bridging integrator 2	S	273	0,00731773	-1,4822	1,1409E-05
AHNAK	Neuroblast differentiation-associated protein AHNAK	S	135	0,01235651	-1,4897	1,0432E-05
RCSD1	CapZ-interacting protein	S	83	0,01288204	-1,4928	1,0045E-05
AHNAK	Neuroblast differentiation-associated protein AHNAK	S	5731	0,03214853	-1,6085	2,3695E-06
AHNAK	Neuroblast differentiation-associated protein AHNAK	S	210	0,01043152	-2,0795	2,4981E-09

Table S9. Unsupervised hierarchical clustering of differentially expressed phosphosites when comparing AML patient cells showing strong versus weak antiproliferative effects of V-ATPase inhibition. The table lists the differentially expressed phosphosites from the top and downwards for the heatmap presented in Figure 3A (gene name, protein, amino acid, amino acid residue, summary of the phosphosite identity).

**UPPER MAIN CLUSTER OF FIGURE 3A** Proteins showing significantly increased levels in AML cells with a weak antiproliferative effect of V-ATPase inhibition (43 phosphosites)				
**Gene name**	**Protein**	**AA**	**Residue**	**Summary**
FOXK1	FOXK1	S	428	FOXK1_S428
MAVS	MAVS	S	222	MAVS_S222
LMNB2	LMNB2	S	419	LMNB2_S419
HNRNPM	HNRNPM	S	413	HNRNPM_S413
HNRNPM	HNRNPM	S	442	HNRNPM_S442
EIF4EBP1	EIF4EBP1	S	65	EIF4EBP1_S65
RCSD1	RCSD1	S	83	RCSD1_S83
FAM21A	FAM21A	S	288	FAM21A_S288
FAM21A	FAM21A	S	284	FAM21A_S284
RCSD1	RCSD1	T	124	RCSD1_T124
RCSD1	RCSD1	S	123	RCSD1_S123
RCSD1	RCSD1	S	120	RCSD1_S120
RCSD1	RCSD1	S	116	RCSD1_S116
BIN2	BIN2	S	277	BIN2_S277
BIN2	BIN2	S	273	BIN2_S273
SIPA1	SIPA1	S	74	SIPA1_S74
UHRF1BP1	UHRF1BP1	S	1106	UHRF1BP1_S1106
UHRF1BP1	UHRF1BP1	S	1103	UHRF1BP1_S1103
ARFGEF2	ARFGEF2	S	227	ARFGEF2_S227
ARFGEF2	ARFGEF2	S	218	ARFGEF2_S218
ZNF318	ZNF318	S	1945	ZNF318_S1945
AHNAK	AHNAK	S	210	AHNAK_S210
ELAC2	ELAC2	S	199	ELAC2_S199
TEX2	TEX2	S	196	TEX2_S196
AHNAK	AHNAK	S	5780	AHNAK_S5780
BRAT1	BRAT1	S	742	BRAT1_S742
PLA2G4A	PLA2G4A	S	729	PLA2G4A_S729
FNBP1	FNBP1	S	299	FNBP1_S299
AHNAK	AHNAK	S	210	AHNAK_S210
AHNAK	AHNAK	S	5780	AHNAK_S5780
AHNAK	AHNAK	S	5077	AHNAK_S5077
AHNAK	AHNAK	S	5731	AHNAK_S5731
OGFR	OGFR	S	378	OGFR_S378
RCSD1	RCSD1	S	120	RCSD1_S120
RCSD1	RCSD1	S	216	RCSD1_S216
FAM21A	FAM21A	S	284	FAM21A_S284
AHNAK	AHNAK	S	135	AHNAK_S135
AHNAK	AHNAK	S	5110	AHNAK_S5110
BAD	BAD	S	99	BAD_S99
GABPA	GABPA	S	303	GABPA_S303
CHAMP1	CHAMP1	S	432	CHAMP1_S432
FAM65B	FAM65B	S	341	FAM65B_S341
VIM	VIM	S	39	VIM_S39
**UPPER MAIN CLUSTER** Proteins showing significantly increased levels in AML cells with a strong antiproliferative effect of V-ATPase inhibition (57 phosphosites)				
TBC1D10C	TBC1D10C	T	415	TBC1D10C_T415
ACIN1	ACIN1	S	216	ACIN1_S216
HIST1H1E	HIST1H1E	T	18	HIST1H1E_T18
HIST1H1E	HIST1H1E	S	2	HIST1H1E_S2
CDYL	CDYL	S	147	CDYL_S147
CBX3	CBX3	S	93	CBX3_S93
SLC38A1	SLC38A1	S	52	SLC38A1_S52
SMARCA2	SMARCA2	S	172	SMARCA2_S172
RYBP	RYBP	S	123	RYBP_S123
RYBP	RYBP	S	127	RYBP_S127
RYBP	RYBP	S	130	RYBP_S130
FBL	FBL	S	124	FBL_S124
NUMA1	NUMA1	S	1955	NUMA1_S1955
NUMA1	NUMA1	S	1977	NUMA1_S1977
STMN1	STMN1	S	63	STMN1_S63
PATL1	PATL1	S	177	PATL1_S177
ATRIP	ATRIP	S	391	ATRIP_S391
CCNY	CCNY	S	326	CCNY_S326
LSM14A	LSM14A	S	183	LSM14A_S183

LSM14A	LSM14A	S	182	LSM14A_S182
UNCX	UNCX	S	449	UNCX_S449
UNCX	UNCX	S	443	UNCX_S443
LAT2	LAT2	S	102	LAT2_S102
PDCD4	PDCD4	S	302	PDCD4_S302
DDX54	DDX54	S	782	DDX54_S782
SERBP1	SERBP1	S	330	SERBP1_S330
RLF	RLF	S	634	RLF_S634
BOD1L1	BOD1L1	S	482	BOD1L1_S482
SLC20A1	SLC20A1	S	269	SLC20A1_S269
SLC20A1	SLC20A1	S	265	SLC20A1_S265
JUNB	JUNB	T	255	JUNB_T255
JUNB	JUNB	S	259	JUNB_S259
NBAS	NBAS	S	475	NBAS_S475
NBAS	NBAS	S	473	NBAS_S473
CDK12	CDK12	T	893	CDK12_T893
MIA2	MIA2	S	1243	MIA2_S1243
SF3B2	SF3B2	S	309	SF3B2_S309
SF3B2	SF3B2	S	307	SF3B2_S307
FLNA	FLNA	S	2150	FLNA_S2150
SUPT6H	SUPT6H	T	1532	SUPT6H_T1532
SUPT6H	SUPT6H	S	1535	SUPT6H_S1535
TRA2A	TRA2A	S	236	TRA2A_S236
HNRNPK	HNRNPK	S	379	HNRNPK_S379
GATAD2B	GATAD2B	S	129	GATAD2B_S129
SUZ12	SUZ12	S	546	SUZ12_S546
NFRKB	NFRKB	S	298	NFRKB_S298
RBM6	RBM6	S	746	RBM6_S746
HIST1H1E	HIST1H1E	T	18	HIST1H1E_T18
RPL12	RPL12	S	38	RPL12_S38
LSP1	LSP1	T	175	LSP1_T175
LSP1	LSP1	S	177	LSP1_S177
GLYR1	GLYR1	S	130	GLYR1_S130
HIST1H1C	HIST1H1C	S	36	HIST1H1C_S36
HIST1H1E	HIST1H1E	S	36	HIST1H1E_S36
HNRNPUL1	HNRNPUL1	S	94	HNRNPUL1_S94
SP100	SP100	S	18	SP100_S18
SURF6	SURF6	S	138	SURF6_S138

Table S10. Gene expression analysis of the V-ATPase interactome; analyses of the results for 32 unselected/consecutive AML patients. The table presents clinical and biological data for the patients listed from the top to the bottom of the patient clustering (Figure 7 in the article). The dark grey line indicates the separation between the upper and the lower main patient clusters. Secondary AML is marked with green color;Flt3-ITD is marked with grey.

Id	Inhib	Sex	Age	Previous	FAB	Karyotype	FLT3	NPM1	CD34
19	0.37	M	42		M2	Normal	ITD	wt	+
22	0.02	F	77	MDS	M1/2	Normal	ITD	INS	nt
28	0.57	F	57		M4	inv16	wt	wt	+
25	0.12	F	78		M1	Normal	ITD	INS	-
46	0.33	M	60		M4	del9	ITD	wt	+
55	0.23	F	55		M1	Normal	ITD	INS	+
30	0.66	M	20		M2	Normal	ITD	wt	+
71	0.72	F	59		M4	Normal	ITD	INS	-
21	0.1	F	18		M4	Inv16 (p13.1q22)	wt	wt	+
27		M	58		M5	Normal	wt	wt	+
54	0.18	F	77		M1	nt	wt	INS	-
17	0.43	M	68		M1	Normal	wt	wt	+
59	0.07	M	76		M0	Normal	wt	wt	+
20	0.09	M	24		M2	Complex	nt	wt	+
47		M	78		M1	t(9;22) +8, +10,+12+19+20+21der22	nt	nt	+
50		M	41		M1	t(8;21) del9-20 -22 -3mar	wt	wt	+
4	0.89	M	46		M1	Normal	wt	INS	nt
34	0.09	F	87		M0	del5 (q13q33)	wt	wt	+
70	0.95	F	80	Myelofibrosis	M2	Nt	nt	nt	+
60	0.39	M	78	MDS	M1	Nt	ITD	nt	+
3	0.01	F	77	MDS	M1	Normal	wt	wt	+
48	0.19	F	71	MDS	nt	del(12)	nt	nt	+
74	0.03	M	76	MDS	nt	Normal	nt	nt	+
13	0.16	M	36		M4	inv16	wt	wt	+
64	0.16	F	71	MDS	nt	t(1;5), t(2;3)	nt	nt	+
33	0.3	M	78	KMML	M4	+8	nt	nt	-
26	0.08	F	82		M4	Normal	ITD	wt	+
57	0.81	M	64		M5	Normal	wt	INS	-
12		M	48		M5	Normal	ITD	INS	-
51	0.14	M	76	KMML	M5	del12, -7	nt	nt	+
35	0.21	M	72	MDS	M4	Normal	nt	nt	-
29		M	82		nt	+8	wt	wt	+

Abbreviations: CMML, chronic myelomonocytic leukemia; MDS, myelodysplastic syndrome; nt, not tested; PV, polycytemia vera; wt, wild type.

Table S11. The effect of V-ATPase inhibitors on the viability of primary human AML cells derived from all 80 patients included in our study. The leukemic cells were incubated with bafilomycin A1 10 nM or concanamycin A 10 nM for 48 hours before cell viability was investigated by using flow cytometric analysis based on Annexin-propidium iodide (PI) staining. The overall results for all 80 patients are summarized in the table. For all patients we identified the three main cell subsets of viable AnnexinV$^-$PI$^-$, early apoptotic AnnexinV$^+$PI$^-$ and late apoptotic/necrotic Annexin$^+$PI$^+$ cells. The table presents the median percent and the variation range together with the results from the statistical analysis (*median* percentage and *variation range* for control and drug-containing cultures that were compared with the corresponding drug-free controls cultures. The Wilcoxon's test for paired samples was used for the statistical analyses and the p-value are presented in the right column.

**Biological parameter examined in medium controls and drug-containing cultures**	**Median (%)**	**Range (%)**	***p*-value (Wilcoxon's test)**
**Cell viability (AnnexinV$^-$PI$^-$)**			
Drug-free control	54.0	2.6-97.9	
Bafilomycin A1 10 nM	49.4	2.0-97.7	<0.0005
Concanamycin A 10 nM	16.2	0.6-95.2	<0.0005
**Early apoptotic cells (AnnexinV$^+$PI$^-$)**			
Drug-free control	3.6	0.2-39.1	
Bafilomycin A1 10 nM	4.1	0.3-25.3	0.104
Concanamycin A 10 nM	6.6	0.3-26.0	<0.0005
**Late apoptotic and necrotic cells (AnnexinV$^+$PI$^+$)**			
Drug-free control	38.7	1.9-95.0	
Bafilomycin A1 10 nM	44.3	2.0-95.9	<0.0005
Concanamycin A 10 nM	73.2	4.1-98.4	<0.0005

Table S12. Constitutive release of soluble mediators by primary human AML cells; comparison of cultures with the two V-ATPase inhibitors bafilomycin A1 and concanavalin A (10 nM) with drug-free medium controls. The results are presented as the number of patients with detectable release in control or drug-containing cultures (median level together with variation range). The p-values refer to the statistical comparison between inhibitor-containing and control cultures (Wilcoxon's test for paired samples). Green color indicates a significant increase whereas blue color indicates a significant decrease of the mediator level when comparing the results for all 80 patients (n.d., not detectable).

Mediators	Bafilomycin				Concanamycin				Control		
	Number	Median (pg/mL)	Range (pg/mL)	*p*-value	Number	Median (pg/mL)	Range (pg/mL)	*p*-value	Number	Median (pg/mL)	Range (pg/mL)
**Chemokines**											
CCL2	72	479	n.d. – 40,616	0.239	71	1125	n.d. – 35,099	0.466	73	360	n.d. – 45,780
CCL3	66	756	n.d. – 553,772	<0.0005	71	6486	n.d. - > 174,065	<0.0005	64	651	n.d. – 119,984
CCL4	63	547	n.d. – 64,731	<0.0005	69	1905	n.d. – 87,940	<0.0005	60	370	n.d. – 16,782
CCL5	79	137	3.8 – 13,934	<0.0005	80	535	21.8 – 7356	<0.0005	75	78.2	n.d. – 6853
CXCL1	76	90.6	n.d. - > 31,279	0.014	79	111	n.d. – 59,865	0.004	76	63.1	n.d. - > 13,502
CXCL5	63	247	n.d. – 142,272	0.027	69	197	n.d. – 84,623	0.011	61	218	n.d. – 151,194
CXCL8	78	2757	n.d. - > 18,561	<0.0005	80	6826	0.4 - > 26,000	<0.0005	80	2757	2.14 - >17,483.
CXCL10	56	27.2	n.d. – 3756	<0.0005	61	26.4	n.d. – 3513	0.006	54	12.0	n.d. – 3415
**Interleukins**											
IL-1B	48	5.7	n.d. – 3174	<0.0005	59	45.9	n.d. – 13,976	<0.0005	42	1.6	n.d. – 2813
IL-1ra	72	6562	n.d. - > 625,189	<0.0005	70	6467	n.d. – 397,214	0.380	69	4115	n.d. - > 241,847
IL-6	72	12.6	n.d. – 27,659	<0.0005	73	26.8	n.d. – 30,801	<0.0005	71	10.3	n.d. – 24,155
**TNF-A**	57	15.6	n.d. - > 1893	<0.0005	66	78.0	n.d. - > 5225	<0.0005	50	5.5	n.d. – 2766
**Proteases**											
MMP-1	65	314	n.d. – 297,454	0.206	68	355	n.d. – 47,207	0.254	66	210	n.d. – 79,752
MMP-2	63	3077	n.d. – 37,498	<0.0005	63	2209	n.d. – 19,563	0.213	62	2435	n.d. – 25,297
MMP-9	72	6072	n.d. – 196,007	0.666	74	341	n.d. – 198,523	0.0005	73	4875	n.d. – 191,664
**Protease Inhibitors**											
Cystatin C	75	3604	n.d. – 59,761	0.028	77	2695	n.d. – 18,0	<0.000	77	3828	n.d. – 86,867
Serpin E1	79	689	7.4 - > 36,405	<0.0005	80	694	6.0 – 21,5	0.350	80	449	8.11 – 72,208
**Growth factors**											
G-CSF	31	21.0	n.d. – 7,771	0.001	35	21.0	n.d. – 43,315	<0.0005	28	21.0	n.d. – 4474
HGF	51	24.8	n.d. – 962	0.026	48	15.0	n.d. – 271	<0.0005	50	19.3	n.d. – 964

Table S13. Effects of concanamycin A on the constitutive cytokine release by primary human AML cells. Leukemic cells cells from 80 patients were cultured for 48 hours in cytokine-supplemented growth medium with and without concanamycin A 10 nM before mediator levels were determined in the culture supernatants. The relative mediator level was determined for each patient and cytokine; i.e. the mediator level for the drug-containing culture relative to the same cytokine level in the corresponding medium control cultures. An unsupervised hierarchical clustering analysis was performed based on these relative mediator levels (Figure 9), and the patients are listed according to the clustering analysis in the table. A subset/subcluster of 19 patients with a relatively weak effect of concanamycin A on the cytokine levels formed a separate subcluster (indicated by grey color in the table.

Id	Sex	Age	Previous	FAB	Karyotype	FLT3	NPM-1	CD34
1	M	42		M2	Normal	ITD	wt	+
56	M	20		M2	Normal	ITD	wt	+
13	F	77	Secondary AML (MDS)	M1	Normal	wt	wt	+
40	F	71	Secondary AML (MDS)	nt	t(1;5), t(2;3)	nt	nt	+
60	M	76	Secondary AML (CMML)	M5	del12, -7	nt	nt	+
78	F	77		M1	nt	wt	INS	-
66	M	72	Secondary AML (MDS)	M4	Normal	nt	nt	-
2	M	81	Secondary AML (PV)	M2	-7	wt	wt	-
19	F	86		M2	nt	nt	nt	-
41	M	41		M4	Normal	wt	wt	nt
3	F	80	Secondary AML (CMF)	M2	nt	nt	nt	+
21	F	55		M2	Normal	ITD	INS	-
53	F	59		M4	Normal	ITD	INS	-
24	F	77	Secondary AML (MDS)	M1/2	Normal	ITD	INS	nt
4	M	82	Secondary AML (CMF)	M2	t(9;22)	wt	wt	+
34	F	59		M5	Normal	ITD	INS	-
61	F	74		M4	t(8;21)	ITD	INS	+
67	M	77		M2	+8, +6, +15, +20, +21, +22	ITD	wt	+
70	M	68		M1	Normal	wt	wt	+
11	M	78	Secondary AML (MDS)	M1	nt	ITD	nt	+
43	M	82		nt	+ 8	wt	wt	+
30	F	78		M1	Normal	ITD	INS	-
58	F	29		M5	Normal	ITD+As p835	wt	+
18	F	71	Secondary AML (MDS)	nt	del(12)	nt	nt	+
25	M	71		nt	nt	nt	nt	nt
74	F	87		M0	del5 (q13q33)	wt	wt	+
5	M	60		M5	t(10;11), +8	wt	wt	nt
22	M	71	Secondary AML (chemotherapy)	M4/5	nt	nt	nt	nt
80	M	19		M5	Normal	wt	wt	-
6	M	33		M4	Normal	ITD	INS	+
8	F	49	AML relapse	M2	Complex	nt	nt	+
9	M	32		M2	Normal	wt	wt	+
14	M	46		M1	Normal	wt	INS	nt
23	F	18		M4	Inv16 ((p13.1q22)	wt	wt	+
63	F	68		M5	Normal	wt	INS	-
28	F	75	Secondary AML (CMF)	M2	Nt	nt	nt	+
17	F	70		M4	Normal	wt	INS	-
47	M	65		M4	Normal	wt	INS	-
38	F	82		M4	Normal	ITD	wt	+
79	F	50		M2	-7, t(3;3) (q21.3;q26.2)	wt	wt	+
48	M	64		M5	Normal	wt	INS	-
54	M	61		M4	Nor6mal	wt	wt	-
64	M	65		M5	Complex	wt	INS	nt
45	F	55		M5	Normal	ITD	INS	-
15	M	46		M1	Normal	wt	INS	nt
31	F	67		M0	+21	wt	wt	+
76	F	71		M0	Normal	wt	INS	-
77	F	46		M2	i(13) subclone	wt	wt	+
42	M	53		M0	+13	wt	wt	+
59	F	63		M1	Normal	wt	wt	+
29	F	45		M4	Normal	wt	INS	-
57	F	64		M2	Normal	ITD	INS	-
37	M	76	Secondary AML (MDS)	nt	Normal	nt	nt	+
72	M	76		M0	Normal	wt	wt	+
16	M	68	Secondary AML (CMF)	M4	Normal	Asp835	wt	+

62	M	78		M0	Complex	wt	wt	-
35	M	41		M1	t(8;21) del9-20 -22 -3mar	wt	wt	+
10	F	51	AML relapse	M0	Complex	wt	wt	+
52	F	92	Secondary AML (chemotherapy)	M1	nt	nt	nt	-
32	M	60	Secondary AML (MDS)	M2	Normal	nt	nt	+
50	M	78		M1	t(9;22) +8, +10,+12+19+20+21der22	nt	nt	+
46	M	62		M1	Normal	wt	wt	+
22	M	24		M2	Complex	nt	wt	+
20	M	65		M5	t(16;16)	wt	wt	+
51	M	36		M4	inv16	wt	wt	+
55	F	57		M4	inv16	wt	wt	+
65	F	64	AML relapse	M4	42-46, XX, -16,-22 [cp25]	wt	wt	+
26	F	46		M1	inv16	wt	wt	+
73	M	62		M4	+ 8	wt	wt	+
44	M	58		M5	Normal	wt	wt	+
36	M	35		M2	Normal	wt	wt	+
33	M	60		M4	del9	ITD	wt	+
27	M	65	Secondary AML (MDS)	M1	Normal	wt	INS	-
75	M	59	Secondary AML (CMML)	M5	del20, +8	ITD	wt	+
49	M	48		M5	Normal	ITD	INS	-
68	F	60		M5	Normal	ITD	INS	-
7	M	78	Secondary AML (CMML)	M4	+8	nt	nt	-
39	M	72	Secondary AML (MDS)	M1	Complex	wt	nt	+
69	F	55		M1	Normal	ITD	INS	+
71	M	54		M5	Normal	wt	INS	-

Abbreviations: CMF, chronic myelofibrosis; CMML, chronic myelomonocytic leukemia; MDS, myelodysplastic syndrome; nt, not tested; PV, polycytemia vera; wt, wild type.

Table S14. Effects of bafilomycin A1 on the constitutive cytokine release by primary human AML cells. Leukemic cells from 80 patients were cultured for 48 hours in cytokine-supplemented growth medium with and without bafilomycin A1 10 nM before mediator levels were determined in the culture supernatants. The relative mediator level was determined for each patient and cytokine; i.e. the mediator level for the drug-containing culture relative to the same cytokine level in the corresponding medium control cultures. An unsupervised hierarchical clustering analysis was performed based on these relative mediator levels (Figure S5), and the patients are listed according to the clustering analysis in the table. A subset/subcluster of 19 patients with a relatively weak effect of bafilomycin A1 on the cytokine levels formed a separate subcluster (indicated by grey color in the table.

Id	Sex	Age	Previous	FAB	Karyotype	FLT3	NPM-1	CD34
1	M	42		M2	Normal	ITD	wt	+
63	F	68		M5	Normal	wt	INS	-
34	F	59		M5	Normal	ITD	INS	-
76	F	71		M0	Normal	wt	INS	-
36	M	35		M2	Normal	wt	wt	+
44	M	58		M5	Normal	wt	wt	+
40	F	71	Secondary AML (MDS)	nt	t(1;5), t(2;3)	nt	nt	+
61	F	74		M4	t(8;21)	ITD	INS	+
2	M	81	Secondary AML (PV)	M2	-7	wt	wt	-
3	F	80	Secondary AML (CMF)	M2	nt	nt	nt	+
67	M	77		M2	+8, +6, +15, +20, +21, +22	ITD	wt	+
17	F	70		M4	Normal	wt	INS	-
5	M	60		M5	t(10;11), +8	wt	wt	nt
6	M	33		M4	Normal	ITD	INS	+
53	F	59		M4	Normal	ITD	INS	-
31	F	67		M0	+21	wt	wt	+
11	M	78	Secondary AML (MDS)	M1	nt	ITD	nt	+
78	F	77		M1	nt	wt	INS	-
22	M	71	Secondary AML (chemotherapy)	M4/5	nt	nt	nt	nt
54	M	61		M4	Normal	wt	wt	-
24	F	77	Secondary AML (MDS)	M1/2	Normal	ITD	INS	nt
48	M	64		M5	Normal	wt	INS	-
72	M	76		M0	Normal	wt	wt	+
21	F	55		M2	Normal	ITD	INS	-
80	M	19		M5	Normal	wt	wt	-
60	M	76	Secondary AML (CMML)	M5	del12, -7	nt	nt	+
47	M	65		M4	Normal	wt	INS	-
74	F	87		M0	del5 (q13q33)	wt	wt	+
18	F	71	Secondary AML (MDS)	nt	del(12)	nt	nt	+
9	M	32		M2	Normal	wt	wt	+
77	F	46		M2	i(13) subclone	wt	wt	+
49	M	48		M5	Normal	ITD	INS	-
20	M	65		M5	t(16;16)	wt	wt	+
26	F	46		M1	inv16	wt	wt	+
23	F	18		M4	inv16 (p13.1q22)	wt	wt	+
55	F	57		M4	inv16	wt	wt	+
27	M	65	Secondary AML (MDS)	M1	Normal	wt	INS	-
28	F	75	Secondary AML (CMF)	M2	nt	nt	nt	+
51	M	36		M4	inv16	wt	wt	+
10	F	51	AML relapse	M0	Complex	wt	wt	+
50	M	78		M1	t(9;22) +8, +10,+12+19+20+21der22	nt	nt	+
16	M	68	Secondary AML (CMF)	M4	Normal	Asp835	wt	+
62	M	78		M0	Complex	wt	wt	-
32	M	60	Secondary AML (MDS)	M2	Normal	nt	nt	-
73	M	62		M4	+8	wt	wt	+
15	M	46		M1	Normal	wt	INS	nt
59	F	63		M1	Normal	wt	wt	+
43	M	82		nt	+8	wt	wt	+
66	M	72	Secondary AML (MDS)	M4	Normal	nt	nt	-
22	M	24		M2	Complex	nt	wt	+
57	F	64		M2	Normal	ITD	INS	-
42	M	53		M0	+ 13	wt	wt	+
4	M	82	Secondary AML (CMF)	M2	t(9;22)	wt	wt	+
41	M	41		M4	Normal	wt	wt	nt
68	F	60		M5	Normal	ITD	INS	-

71	M	54		M5	Normal	wt	INS	-
30	F	78		M1	Normal	ITD	INS	-
56	M	20		M2	Normal	ITD	wt	+
58	F	29		M5	Normal	ITD+ Asp835	wt	+
45	F	55		M5	Normal	ITD	INS	-
64	M	65		M5	Complex	wt	INS	nt
25	M	71		nt	nt	nt	nt	nt
38	F	82		M4	Normal	ITD	wt	+
79	F	50		M2	-7, t(3;3) (q21.3;q26.2)	wt	wt	+
65	F	64	AML relapse	M4	42-46, XX, -16,-22 [cp25]	wt	wt	+
46	M	62		M1	Normal	wt	wt	+
33	M	60		M4	del9	ITD	wt	+
13	F	77	Secondary AML (MDS)	M1	Normal	wt	wt	+
52	F	92	Secondary AML (chemotherapy)	M1	nt	nt	nt	-
75	M	59	Secondary AML (CMML)	M5	del20, +8	ITD	wt	+
7	M	78	Secondary AML (CMML)	M4	+8	nt	nt	-
70	M	68		M1	Normal	wt	wt	+
8	F	49	AML relapse	M2	Complex	nt	nt	+
35	M	41		M1	t(8;21) del9-20 -22 -3mar	wt	wt	+
37	M	76	Secondary AML (MDS)	nt	Normal	nt	nt	+
69	F	55		M1	Normal	ITD	INS	+
39	M	72	Secondary AML (MDS)	M1	Complex	wt	nt	+
19	F	86		M2	nt	nt	nt	+
29	F	45		M4	Normal	wt	INS	-
14	M	46		M1	Normal	wt	INS	nt

Abbreviations: CMF, chronic myelofibrosis; CMML, chronic myelomonocytic leukemia; MDS, myelodysplastic syndrome; nt, not tested; PV, polycytemia vera; wt, wild type..

Table S15. Clinical and biological characteristics of the 18 patients included in the studies soluble mediator release during co-culture of primary AML cells and normal MACs. The patients are listed according to the patient clustering presented in Figure 11 in the article. The bafilomycin A1/concanamycin A results are presented as the relative responses, i.e. the proliferation/viability in drug-containing cultures relative to the proliferation/viability in drug-free medium controls.

Id		Sex	Age	Previous	FAB	Karyotype	FLT3	NPM-1	CD34	Proliferation Bafilomycin A1 10 nM	Viability Concanamycin A 1 nM
60	1	M	76	Secondary AML (CMML)	M5	del12, -7	nt	nt	+	0.14	0.20
63	2	F	68		M5	Normal	wt	INS	-	0.60	0.11
3	3	F	80	Secondary AML (CMF)	M2	nt	nt	nt	+	0.95	0.49
18	4	F	71	Secondary AML (MDS)	nt	del(12)	nt	nt	+	0.19	0.47
48	5	M	64		M5	Normal	wt	INS	-	0.81	0.23
74	6	F	87		M0	del5 (q13q33)	wt	wt	+	0.09	0.51
24	7	F	77	Secondary AML (MDS)	M1/2	Normal	ITD	INS	nt	0.07	0.43
56	8	M	20		M2	Normal	ITD	wt	+	0.81	0.54
11	9	M	78	Secondary AML (MDS)	M1	nt	ITD	nt	+	0.39	0.22
43	10	M	82		nt	+8	wt	wt	+	nt	0.34
1	11	M	42		M2	Normal	ITD	wt	+	0.37	0.27
50	12	M	78		M1	t(9;22) +8, +10,+12+19+20 +21der22	nt	nt	+	nt	1.03
51	13	M	36		M4	inv16	wt	wt	+	0.16	0.44
26	14	F	46		M1	inv16	wt	wt	+	0.38	0.41
53	15	F	59		M4	Normal	ITD	INS	-	0.72	0.78
69	16	F	55		M1	Normal	ITD	INS	+	0.23	0.65
23	17	F	18		M4	inv16 (p131q22)	wt	wt	+	0.10	0.25
55	18	F	57		M4	inv716	wt	wt	+	0.57	0.48

Abbreviations: CMF, chronic myelofibrosis; CMML, chronic myelomonocytic leukemia; MDS, myelodysplastic syndrome; nt, not tested (proliferation corresponding to <1000 cpm); PV, polycytemia vera; wt, wild type.

Table S16. Constitutive release of soluble mediators by MSC co-culture of primary human AML cells derived from 18 unselected patients. Cells were co-cultured with and without drugs for 48 h before supernatants were harvested and cytokine levels determined. The results are presented as the median and range for the inhibitor-containing and corresponding control cultures. For each of the inhibitors we also present the p-value for the corresponding inhibitor-containing cultures compared with the corresponding controls (Wilcoxon's test for paired samples).

**Mediators**	**Bafilomycin (BAF) 10nM (AML+MSC)**			**Concanamycin (CCA) 10nM (AML+MSC)**			**Control (CTRL) (AML+MSC)**	
	**Median (pg/mL)**	**Range (pg/mL)**	***p*-value**	**Median (pg/mL)**	**Range (pg/mL)**	***p*-value**	**Median (pg/mL)**	**Range (pg/mL)**
**Chemokines**								
CCL2	870	171-29,300	0.500	268	148-6033	↓0.001	1018	164-30,730
CCL3	807	627-4653	0.163	757	586-2535	0.349	779	640-1771
CCL4	1517	1270-5953	0.981	1429	1176-2278	↓0.002	1495	1278-3694
CXCL1	450	210-5169	↓0.030	246	176-2268	↓<0.0005	684	210-7548
CXCL5	596	294-12,346	↓0.004	434	265-1204	↓<0.0005	696	323-23,492
CXCL8	6652	618-29,830	0.056	5572	536-27892	↓0.008	6652	633-28,166
CXCL10	11.9	7.4-3410	↑0.017	9.0	6.8-154	↓0.022	10.7	7.1-3116
**Interleukins**								
IL-1B	20.8	12.1-147	0.427	18.0	11.0-96.4	0.199	20.9	14.3-79.2
IL-1ra	943	297-18,494	0.446	722.	257-3209	↓0.001	936	295-14,493
IL-6	881	228-9825	0.071	951	224-9057	↑0.011	746	213-6246
**TNF-A**	35.3	23.8-141	0.758	32.6	20.0-136	0.122	35.3	25.1-102
**Proteases**								
MMP-1	326	218-1493	↑0.008	284	201-2049	↓0.044	311	210-864
MMP-2	28,808	20,119-32,704	0.500	25,065	16,428-29,343	↓<0.0005	28,649	19,279-33,336
**Protease Inhibitors**								
Cystatin C	5097	↓3234-10,733	↓<0.0005	3752	2325-6604	↓<0.0005	5635	3488-13,522
Serpin E1	37,251	n.d.-84,584	↓0.034	30,036	n.d.-45,576	↓0.002	41,554	n.d.-82,993
**Growth factors**								
GM-CSF	22.4	11.5-202	0.616	15.0	10.4-77.9	↓0.007	23.2	8.2-191
HGF	70.6	26.2-305	↑0.022	49.2	19.7-125	↓0.028	61.5	21.9-223

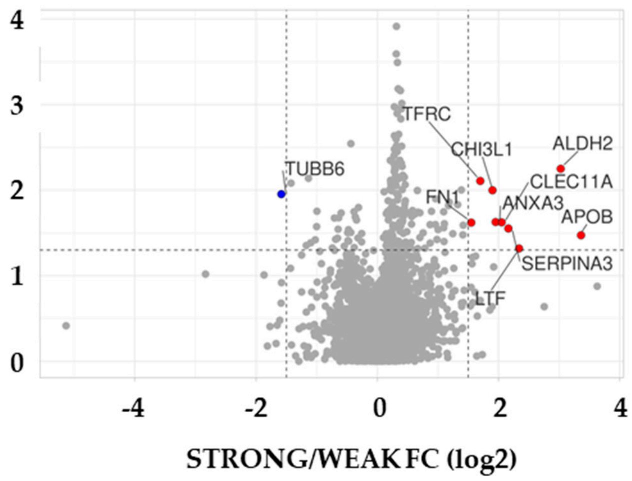

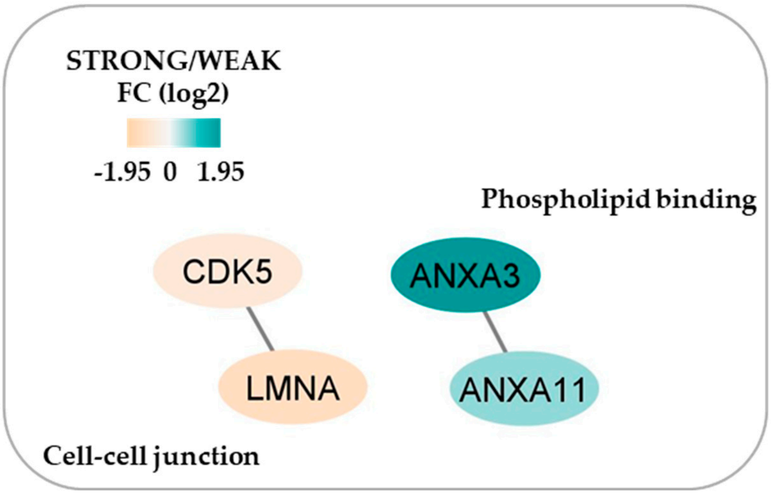

Figure S1. A comparison of the global primary AML cell proteome for leukemic cells characterized by either a strong antiproliferative effect of bafilomycin A1 10 nM (eight patients, relative response ≤0.30) or a weak effect (seven patients, ≥0.60). (UPPER) The Volcano plot analysis is based on differentially expressed proteins quantified for at least five patients per group, and the indicated points above the non-axial horizontal grey line represent proteins with significantly different abundances ($P < 0.05$). (LOWER) Protein–protein interactions (PPI) networks of differentially expressed proteins based on the STRING database, visualized and analyzed with Cytoscape. The protein nodes are colored according to their strong/weak antiproliferative effects log2 fold change (FC), i.e., green indicates increased abundance in the strong effect group and light orange indicates increased abundance in the weak antiproliferative effect group.

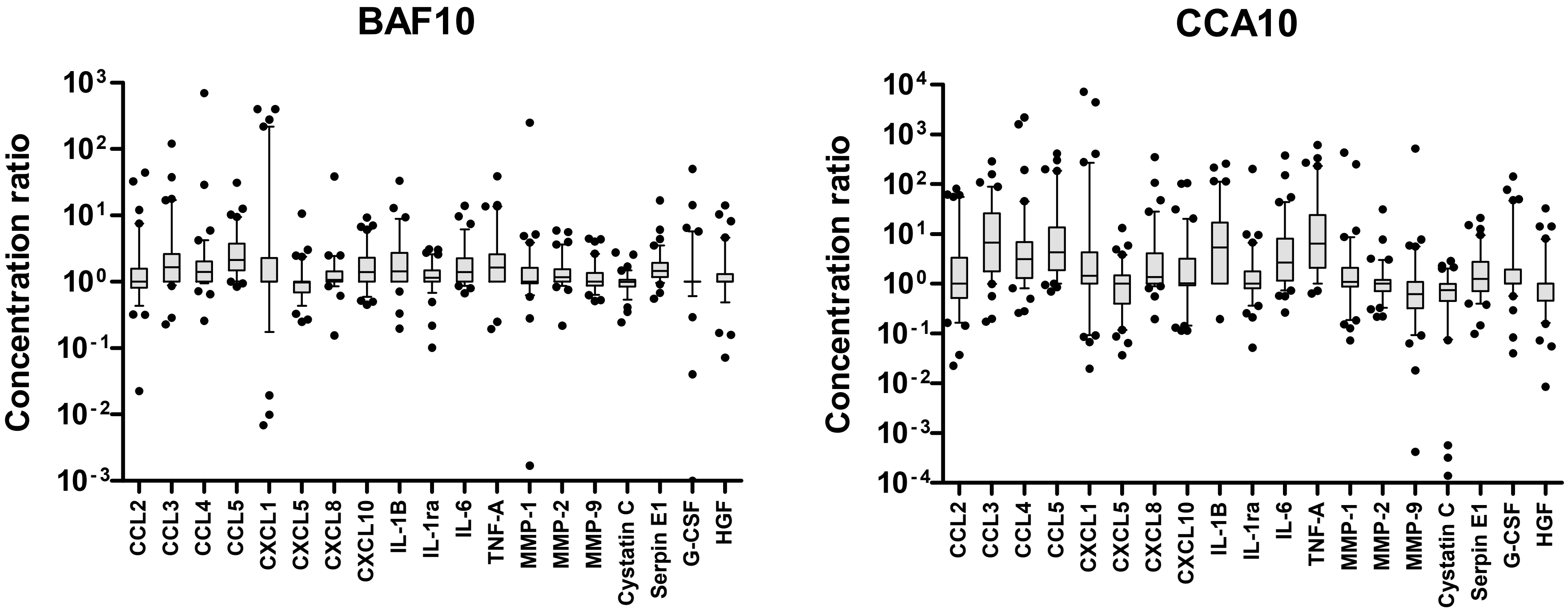

Figure S2. The constitutive release of soluble mediators by primary AML cells derived from 80 patients. The results for each of the mediators are presented as the median, 25-75 percentiles (boxes) and the 09 % confidence interval (whiskers). Outliers are marked as separate dots. All results are presented as the relative release, i.e. the release in drug-containing cultures relative to the corresponding drug-free controls. Both V-ATPase inhibitors were tested at 10 nM.

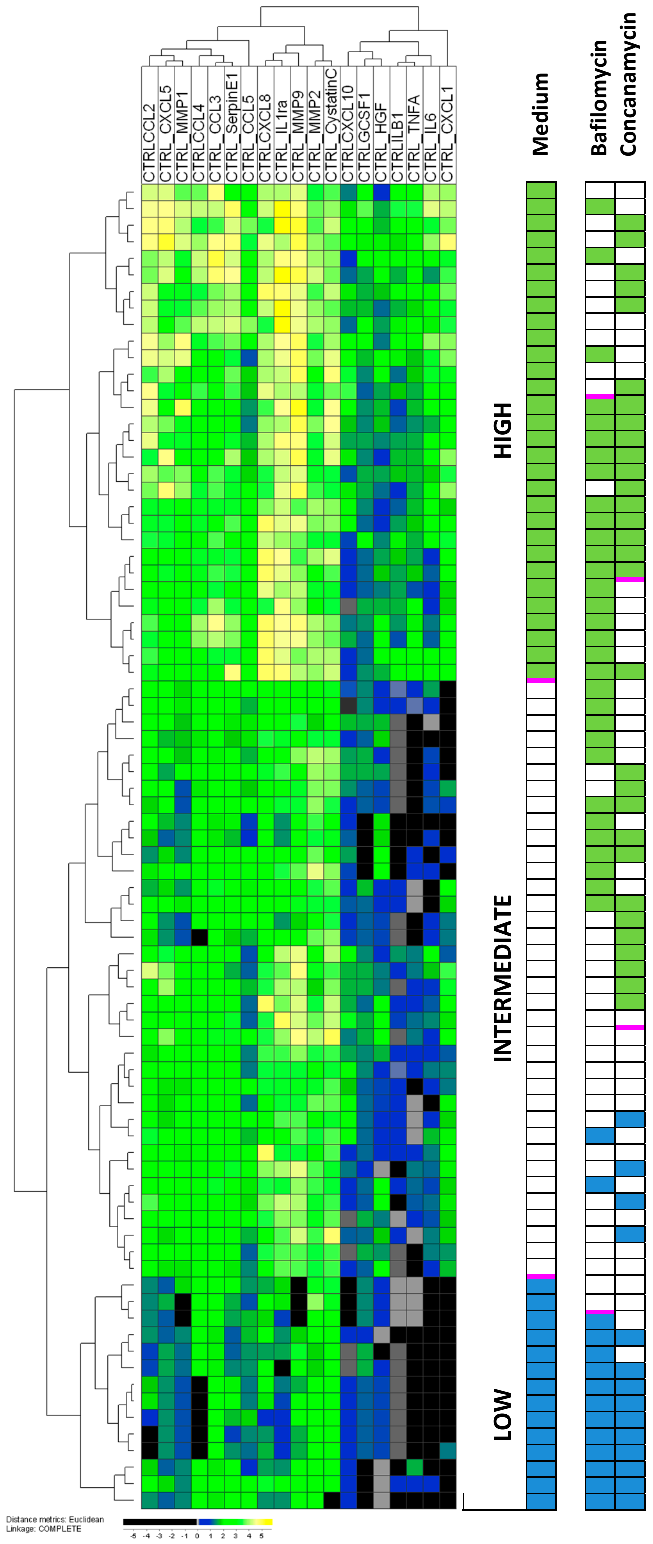

Figure S3. The patient profiles of absolute soluble mediator levels when primary AML cells were cultured either in medium alone, in the presence of bafilomycin 10 nM or concanamycin A 10 nM.. (LEFT) Primary human AML cells derived from 78 patients were cultured for 48 hours in cytokine-supplemented growth medium alone; the left part of the figure shows the results from an unsupervised hierarchical clustering analysis based on these control clusters (distance metrics Euclidian; Linkage Complete, black color indicates undetectable levels, yellow color indicates a level five times higher than the median level. (RIGHT) We compared similar unsupervised hierarchical clustering analyses in the right part of the figure; the three columns show the patient clustering for the control cultures, cultures with bafilomycin A1 10 nM (see Figure S4 left) and concanamycin A 10 nM (see Figure S4 right). The **pink lines** indicate separation between clusters/subclusters. All three clustering analyses identified two main clusters. A lower minor cluster included patients with generally low mediator levels: the **blue color** (LOW) indicates the patients in this cluster for the control culture clustering. The upper main cluster included patients with generally higher levels; this main cluster could be further divided into two subclusters indicated by **green** (HIGH, upper subcluster) and white (INTERMEDIATE, lower subcluster) respectively for the control culture clustering analysis. The positions for the green/white/blue patients in the control culture clustering is indicated in the middle and right columns for bafilomycin A1 and concanamycin A, respectively. Thus, most patients clustering together/close to each other based on control clusters cluster close to each other also after culture with bafilomycin A1 or concanamycin A.

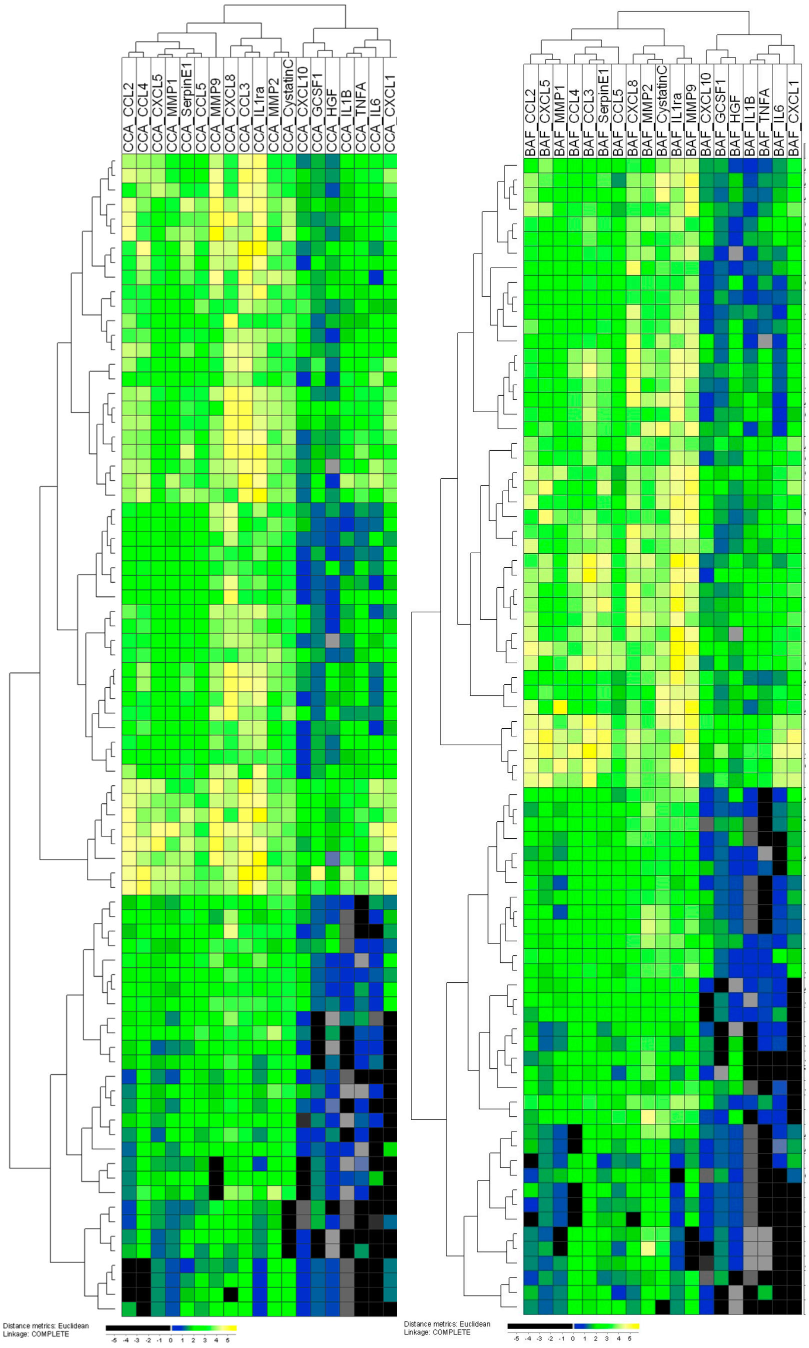

Figure S4. The patient profiles of absolute soluble mediator levels when primary AML cells were cultured in the presence of bafilomycin 10 nM (LEFT) or concanamycin A 10 nM. (RIGHT) Primary human AML cells derived from 80 patients were cultured for 48 hours in cytokine-supplemented growth medium supplemented with either bafilomycin A1 or concanamycin A. The figure presents the unsupervised hierarchical clustering analyses for each of the two drugs. Each of the two analyses identified two main patient cluster. The lower main cluster included patients with generally low levels. The upper main cluster included the majority of the patients and could be further subdivided into two subclusters; the upper subcluster included patients with generally high soluble mediator levels whereas the lower subcluster included patients with intermediate levels. The black color indicates undetectable level.

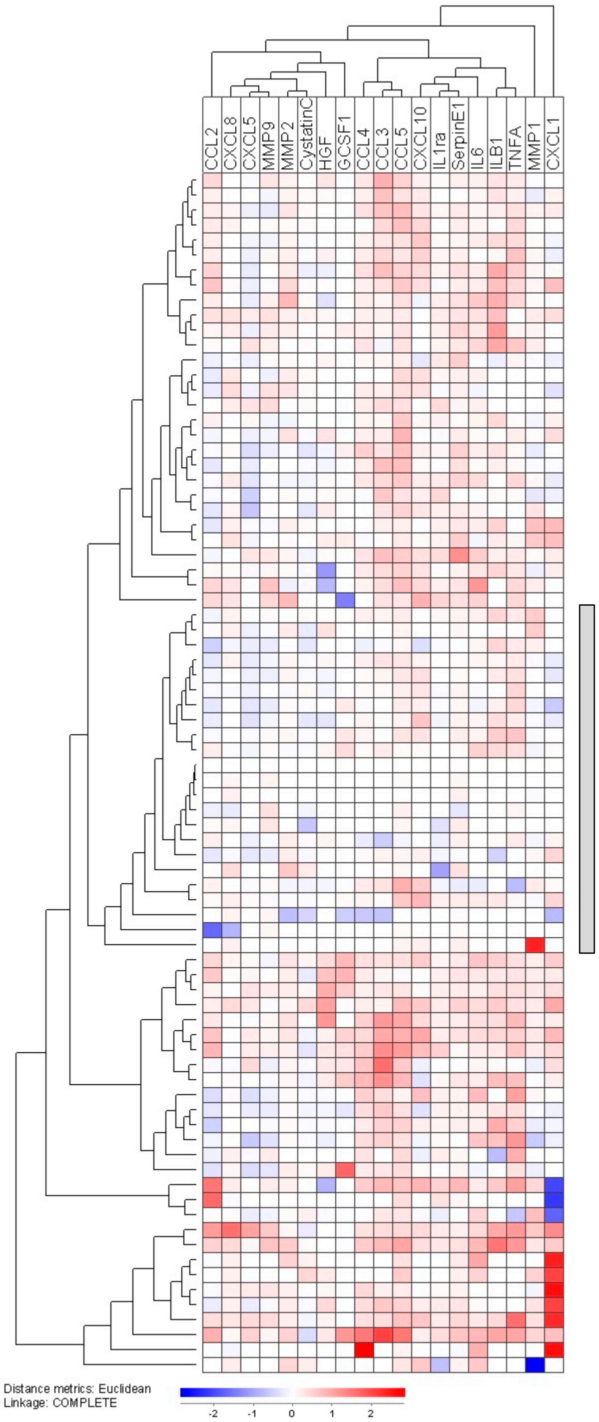

Figure S5. Effects of bafilomycin A1 on the constitutive cytokine release by primary human AML cells. Primary human AML cells from 80 patients were cultured for 48 hours in cytokine-supplemented growth medium with and without concanamycin 10 nM before mediator levels were determined in the culture supernatants. The relative mediator level was determined for each patient and cytokine; i.e. the mediator level for the drug-containing culture relative to the same cytokine level in the corresponding medium control cultures. An unsupervised hierarchical clustering analysis was performed based on these relative mediator levels. A subset of 23 patients with a relatively weak effect of concanamycin (white color indicates no difference between control cultures and bafilomycin A1 cultures) formed a separate subcluster (indicated by the grey column to the right), and 11 of these patients were also included among 19 patients that formed a separate low-effect cluster when testing the effects of concanamycin A.

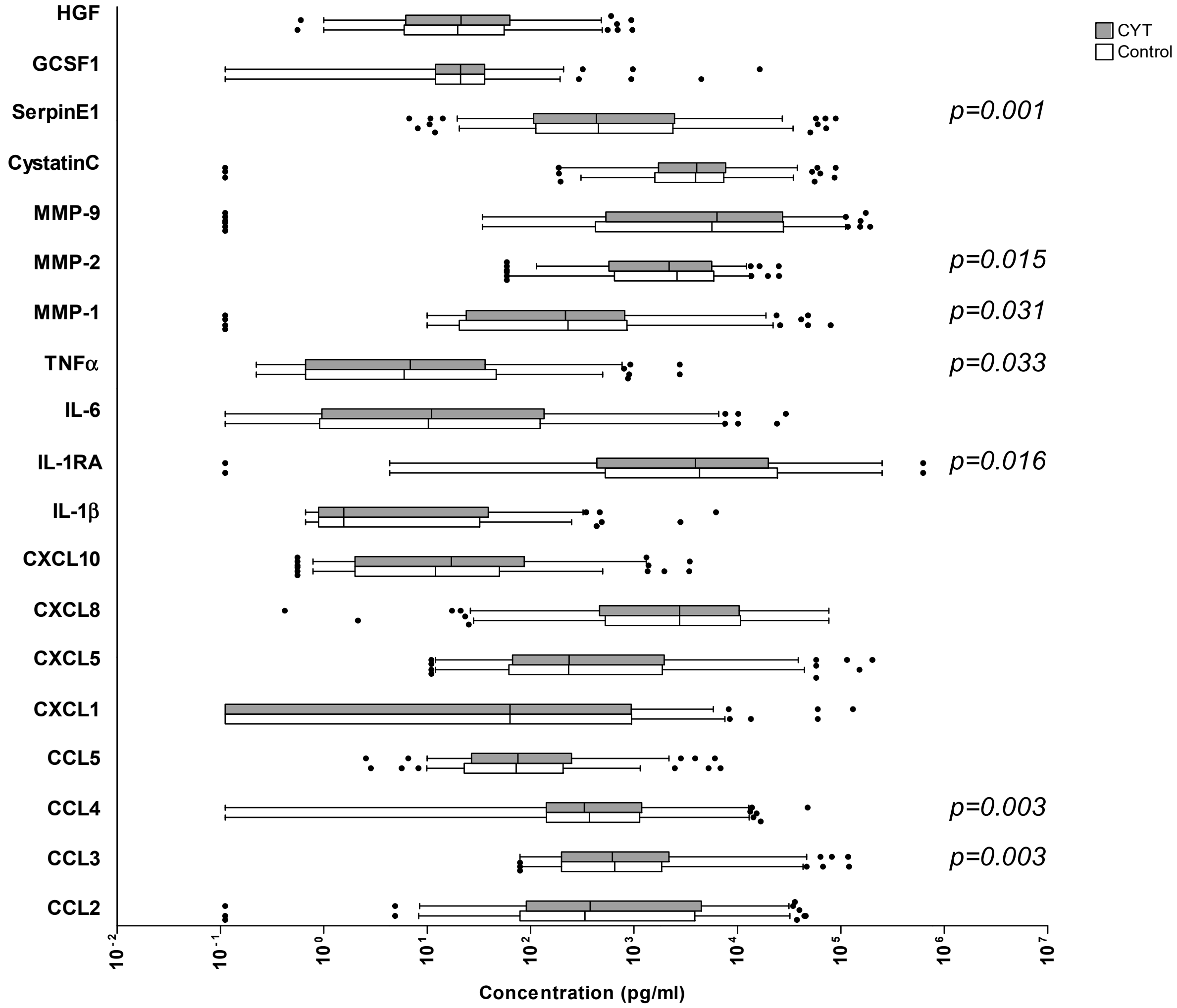

Figure S6. Cytarabine has only minor effects on the soluble mediator release profile of primary human AML cells. Primary human AML cells derived from the 78 patients were cultured in vitro either in medium alone (white columns) or medium supplemented with cytarabine 10 nM (dark columns). Cells were cultured for 48 hours before supernatants were harvested and the mediator concentrations determined. As can be seen from Table 2 the constitutive cytokine release profile differed between patients when the leukemic cells were cultured in medium alone. The results are presented as the median level, the 25/75 percent percentiles and the 95% confidence interval. Outliers are indicated in the figure. The figure presents the results for 19 soluble mediators. Cytarabine had no significant effect for 12 mediators and a strong effect corresponding to an uncorrected p-value <0.01 was only seen for CCL3, CCL4 and Serpin E1.

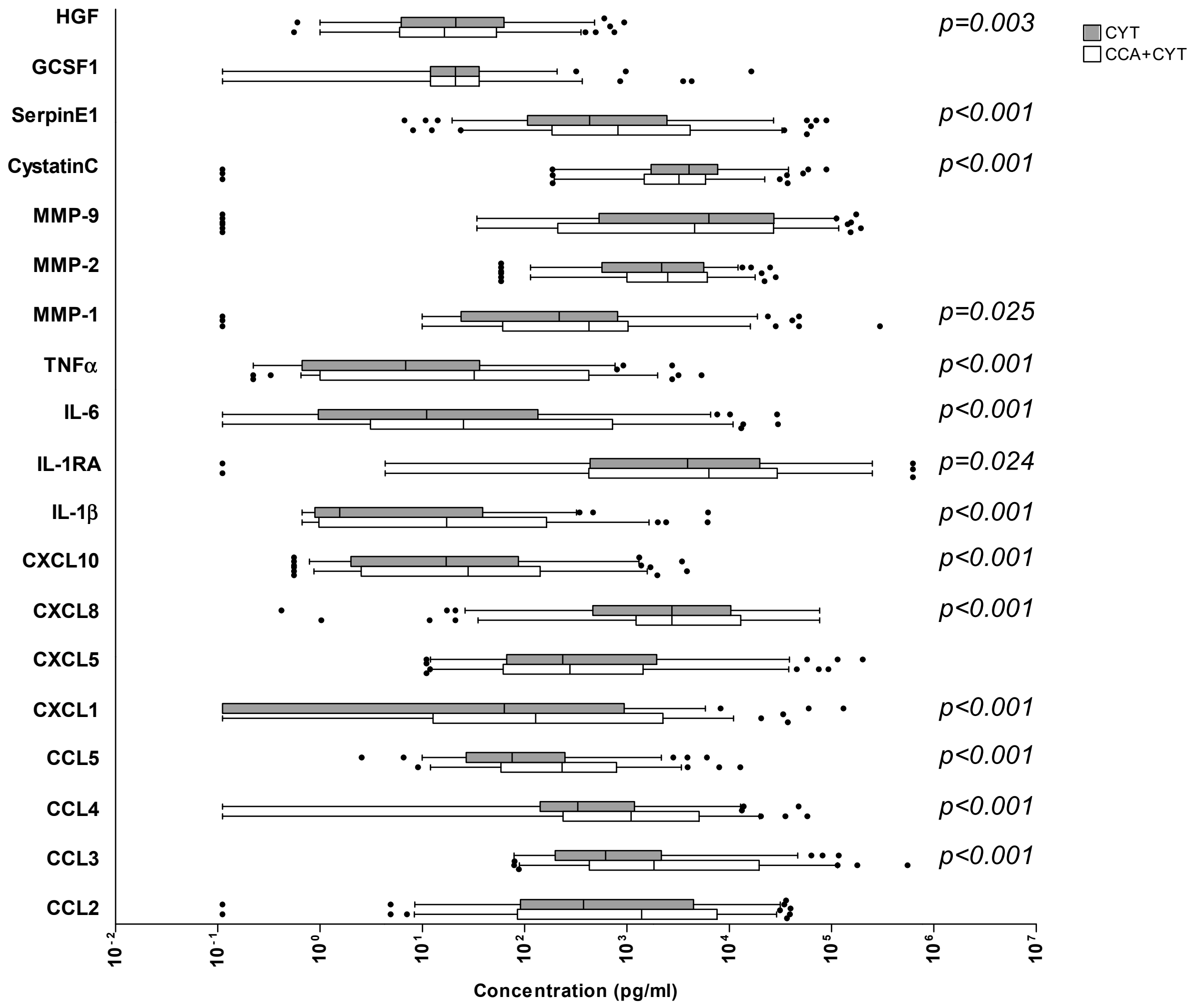

Figure S7. V-ATPase inhibition causes an increased constitutive soluble mediator release by primary human AML cells also in the presence of cytarabine. Primary human AML cells derived from the 80 patients were cultured in vitro either in medium supplemented with cytarabine 10 nM alone (dark columns) or cytarabine plus concanamycin A 1nM (white columns). Cells were cultured for 48 hours before supernatants were harvested and the mediator concentrations determined. The results are presented as the median level, the 25/75 percent percentiles and the 95% confidence interval. Outliers are indicated in the figure. The figure presents the results for 19 soluble mediators. Concanamycin A increased the levels for 14 mediators and a strong increase corresponding to a p-value <0.001 was seen for 14 mediators.

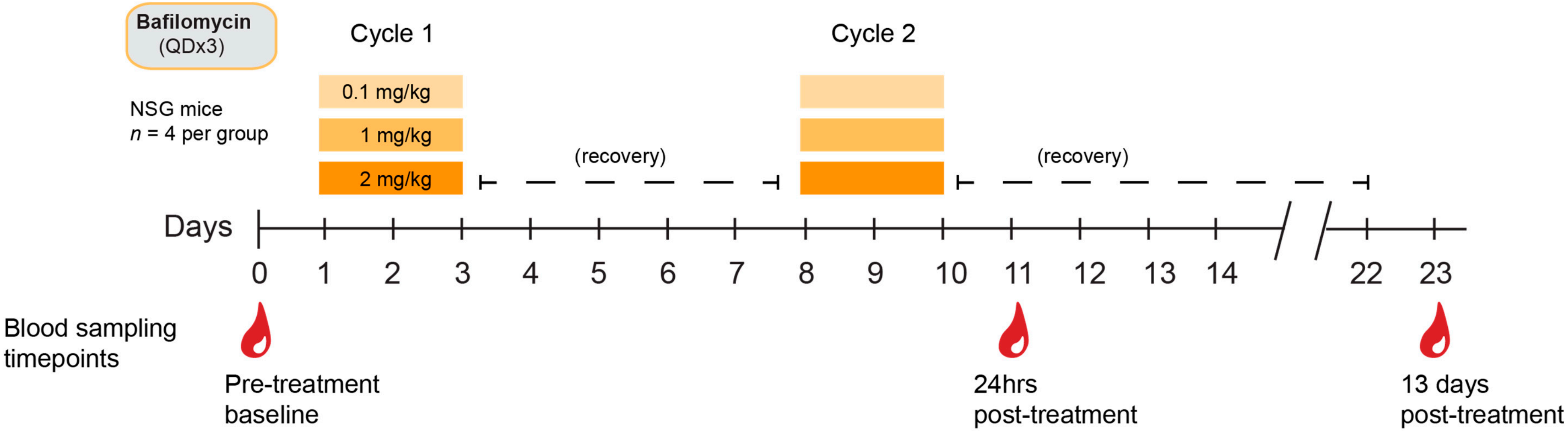

Figure S8. Evaluation of in vivo bafilomycin toxicity; studies of normal peripheral blood cell counts

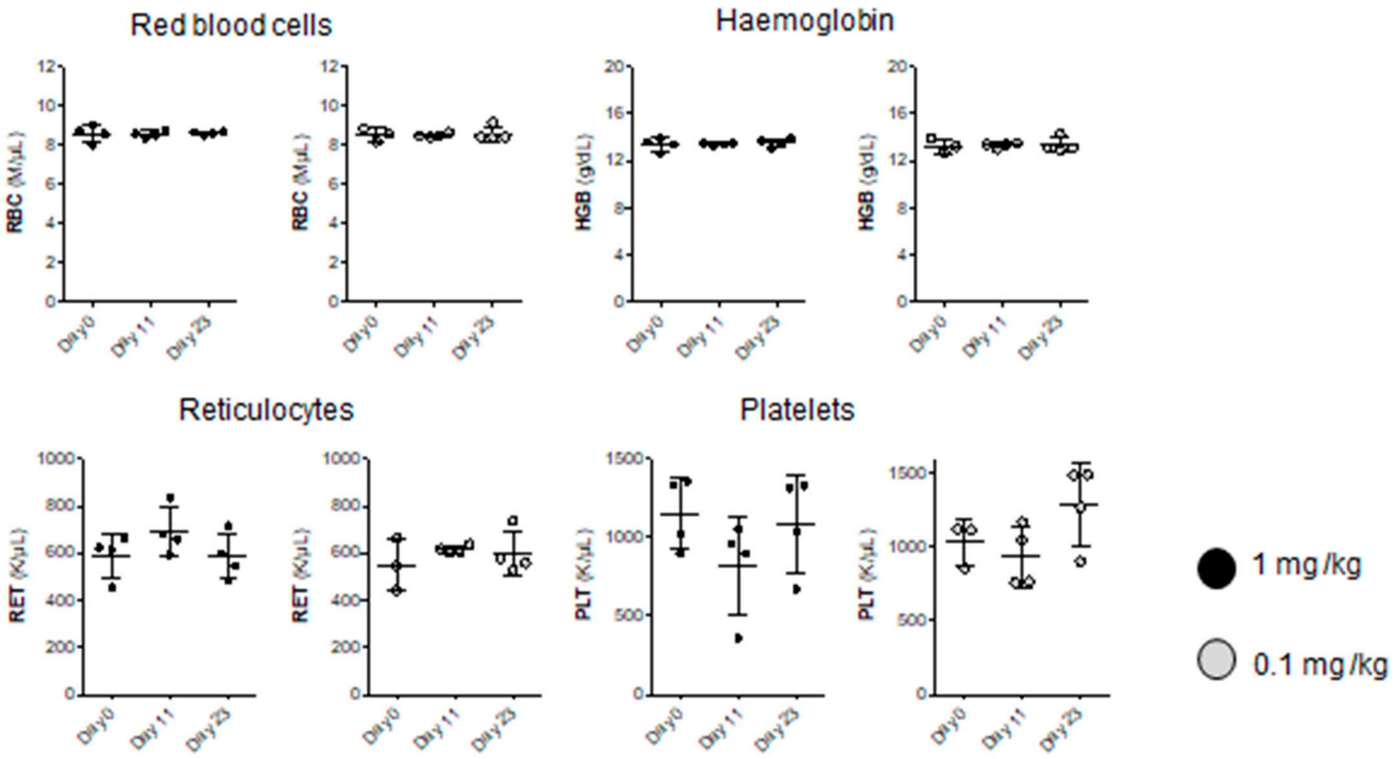

Figure S9. Effects on bafilomycin on erythrocytes and platelets. The animals (four mice for each concentration) were treated with bafilomycin for days 1-3 and 8-10, and peripheral blood cell counts were determined on days 0 (pretreatment), day 11 immediately after the second treatment and day 23 during regeneration.

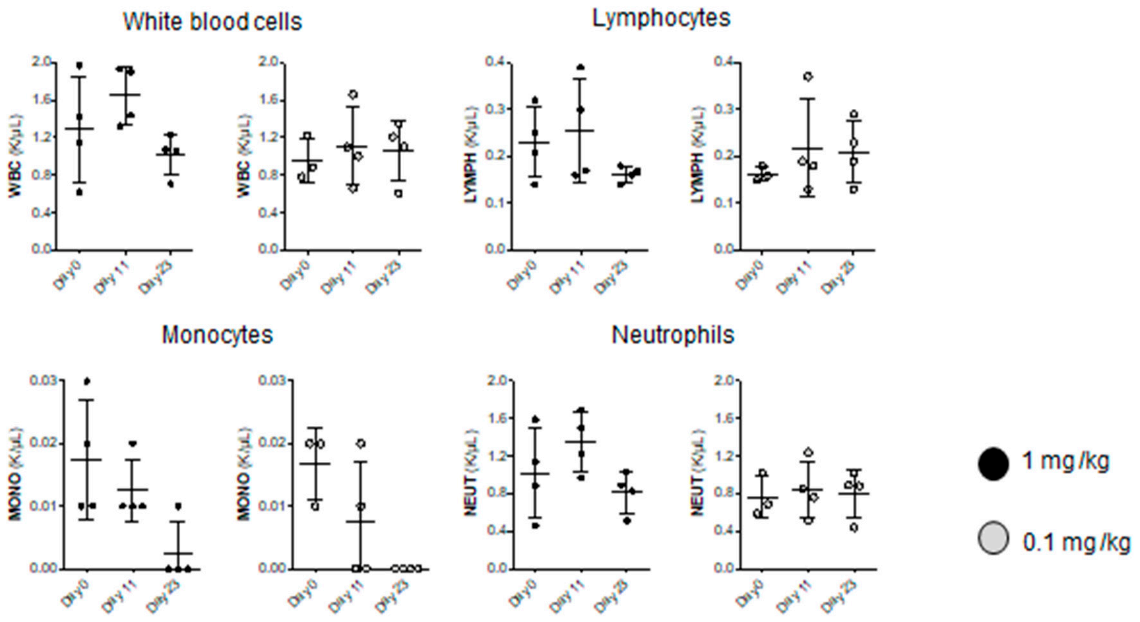

Figure S10. Effects on bafilomycin on normal leukocytes. The animals (four mice for each concentration) were treated with bafilomycin for days 1-3 and 8-10, and peripheral blood cell counts were determined on days 0 (pretreatment), day 11 immediately after the second treatment and day 23 during regeneration.

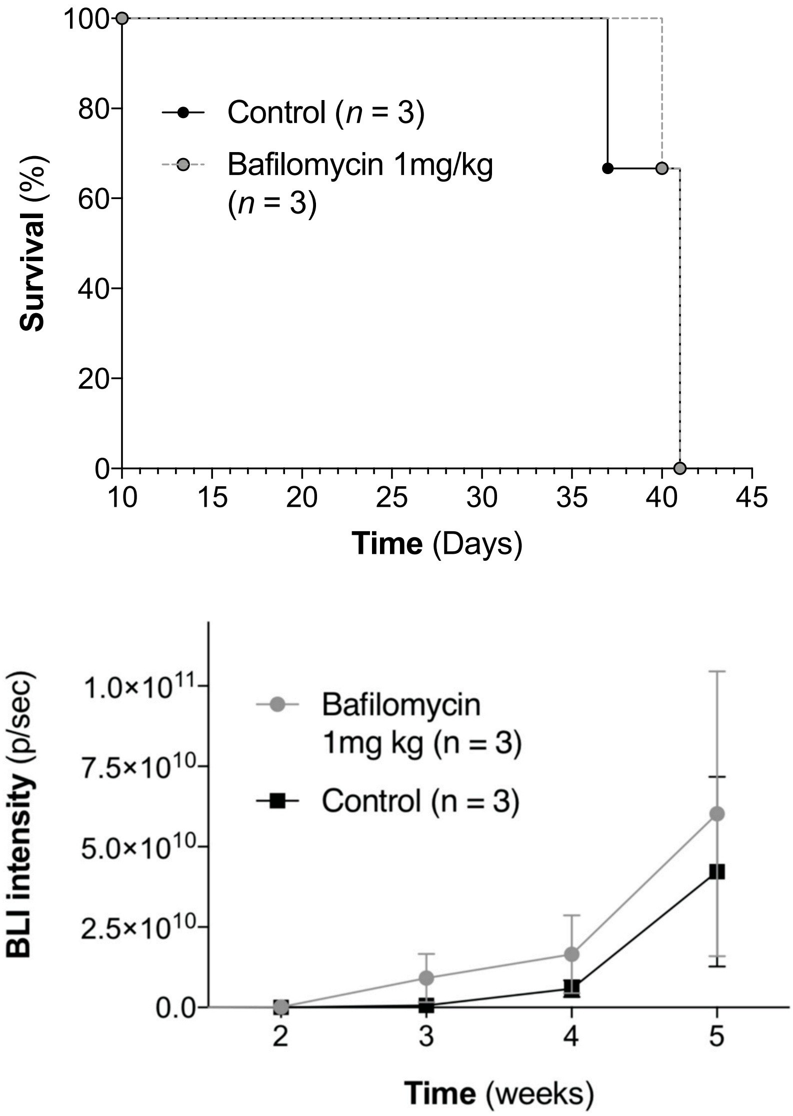

Figure S11. The effect of bafilomycin on xenografted MV4-11 AML cells, studies of survival and disease burden as determined by bioluminescence (BLI) intensity, photons per second (p/sec).

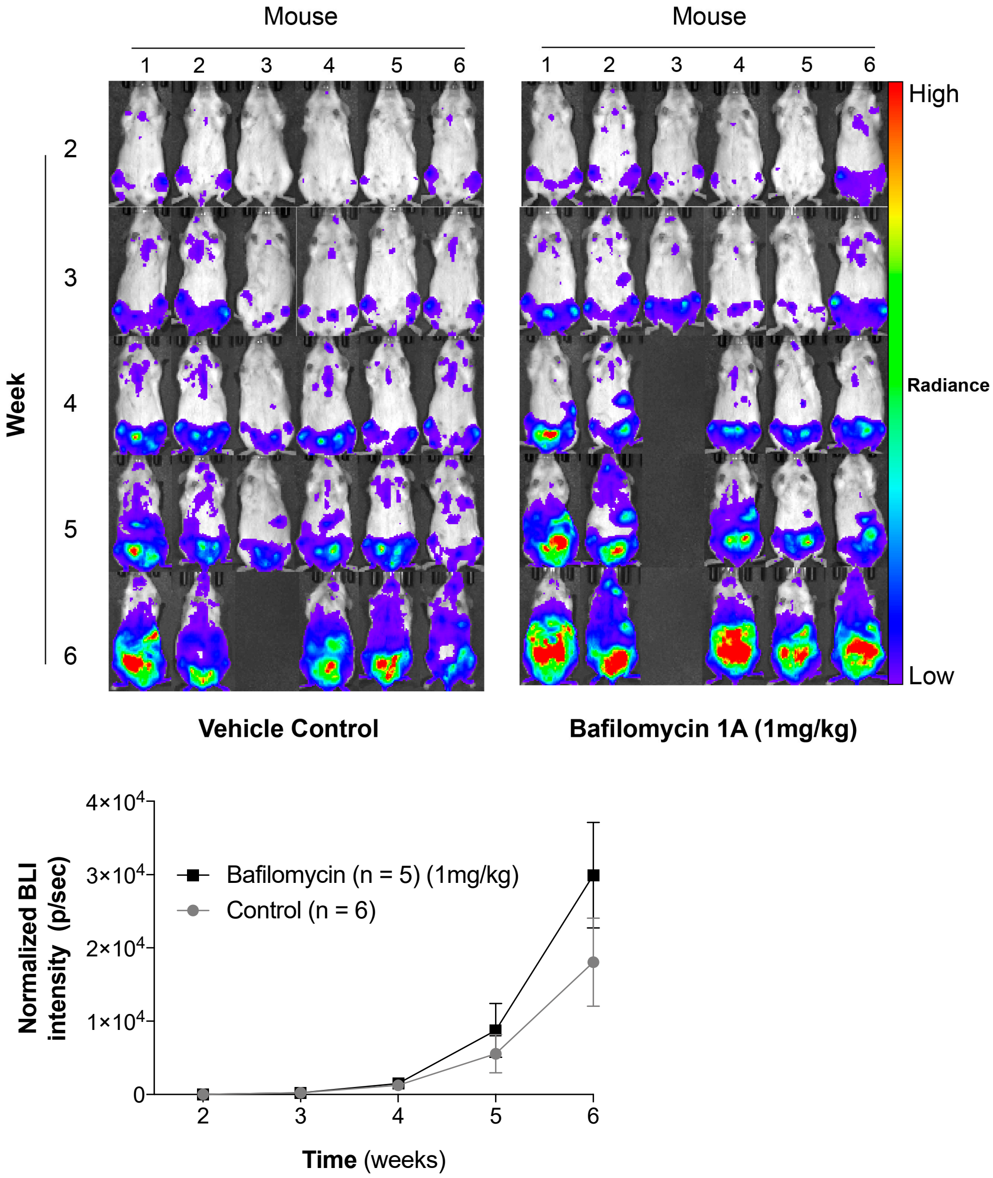

Figure S12. The effect of bafilomycin on bioluminescence imaging of xenografted HL-60 AML cells. No statistical significance was observed in whole body bioluminescence (BLI), photons per second (p/sec) between bafilomycin monotherapy and control mice (p = 0.6570).
